# Supplementary material for: Diastereoselective Synthesis of Dispiro[Imidazothiazolotriazine-Pyrrolidin-Oxindoles] and Their Isomerization Pathways in Basic Medium
Source: Int J Mol Sci. 2023 Nov 15;24(22):16359. doi: 10.3390/ijms242216359 (PMC10671214; doi:10.3390/ijms242216359)

Supplementary Information

Diastereoselective Synthesis of Dispiro[Imidazothiazolotriazine-Pyrrolidin-Oxindoles] and Their Isomerization Pathways in Basic Medium

*Alexei N. Izmest'ev [1,2], Dmitry B. Vinogradov [1], Angelina N. Kravchenko [1], Natalya G. Kolotyrkina [1] and Galina A. Gazieva [1],**

[1] N. D. Zelinsky Institute of Organic Chemistry, Russian Academy of Sciences, 47 Leninsky Prosp., Moscow 119991, Russia; nebeli@mail.ru (A.N.I.); vinogradovdima14@ioc.ac.ru (D.B.V.); kani@ioc.ac.ru (A.N.K.); nkolotyr@gmail.com (N.G.K.)

[2] Department of General and Inorganic Chemistry, National University of Science and Technology "MISIS", 4 Leninsky Prosp., Moscow 119049, Russia

** Correspondence: gaz@ioc.ac.ru*

Table of Contents

1. X-ray crystallographic data and refinement details for **4f**, **5b**, **6a** and **11e** S2
2. References S3
3. Spectra ^{1}H and ^{13}C NMR of compounds **4a-m** S4
4. Spectra ^{1}H and ^{13}C NMR of compounds **5a-j** S28
5. Spectra ^{1}H and ^{13}C NMR of compounds **6a,b,e-g,i,j** S48
6. Spectra ^{1}H and ^{13}C NMR of compounds **11a-f** S62
7. Spectra {^{1}H-^{1}H} NOESY and {^{1}H-^{13}C} HMBC NMR of compound **6a** S74
8. Chromatograms of the mixture of diastereomers **4l** and **4m** S76

X-ray crystallographic data and refinement details for **4f**, **5b**, **6a** and **11e**

Single crystals of **4f, 5b, 6a** and **7e** were grown from MeCN.

Data collection for samples **4f** and **6a** X-ray were collected at 100K on a four-circle Rigaku Synergy S diffractometer equipped with a HyPix600HE area-detector (kappa geometry, shutterless ω-scan technique), using graphite monochromatized Cu K_{α}-radiation. The intensity data were integrated and corrected for absorption and decay by the CrysAlisPro program.[1] The structure was solved by direct methods using SHELXT[2] and refined on F^2 using SHELXL-2018[3] in the OLEX2 program.[4] All non-hydrogen atoms were refined with individual anisotropic displacement parameters. Locations of amino hydrogen atoms (9-NH and 1″-NH) were found from the electron density-difference map; these hydrogen atoms were refined with individual isotropic displacement parameters. All other hydrogen atoms were placed in ideal calculated positions and refined as riding atoms with relative isotropic displacement parameters. The SHELXTL program suite[5] was used for molecular graphics. The crystal of compound **6a** contains disordered solvent molecules in the voids with volume of 182 Å per unit cell. Presumably, it includes an acetonitrile molecule with approximately 85% occupancy and probably water molecules with small partial occupancies, located at the same place, which does not allow correct modeling of the lattice solvent molecules in the crystal. A solvent mask was used by OLEX2[4] to remove solvent molecules (48 electrons were found).

Data collection for samples **5b** and **11e** X-ray were collected at 100K on a Bruker Quest D8 diffractometer equipped with a Photon-III area-detector (graphite monochromator, shutterless φ- and ω-scan technique), using Mo K_{α}-radiation. The intensity data were integrated by the SAINT program[5] and were corrected for absorption and decay using SADABS.[6] The structure was solved by direct methods using SHELXT[2] and refined on F^2 using SHELXL-2018.[3] All non-hydrogen atoms were refined with individual anisotropic displacement parameters. Locations of amino hydrogen atoms (4-NH, 9-NH and 1″-NH) were found from the electron density-difference map; these hydrogen atoms were refined with individual isotropic displacement parameters. All other hydrogen atoms were placed in ideal calculated positions and refined as riding atoms with relative isotropic displacement parameters. The SHELXTL program suite[5] was used for molecular graphics.

Deposition Numbers 2297041–2297044 contain the supplementary crystallographic data for this paper. These data are provided free of charge by the joint Cambridge Crystallographic Data Centre and Fachinformationszentrum Karlsruhe Access Structures service www.ccdc.cam.ac.uk/structures.

References

1. CrysAlisPro. Version 1.171.41.106a. *Rigaku Oxford Diffraction*, **2021**.
2. Sheldrick G. M. SHELXT — Integrated space-group and crystal-structure determination *Acta Cryst.* **2015**, A71, 3–8. http://doi.org/10.1107/S2053273314026370
3. Sheldrick G. M. Crystal structure refinement with SHELXL *Acta Cryst.* **2015**, C71, 3–8. http://doi.org/10.1107/S2053229614024218
4. Dolomanov O. V., Bourhis L. J., Gildea R. J., Howard J. A. K., Puschmann H. OLEX2: a complete structure solution, refinement and analysis program *J. Appl. Cryst.* **2009**, 42, 229–341. http://doi.org/10.1107/S0021889808042726
5. Bruker. APEX-III. *Bruker AXS Inc.*, Madison, Wisconsin, USA, **2019**.
6. Krause L., Herbst-Irmer R., Sheldrick G. M., Stalke D. Comparison of silver and molybdenum microfocus X-ray sources for single-crystal structure determination *J. Appl. Cryst.* **2015**, 48, 3–10. http://doi.org/10.1107/S1600576714022985

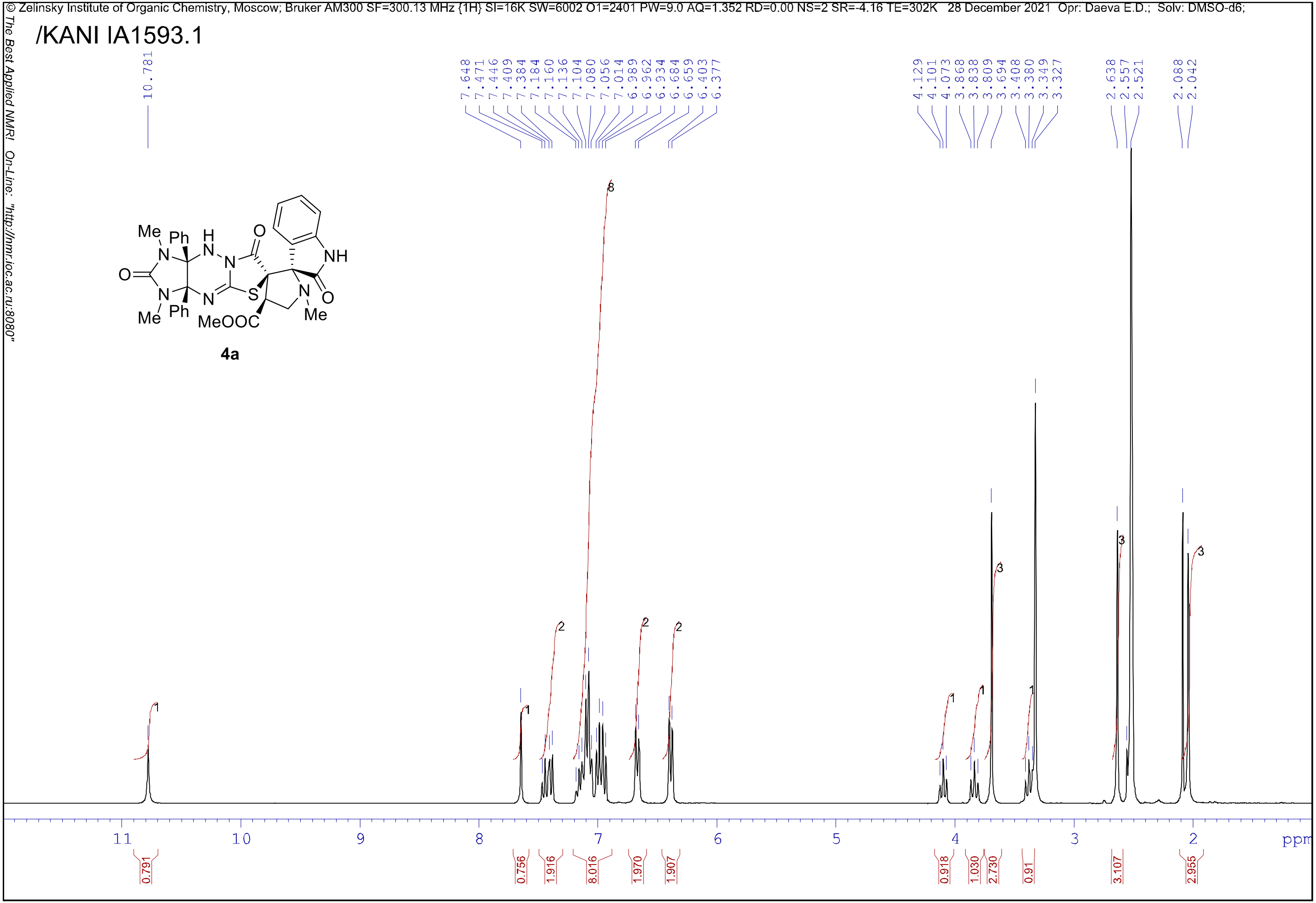
© Zelinsky Institute of Organic Chemistry, Moscow; Bruker AM300 SF=300.13 MHz {1H} SI=16K SW=6002 O1=2401 PW=9.0 AQ=1.352 RD=0.00 NS=2 SR=-4.16 TE=302K 28 December 2021 Opr: Daeva E.D.; Solv: DMSO-d6;
The Best Applied NMR! On-Line: "http://nmr.ioc.ac.ru:8080"
/KANI IA1593.1
10.781
7.648 7.471 7.446 7.409 7.384 7.184 7.160 7.136 7.104 7.080 7.056 7.014 6.989 6.962 6.934 6.684 6.659 6.403 6.377
4.129 4.101 4.073 3.868 3.838 3.809 3.694 3.408 3.380 3.349 3.327
2.638 2.557 2.521
2.088 2.042
4a
ppm
0.791 0.756 1.916 8.016 1.970 1.907 0.918 1.030 2.730 0.91 3.107 2.955

© Zelinsky Institute of Organic Chemistry, Moscow; Bruker AM300 SF=75.47 MHz {13C} SI=32K SW=18114 O1=8301 PW=14.3 AQ=0.901 RD=1.00 NS=322 SR=40.06 TE=301K 17 December 2020 Opr: Daeva E.D.; Solv: DMSO-d6;
The Best Applied NMR! On-Line: "http://nmr.ioc.ac.ru:8080"

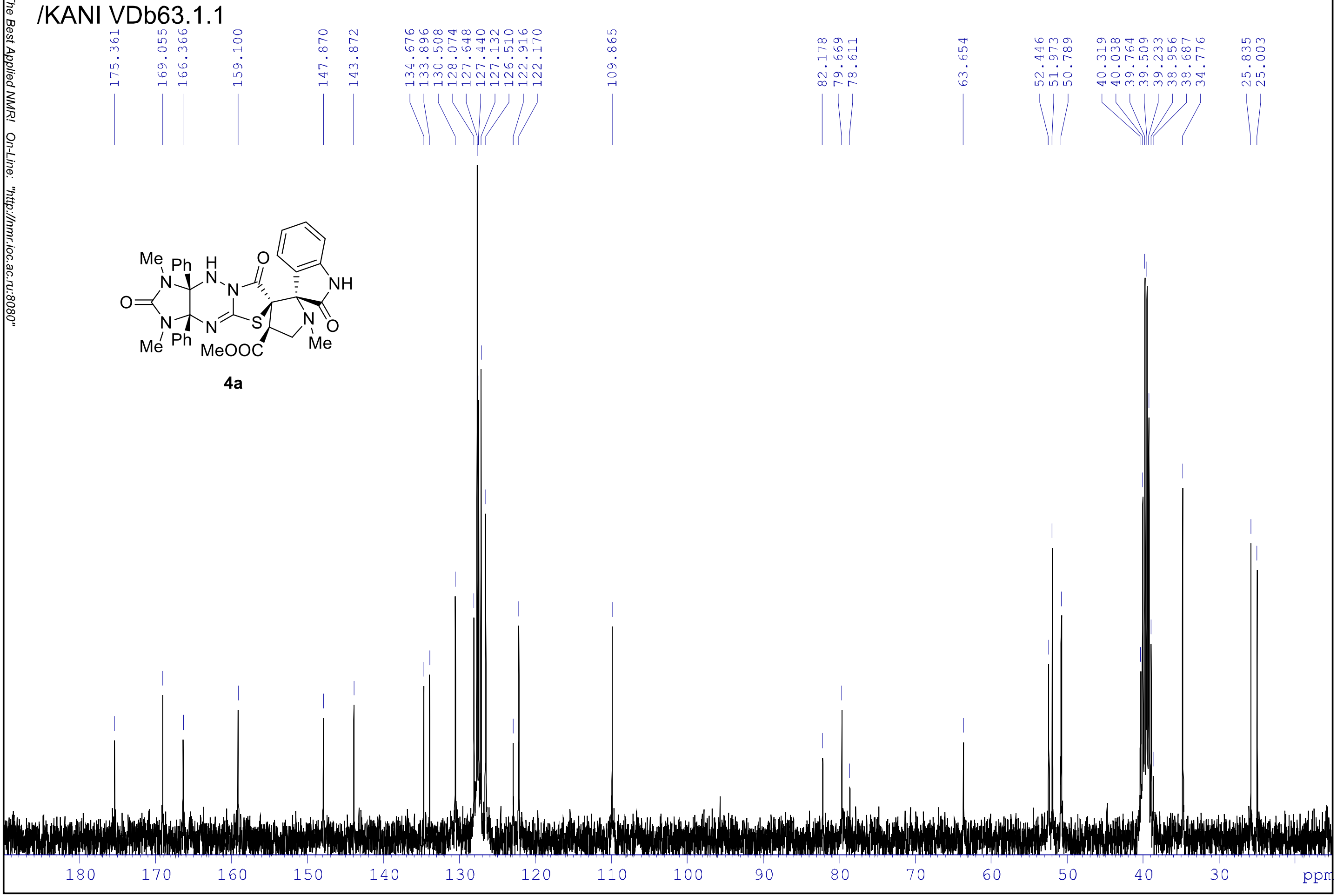

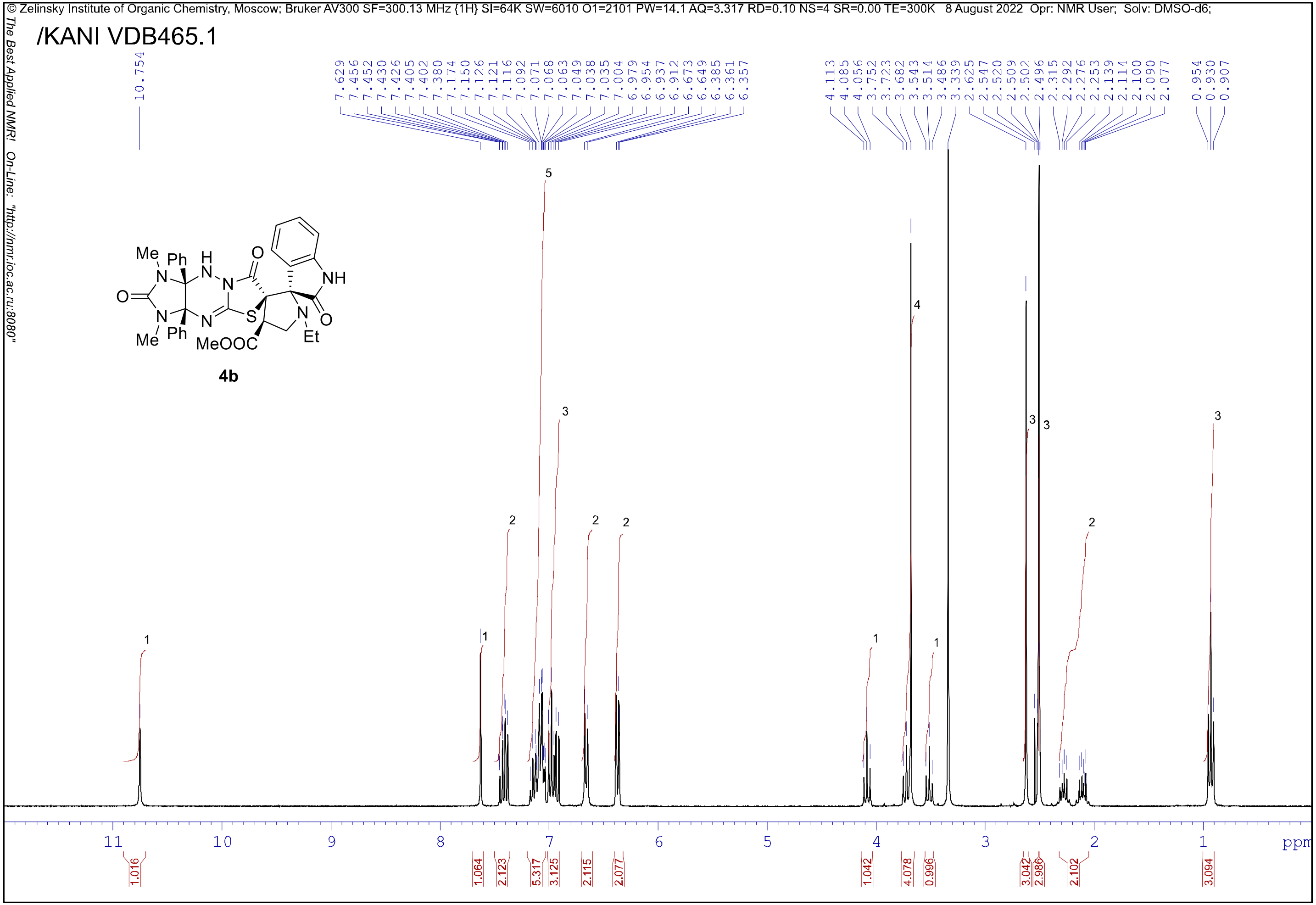
© Zelinsky Institute of Organic Chemistry, Moscow; Bruker AV300 SF=300.13 MHz {1H} SI=64K SW=6010 O1=2101 PW=14.1 AQ=3.317 RD=0.10 NS=4 SR=0.00 TE=300K 8 August 2022 Opr: NMR User; Solv: DMSO-d6;
/KANI VDB465.1
The Best Applied NMR! On-Line: "http://nmr.ioc.ac.ru:8080"
10.754
7.629 7.456 7.452 7.430 7.426 7.405 7.402 7.380 7.174 7.150 7.126 7.121 7.116 7.092 7.071 7.068 7.063 7.049 7.038 7.035 7.004 6.979 6.954 6.937 6.912 6.673 6.649 6.385 6.361 6.357
4.113 4.085 4.056 3.752 3.723 3.682 3.543 3.514 3.486 3.339 2.625 2.547 2.520 2.509 2.502 2.496 2.315 2.292 2.276 2.253 2.139 2.114 2.100 2.090 2.077
0.954 0.930 0.907
Me
Ph
H
N
O
NH
S
Et
MeOOC
4b
ppm
1.016 1.064 2.123 5.317 3.125 2.115 2.077 1.042 4.078 0.996 3.042 2.986 2.102 3.094

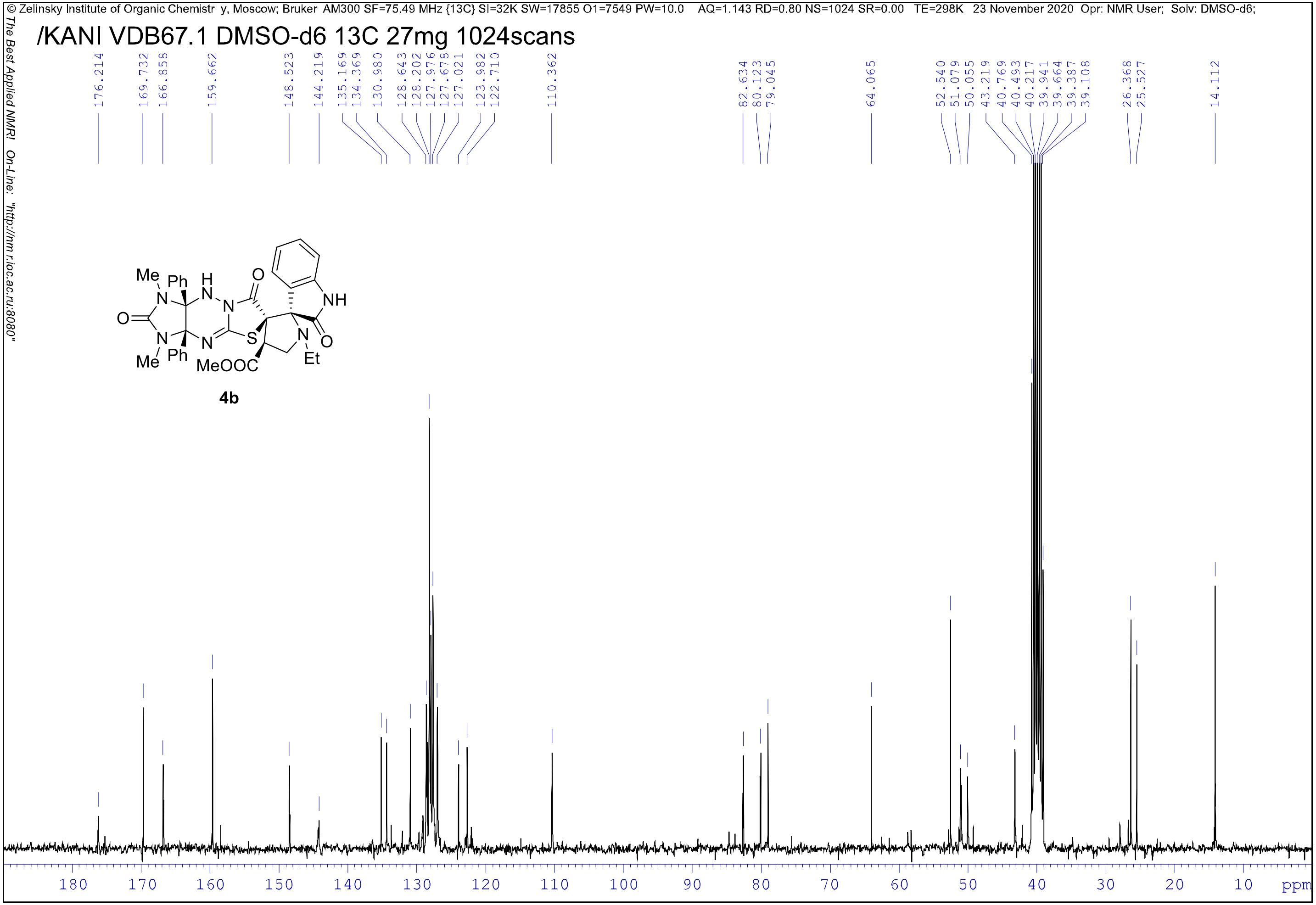
© Zelinsky Institute of Organic Chemistr y, Moscow; Bruker AM300 SF=75.49 MHz {13C} SI=32K SW=17855 O1=7549 PW=10.0 AQ=1.143 RD=0.80 NS=1024 SR=0.00 TE=298K 23 November 2020 Opr: NMR User; Solv: DMSO-d6;
/KANI VDB67.1 DMSO-d6 13C 27mg 1024scans
The Best Applied NMR! On-Line: "http://nmr.ioc.ac.ru:8080"
176.214
169.732
166.858
159.662
148.523
144.219
135.169
134.369
130.980
128.643
128.202
127.976
127.678
127.021
123.982
122.710
110.362
82.634
80.123
79.045
64.065
52.540
51.079
50.055
43.219
40.769
40.493
40.217
39.941
39.664
39.387
39.108
26.368
25.527
14.112
Me
Ph
H
N
O
NH
MeOOC
Et
S
4b
180 170 160 150 140 130 120 110 100 90 80 70 60 50 40 30 20 10 ppm

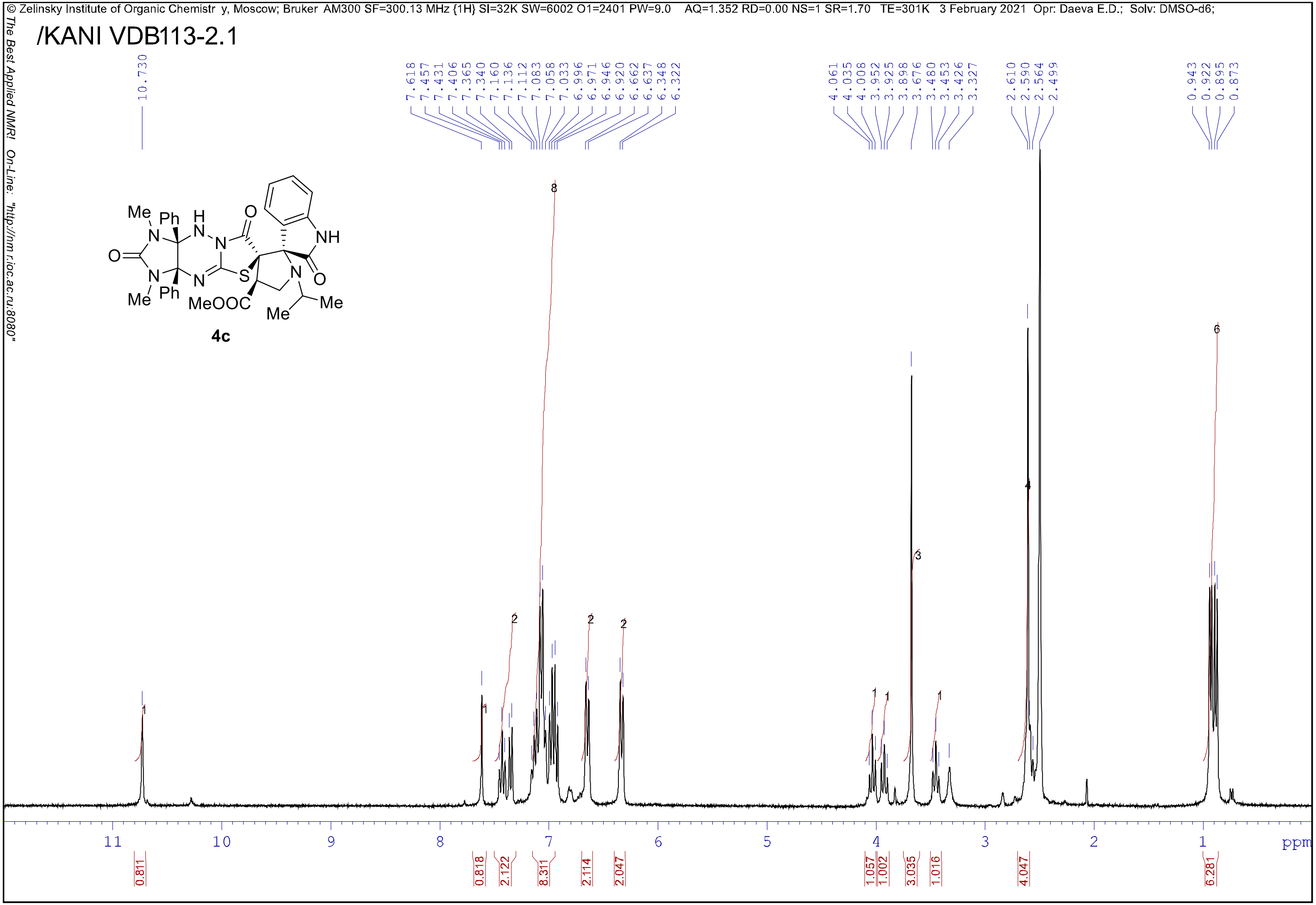
© Zelinsky Institute of Organic Chemistr y, Moscow; Bruker AM300 SF=300.13 MHz {1H} SI=32K SW=6002 O1=2401 PW=9.0 AQ=1.352 RD=0.00 NS=1 SR=1.70 TE=301K 3 February 2021 Opr: Daeva E.D.; Solv: DMSO-d6;
The Best Applied NMR! On-Line: "http://nmr.ioc.ac.ru:8080"
/KANI VDB113-2.1
10.730
7.618 7.457 7.431 7.406 7.365 7.340 7.160 7.136 7.112 7.083 7.058 7.033 6.996 6.971 6.946 6.920 6.662 6.637 6.348 6.322
4.061 4.035 4.008 3.952 3.925 3.898 3.676 3.480 3.453 3.426 3.327
2.610 2.590 2.564 2.499
0.943 0.922 0.895 0.873
Me
Ph
H
O
N
N
N
NH
O
N
N
S
N
O
Me
Ph
MeOOC
Me
Me
4c
0.811 0.818 2.122 8.311 2.114 2.047 1.057 1.002 3.035 1.016 4.047 6.281
11 10 9 8 7 6 5 4 3 2 1 ppm

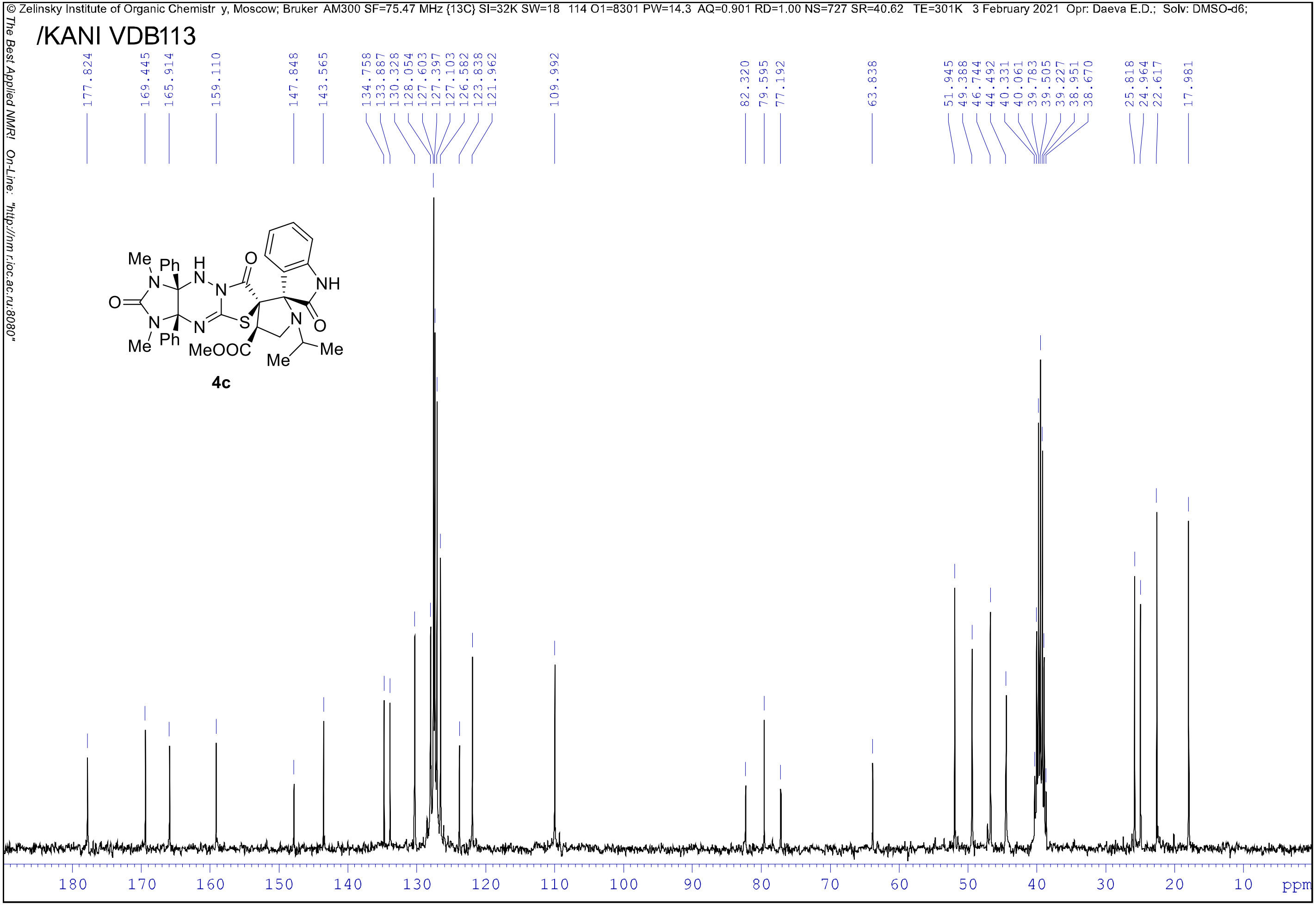
© Zelinsky Institute of Organic Chemistr y, Moscow; Bruker AM300 SF=75.47 MHz {13C} SI=32K SW=18 114 O1=8301 PW=14.3 AQ=0.901 RD=1.00 NS=727 SR=40.62 TE=301K 3 February 2021 Opr: Daeva E.D.; Solv: DMSO-d6;
The Best Applied NMR! On-Line: "http://nmr.ioc.ac.ru:8080"
/KANI VDB113
177.824
169.445
165.914
159.110
147.848
143.565
134.758
133.887
130.328
128.054
127.603
127.397
127.103
126.582
123.838
121.962
109.992
82.320
79.595
77.192
63.838
51.945
49.388
46.744
44.492
40.331
40.061
39.783
39.505
39.227
38.951
38.670
25.818
24.964
22.617
17.981
4c
180 170 160 150 140 130 120 110 100 90 80 70 60 50 40 30 20 10 ppm

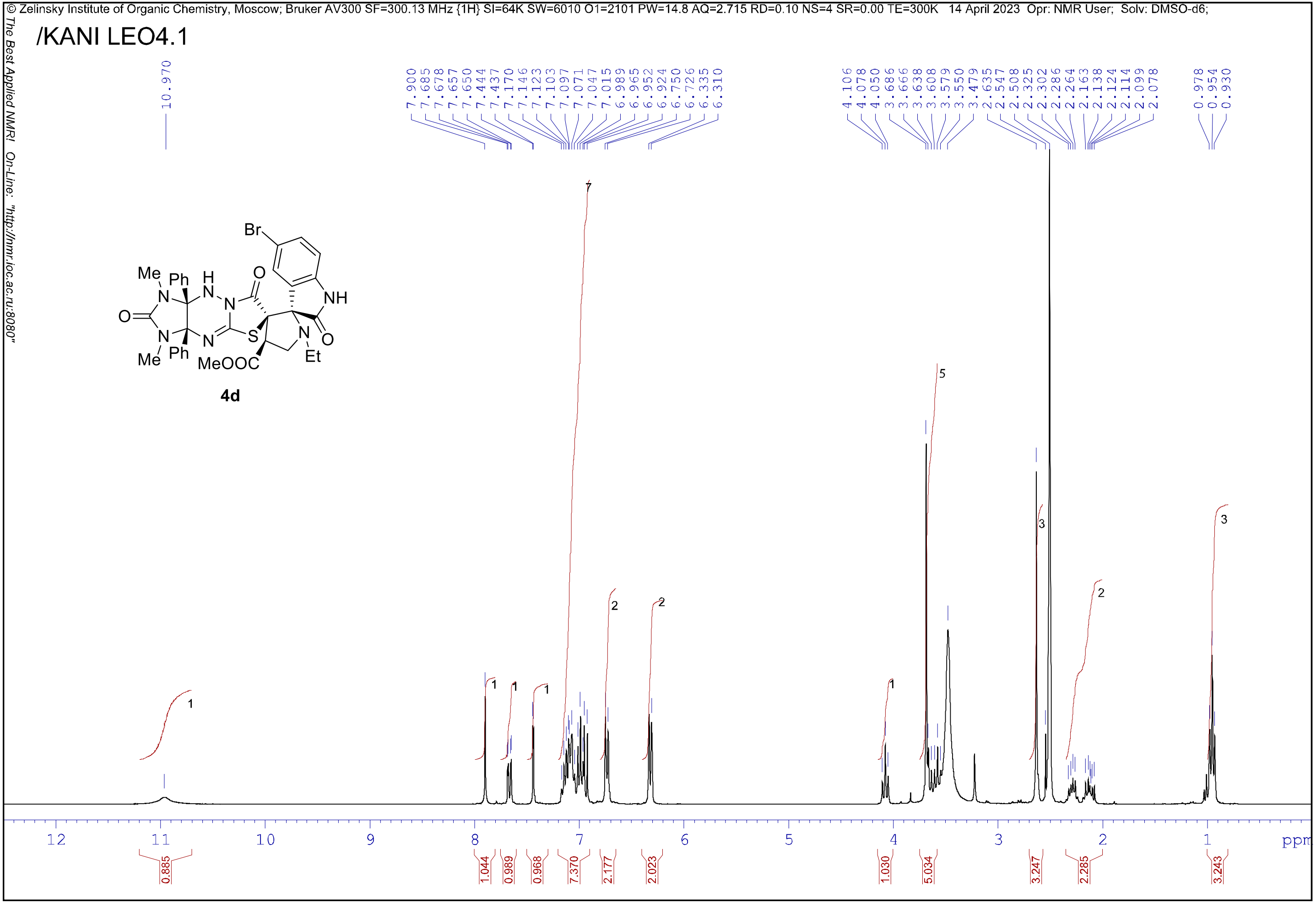
© Zelinsky Institute of Organic Chemistry, Moscow; Bruker AV300 SF=300.13 MHz {1H} SI=64K SW=6010 O1=2101 PW=14.8 AQ=2.715 RD=0.10 NS=4 SR=0.00 TE=300K 14 April 2023 Opr: NMR User; Solv: DMSO-d6;
The Best Applied NMR! On-Line: "http://nmr.ioc.ac.ru:8080"
/KANI LEO4.1
10.970
7.900 7.685 7.678 7.657 7.650 7.444 7.437 7.170 7.146 7.123 7.103 7.097 7.071 7.047 7.015 6.989 6.965 6.952 6.924 6.750 6.726 6.335 6.310
4.106 4.078 4.050 3.686 3.666 3.638 3.608 3.579 3.550 3.479 2.635 2.547 2.508 2.325 2.302 2.286 2.264 2.163 2.138 2.124 2.114 2.099 2.078
0.978 0.954 0.930
4d
0.885 1.044 0.989 0.968 7.370 2.177 2.023 1.030 5.034 3.247 2.285 3.243
ppm

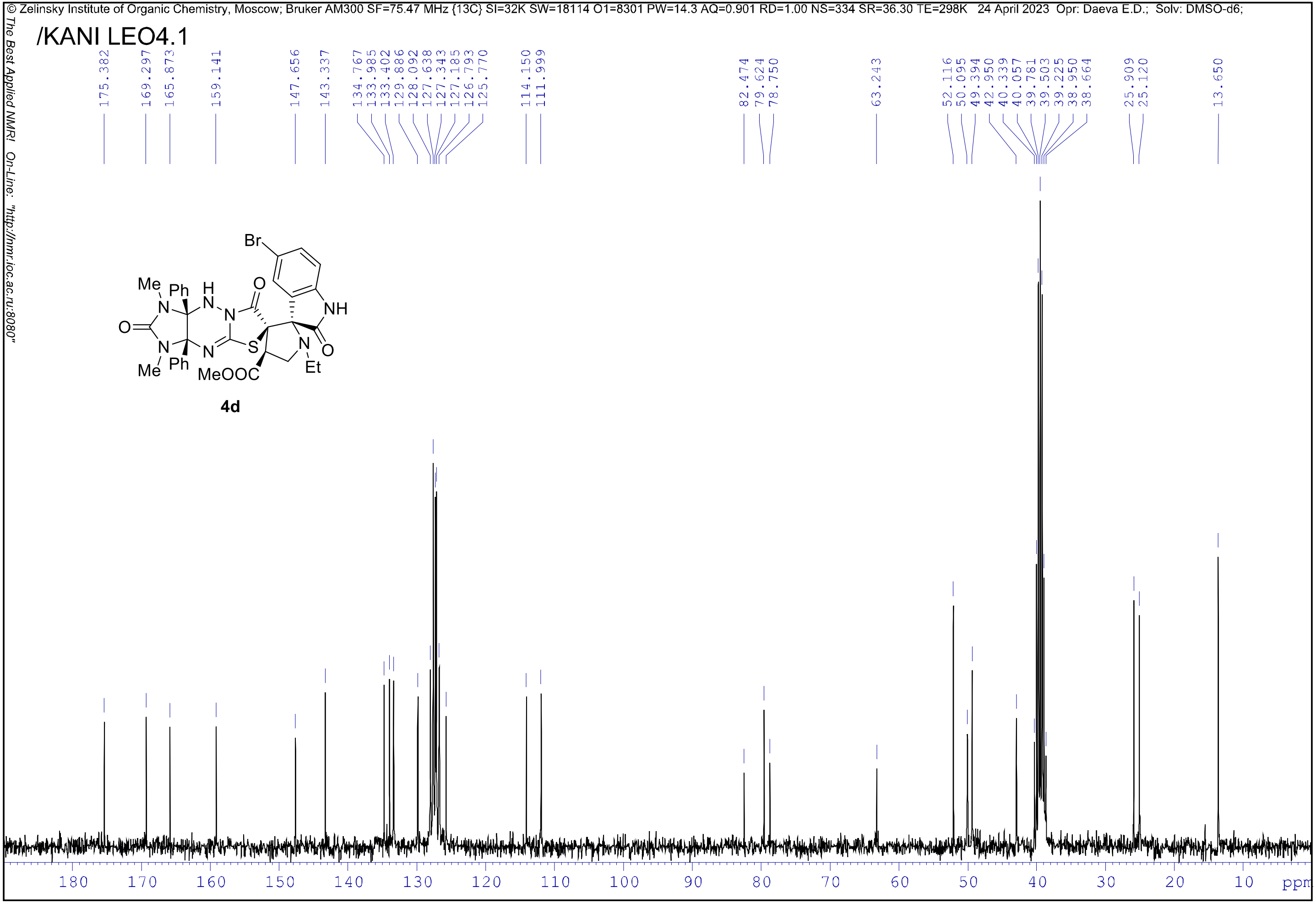
© Zelinsky Institute of Organic Chemistry, Moscow; Bruker AM300 SF=75.47 MHz {13C} SI=32K SW=18114 O1=8301 PW=14.3 AQ=0.901 RD=1.00 NS=334 SR=36.30 TE=298K 24 April 2023 Opr: Daeva E.D.; Solv: DMSO-d6;
The Best Applied NMR! On-Line: "http://nmr.ioc.ac.ru:8080"
/KANI LEO4.1
175.382
169.297
165.873
159.141
147.656
143.337
134.767
133.985
133.402
129.886
128.092
127.638
127.343
127.185
126.793
125.770
114.150
111.999
82.474
79.624
78.750
63.243
52.116
50.095
49.394
42.950
40.339
40.057
39.781
39.503
39.225
38.950
38.664
25.909
25.120
13.650
Br
Me
Ph
H
N
O
NH
S
Et
MeOOC
4d
180
170
160
150
140
130
120
110
100
90
80
70
60
50
40
30
20
10
ppm

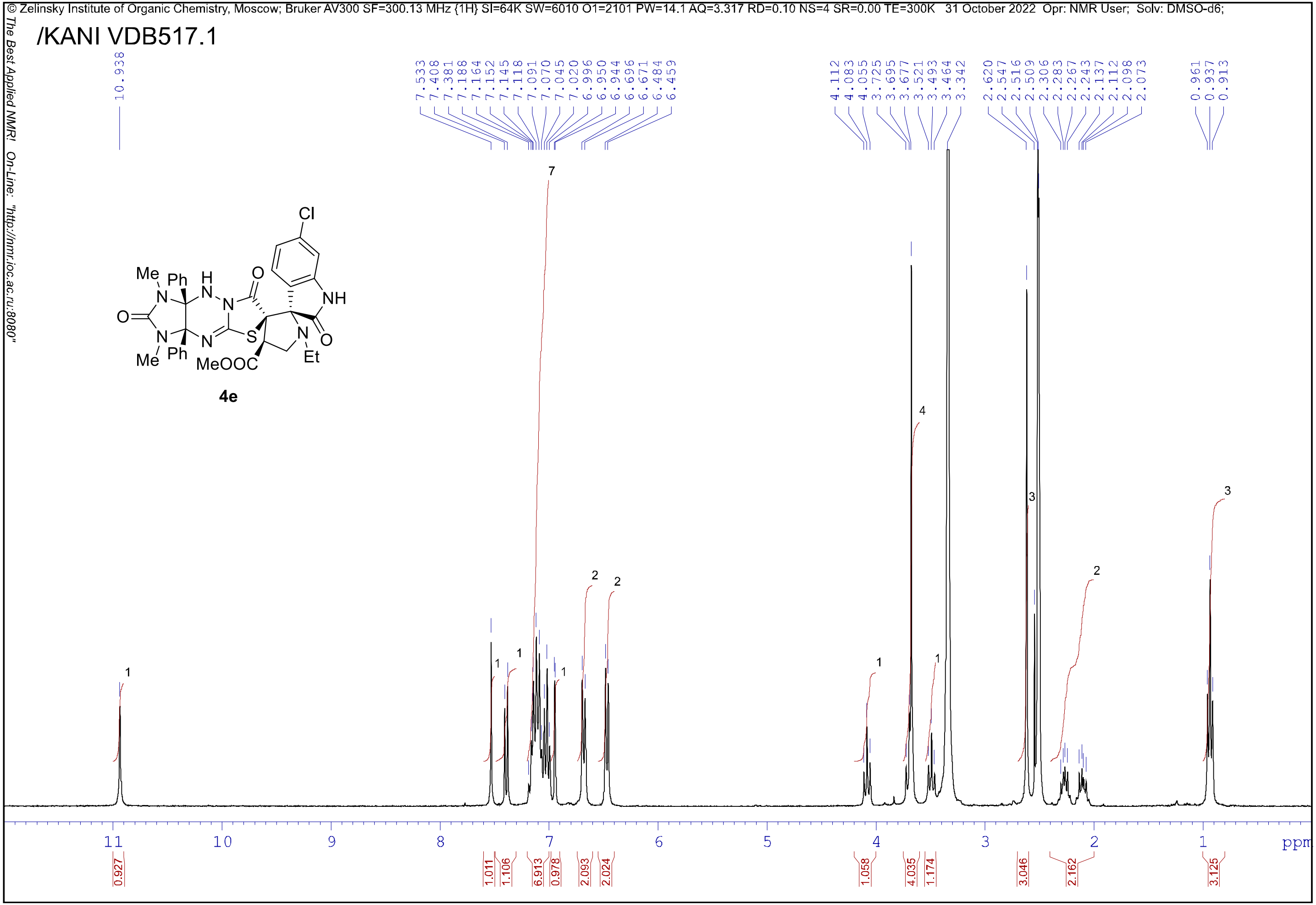
© Zelinsky Institute of Organic Chemistry, Moscow; Bruker AV300 SF=300.13 MHz {1H} SI=64K SW=6010 O1=2101 PW=14.1 AQ=3.317 RD=0.10 NS=4 SR=0.00 TE=300K 31 October 2022 Opr: NMR User; Solv: DMSO-d6;
The Best Applied NMR! On-Line: "http://nmr.ioc.ac.ru:8080"
/KANI VDB517.1
10.938
7.533 7.408 7.381 7.188 7.164 7.152 7.145 7.118 7.091 7.070 7.045 7.020 6.996 6.950 6.944 6.696 6.671 6.484 6.459
4.112 4.083 4.055 3.725 3.695 3.677 3.521 3.493 3.464 3.342
2.620 2.547 2.516 2.509 2.306 2.283 2.267 2.243 2.137 2.112 2.098 2.073
0.961 0.937 0.913
4e
11 10 9 8 7 6 5 4 3 2 1 ppm
0.927 1.011 1.106 6.913 0.978 2.093 2.024 1.058 4.035 1.174 3.046 2.162 3.125

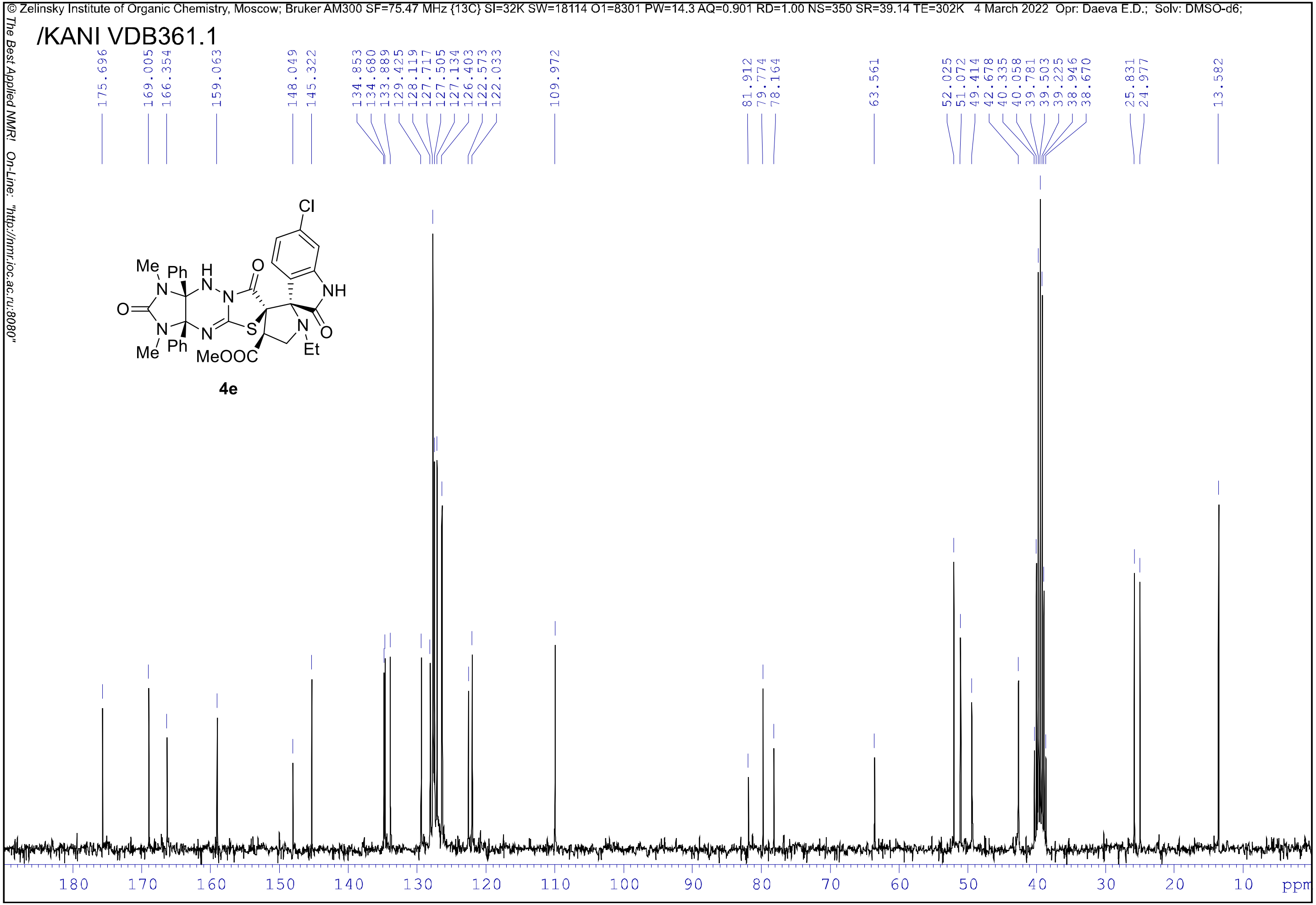
© Zelinsky Institute of Organic Chemistry, Moscow; Bruker AM300 SF=75.47 MHz {13C} SI=32K SW=18114 O1=8301 PW=14.3 AQ=0.901 RD=1.00 NS=350 SR=39.14 TE=302K 4 March 2022 Opr: Daeva E.D.; Solv: DMSO-d6;
The Best Applied NMR! On-Line: "http://nmr.ioc.ac.ru:8080"
/KANI VDB361.1
175.696
169.005
166.354
159.063
148.049
145.322
134.853
134.680
133.889
129.425
128.119
127.717
127.505
127.134
126.403
122.573
122.033
109.972
81.912
79.774
78.164
63.561
52.025
51.072
49.414
42.678
40.335
40.058
39.781
39.503
39.225
38.946
38.670
25.831
24.977
13.582
Cl
Me
Ph
H
N
O
NH
MeOOC
S
Et
4e
180 170 160 150 140 130 120 110 100 90 80 70 60 50 40 30 20 10 ppm

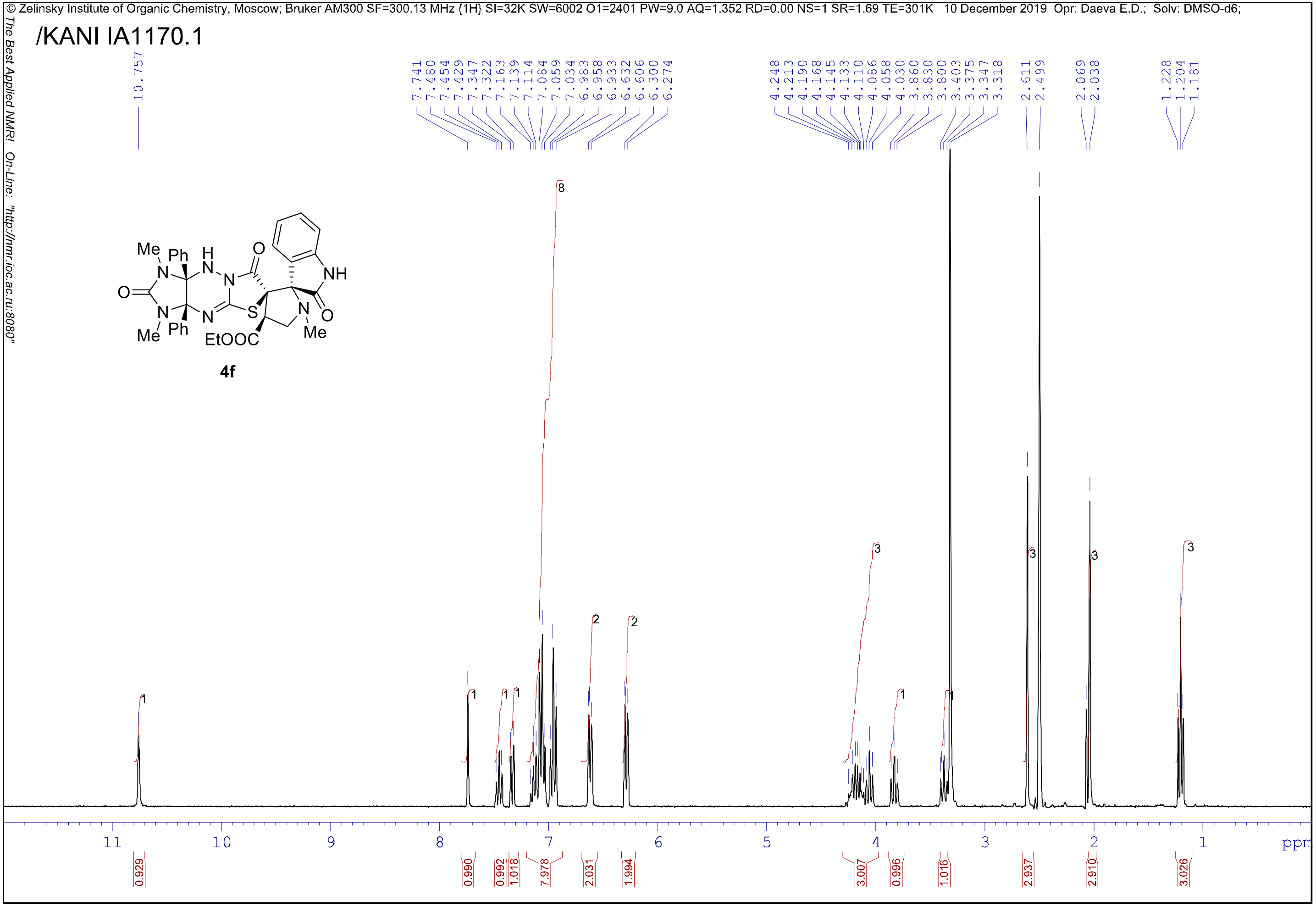
© Zelinsky Institute of Organic Chemistry, Moscow; Bruker AM300 SF=300.13 MHz {1H} SI=32K SW=6002 O1=2401 PW=9.0 AQ=1.352 RD=0.00 NS=1 SR=1.69 TE=301K 10 December 2019 Opr: Daeva E.D.; Solv: DMSO-d6;
The Best Applied NMR! On-Line: "http://nmr.ioc.ac.ru:8080"
/KANI IA1170.1
10.757
7.741 7.480 7.454 7.429 7.347 7.322 7.163 7.139 7.114 7.084 7.059 7.034 6.983 6.958 6.933 6.632 6.606 6.300 6.274
4.248 4.213 4.190 4.168 4.145 4.133 4.110 4.086 4.058 4.030 3.860 3.830 3.800 3.403 3.375 3.347 3.318
2.611 2.499
2.069 2.038
1.228 1.204 1.181
Me
Ph
H
N
O
NH
EtOOC
S
Me
4f
ppm
0.929 0.990 0.992 1.018 7.978 2.031 1.994 3.007 0.996 1.016 2.937 2.910 3.026

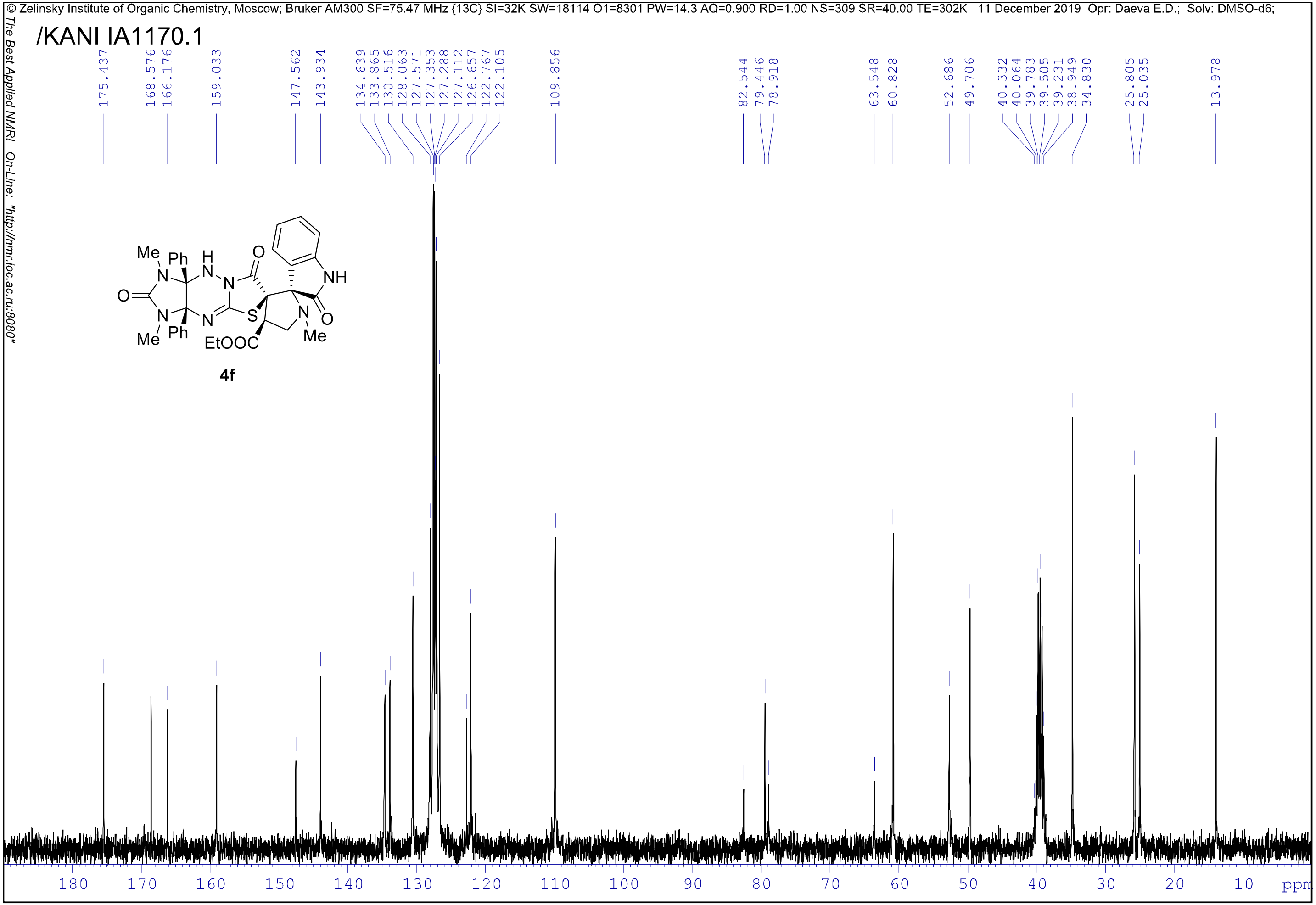
© Zelinsky Institute of Organic Chemistry, Moscow; Bruker AM300 SF=75.47 MHz {13C} SI=32K SW=18114 O1=8301 PW=14.3 AQ=0.900 RD=1.00 NS=309 SR=40.00 TE=302K 11 December 2019 Opr: Daeva E.D.; Solv: DMSO-d6;
The Best Applied NMR! On-Line: "http://nmr.ioc.ac.ru:8080"
/KANI IA1170.1
175.437
168.576
166.176
159.033
147.562
143.934
134.639
133.865
130.516
128.063
127.571
127.353
127.288
127.112
126.657
122.767
122.105
109.856
82.544
79.446
78.918
63.548
60.828
52.686
49.706
40.332
40.064
39.783
39.505
39.231
38.949
34.830
25.805
25.035
13.978
4f
ppm

© Zelinsky Institute of Organic Chemistr y, Moscow; Bruker AM300 SF=300.13 MHz {1H} SI=32K SW=6002 O1=2401 PW=9.0 AQ=1.352 RD=0.00 NS=1 SR=1.69 TE=301K 13 December 2019 Opr: Daeva E.D.; Solv: DMSO-d6;

/KANI IA1175.1

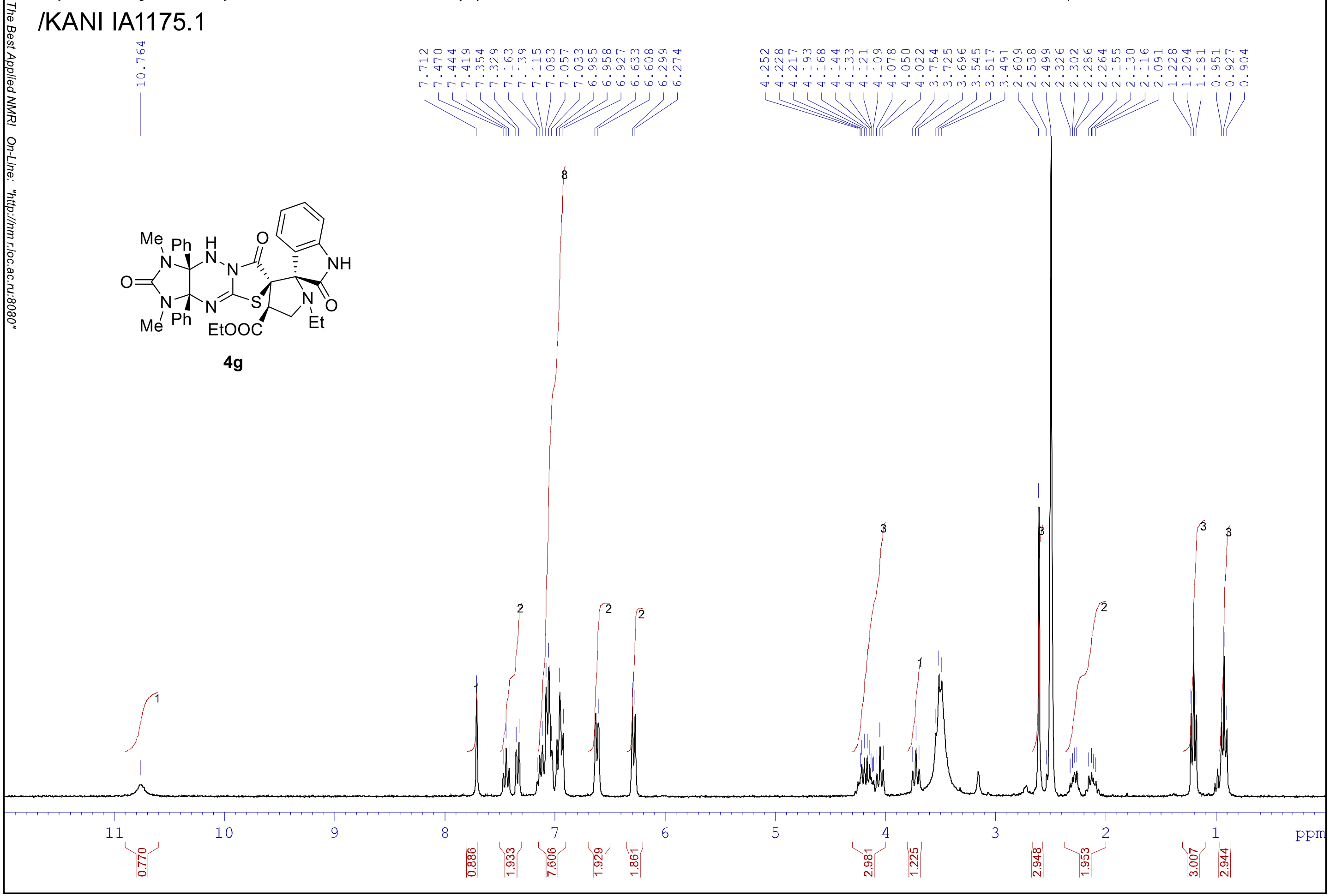

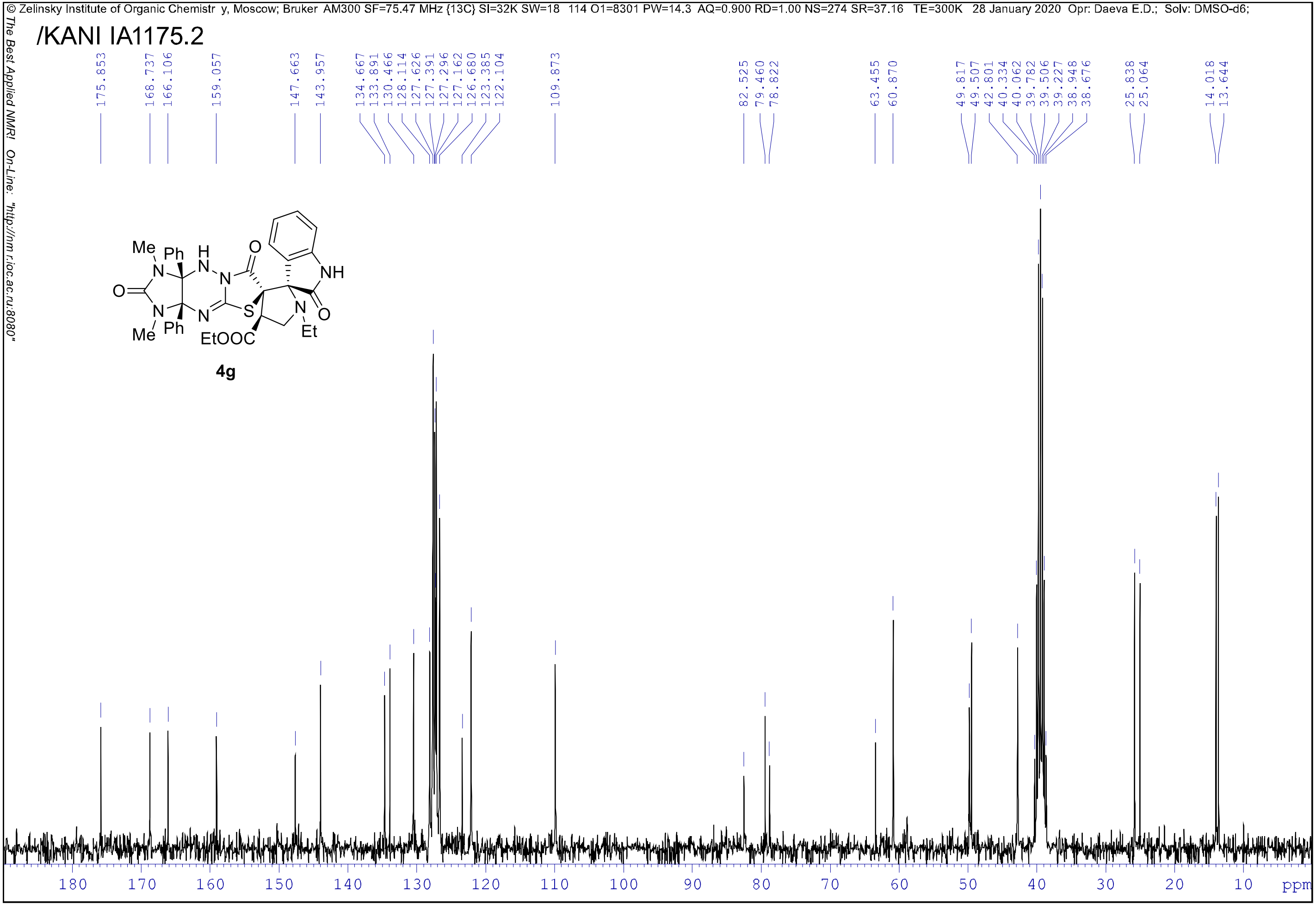
© Zelinsky Institute of Organic Chemistr y, Moscow; Bruker AM300 SF=75.47 MHz {13C} SI=32K SW=18 114 O1=8301 PW=14.3 AQ=0.900 RD=1.00 NS=274 SR=37.16 TE=300K 28 January 2020 Opr: Daeva E.D.; Solv: DMSO-d6;
/KANI IA1175.2
The Best Applied NMR! On-Line: "http://nm r.ioc.ac.ru:8080"
175.853
168.737
166.106
159.057
147.663
143.957
134.667
133.891
130.466
128.114
127.626
127.391
127.296
127.162
126.680
123.385
122.104
109.873
82.525
79.460
78.822
63.455
60.870
49.817
49.507
42.801
40.334
40.062
39.782
39.506
39.227
38.948
38.676
25.838
25.064
14.018
13.644
Me
Ph
H
N
O
N
N
NH
O
N
N
S
N
O
Me
Ph
EtOOC
Et
4g
180
170
160
150
140
130
120
110
100
90
80
70
60
50
40
30
20
10
ppm

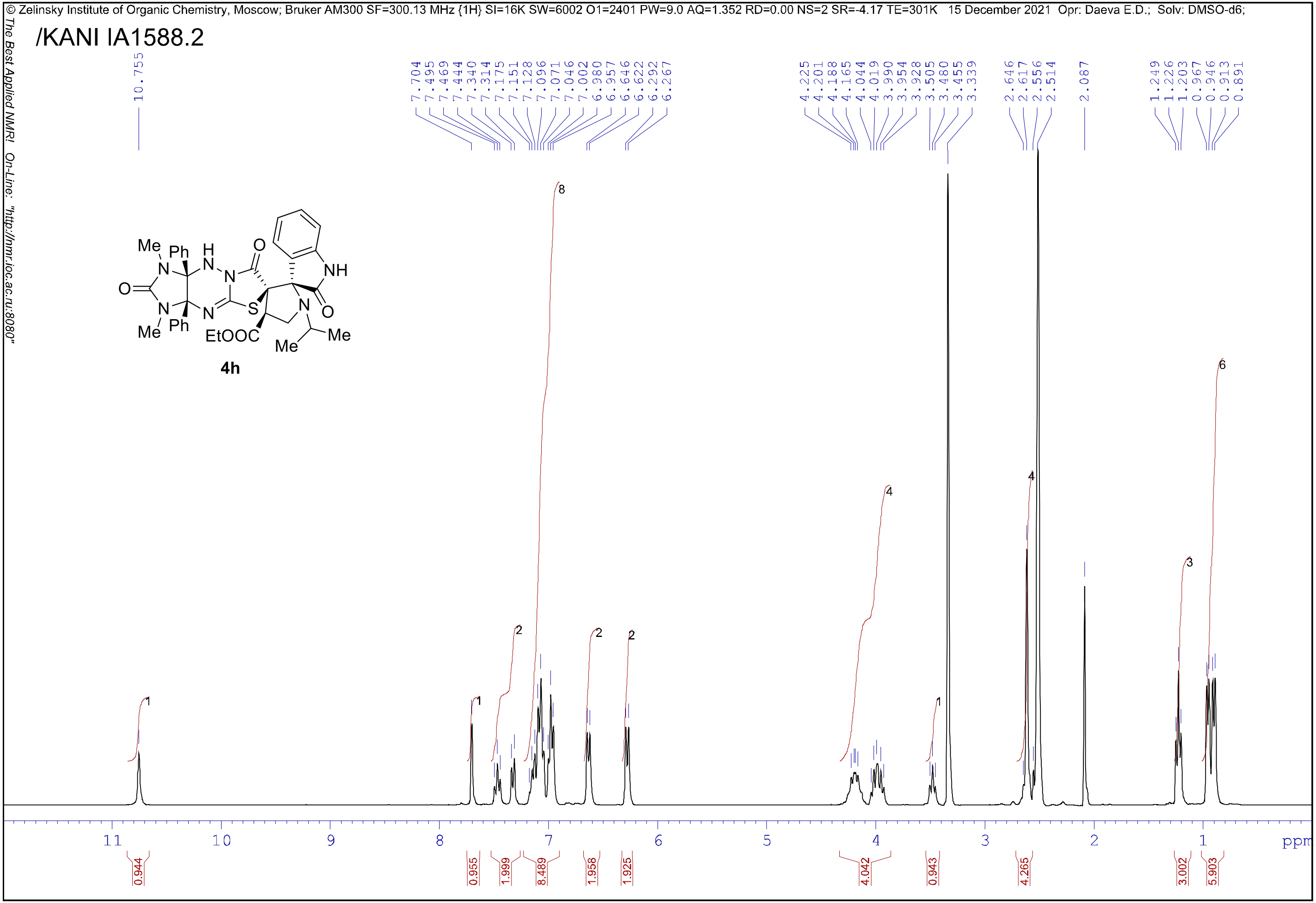
© Zelinsky Institute of Organic Chemistry, Moscow; Bruker AM300 SF=300.13 MHz {1H} SI=16K SW=6002 O1=2401 PW=9.0 AQ=1.352 RD=0.00 NS=2 SR=-4.17 TE=301K 15 December 2021 Opr: Daeva E.D.; Solv: DMSO-d6;
The Best Applied NMR! On-Line: "http://nmr.ioc.ac.ru:8080"
/KANI IA1588.2
10.755
7.704 7.495 7.469 7.444 7.340 7.314 7.175 7.151 7.128 7.096 7.071 7.046 7.002 6.980 6.957 6.646 6.622 6.292 6.267
4.225 4.201 4.188 4.165 4.044 4.019 3.990 3.954 3.928 3.505 3.480 3.455 3.339
2.646 2.617 2.556 2.514
2.087
1.249 1.226 1.203 0.967 0.946 0.913 0.891
4h
0.944 0.955 1.999 8.489 1.958 1.925 4.042 0.943 4.265 3.002 5.903
ppm

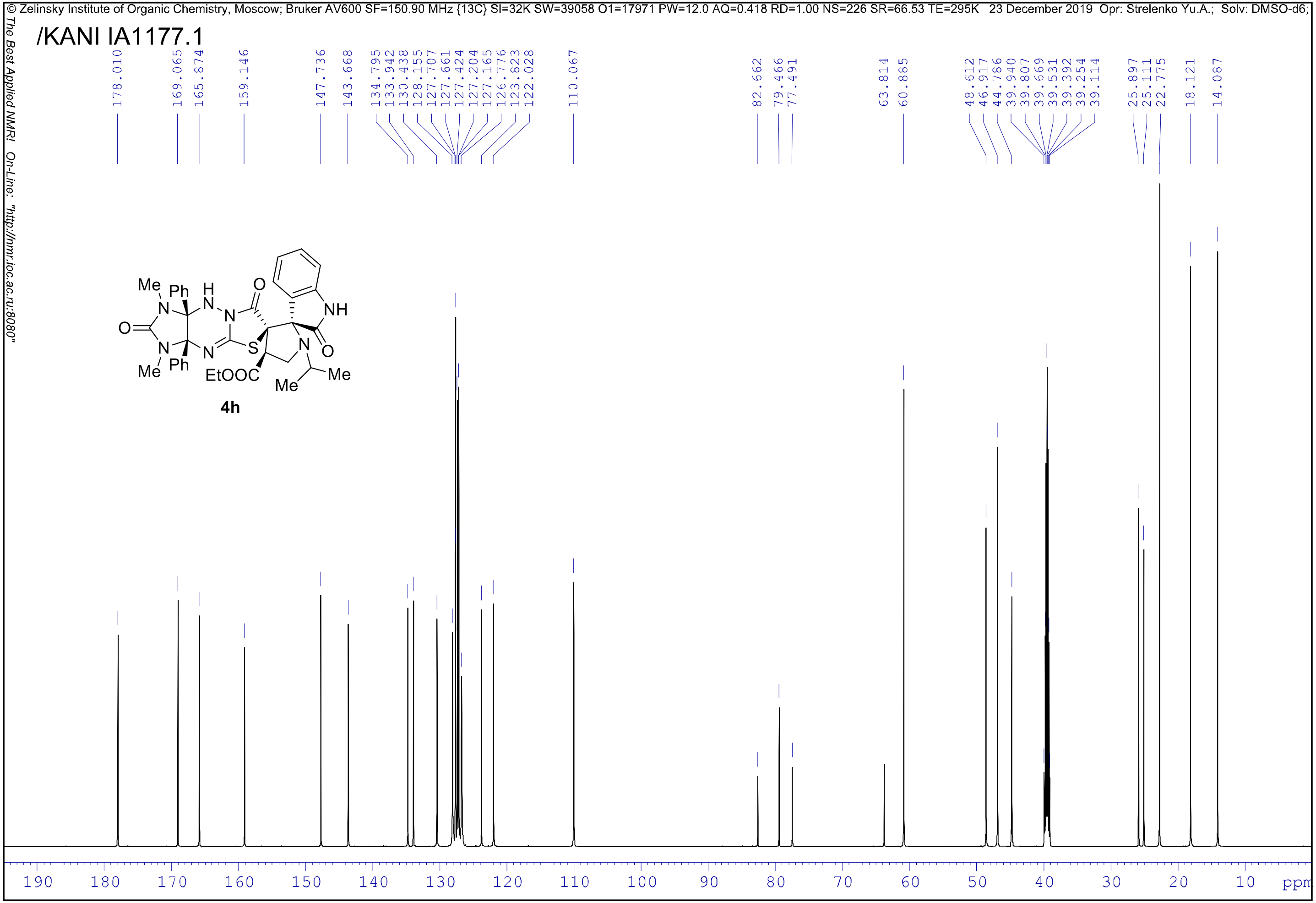
© Zelinsky Institute of Organic Chemistry, Moscow; Bruker AV600 SF=150.90 MHz {13C} SI=32K SW=39058 O1=17971 PW=12.0 AQ=0.418 RD=1.00 NS=226 SR=66.53 TE=295K 23 December 2019 Opr: Strelenko Yu.A.; Solv: DMSO-d6;
The Best Applied NMR! On-Line: "http://nmr.ioc.ac.ru:8080"
/KANI IA1177.1
178.010
169.065
165.874
159.146
147.736
143.668
134.795
133.942
130.438
128.155
127.707
127.661
127.424
127.204
127.165
126.776
123.823
122.028
110.067
82.662
79.466
77.491
63.814
60.885
48.612
46.917
44.786
39.940
39.807
39.669
39.531
39.392
39.254
39.114
25.897
25.111
22.775
18.121
14.087
4h
190 180 170 160 150 140 130 120 110 100 90 80 70 60 50 40 30 20 10 ppm

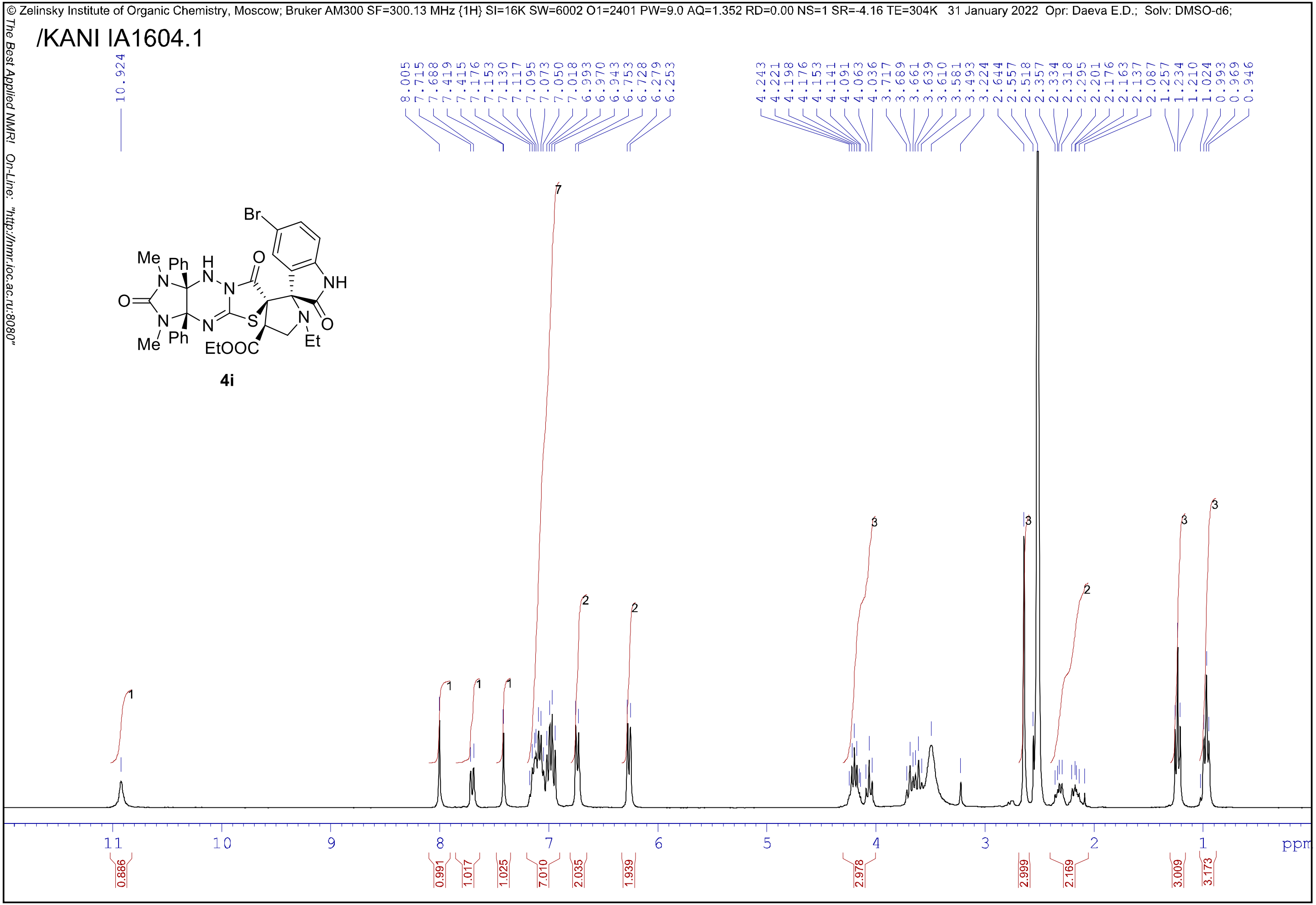

© Zelinsky Institute of Organic Chemistry, Moscow; Bruker AM300 SF=300.13 MHz {1H} SI=16K SW=6002 O1=2401 PW=9.0 AQ=1.352 RD=0.00 NS=1 SR=-4.16 TE=304K 31 January 2022 Opr: Daeva E.D.; Solv: DMSO-d6;
The Best Applied NMR! On-Line: "http://nmr.ioc.ac.ru:8080"
/KANI IA1604.1
10.924
8.005 7.715 7.688 7.419 7.415 7.176 7.153 7.130 7.117 7.095 7.073 7.050 7.018 6.993 6.970 6.943 6.753 6.728 6.279 6.253
4.243 4.221 4.198 4.176 4.153 4.141 4.091 4.063 4.036 3.717 3.689 3.661 3.639 3.610 3.581 3.493 3.224 2.644 2.557 2.518 2.357 2.334 2.318 2.295 2.201 2.176 2.163 2.137 2.087 1.257 1.234 1.210 1.024 0.993 0.969 0.946
Br
Me
Ph
H
N
O
NH
O
N
N
N
S
N
O
N
N
Me
Ph
EtOOC
Et
4i
ppm
0.886 0.991 1.017 1.025 7.010 2.035 1.939 2.978 2.999 2.169 3.009 3.173

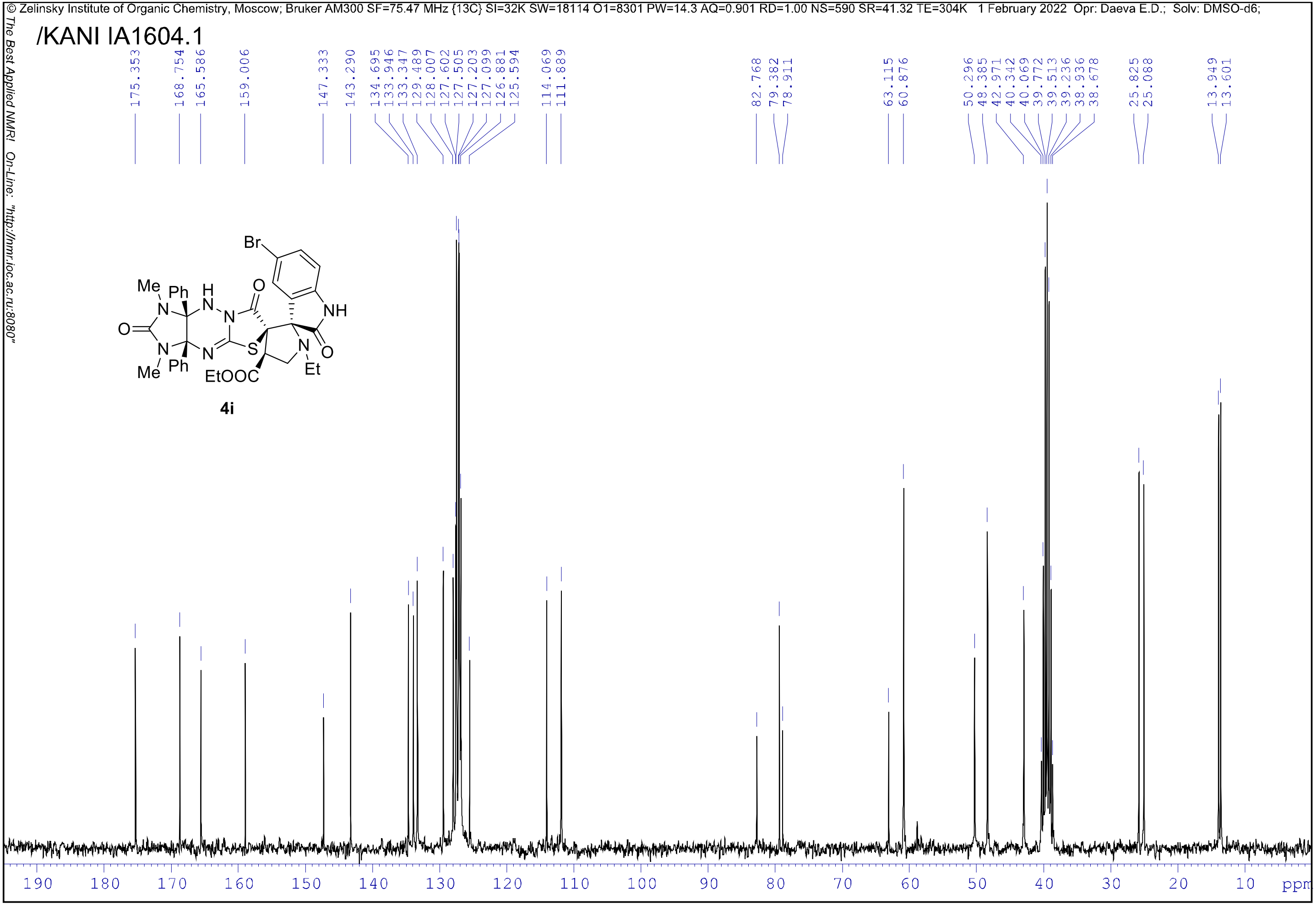
© Zelinsky Institute of Organic Chemistry, Moscow; Bruker AM300 SF=75.47 MHz {13C} SI=32K SW=18114 O1=8301 PW=14.3 AQ=0.901 RD=1.00 NS=590 SR=41.32 TE=304K 1 February 2022 Opr: Daeva E.D.; Solv: DMSO-d6;
The Best Applied NMR! On-Line: "http://nmr.ioc.ac.ru:8080"
/KANI IA1604.1
175.353
168.754
165.586
159.006
147.333
143.290
134.695
133.946
133.347
129.489
128.007
127.602
127.505
127.203
127.099
126.881
125.594
114.069
111.889
82.768
79.382
78.911
63.115
60.876
50.296
48.385
42.971
40.342
40.069
39.772
39.513
39.236
38.936
38.678
25.825
25.088
13.949
13.601
Br
Me
Ph
H
N
O
NH
EtOOC
S
Et
4i
190 180 170 160 150 140 130 120 110 100 90 80 70 60 50 40 30 20 10 ppm

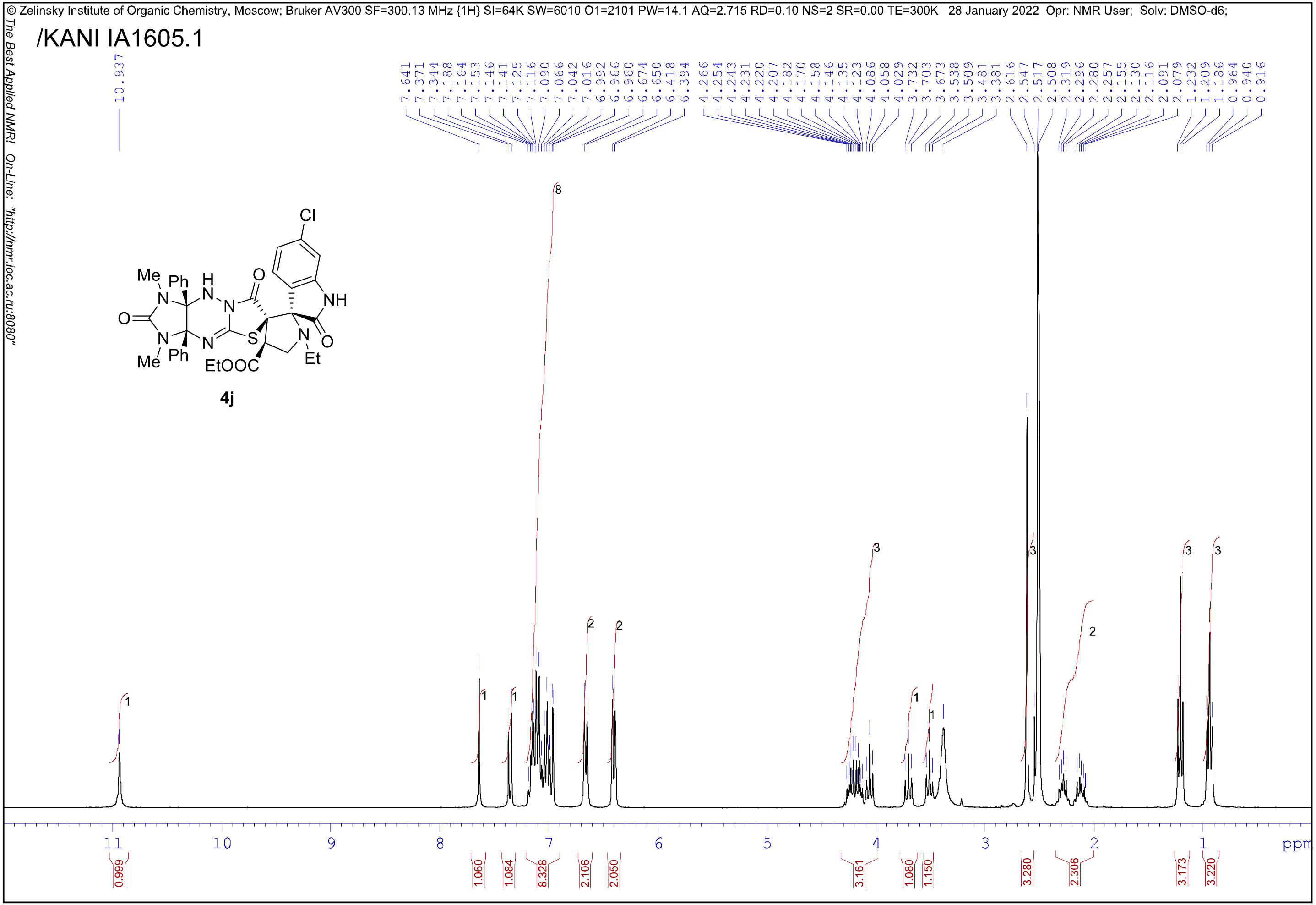
© Zelinsky Institute of Organic Chemistry, Moscow; Bruker AV300 SF=300.13 MHz {1H} SI=64K SW=6010 O1=2101 PW=14.1 AQ=2.715 RD=0.10 NS=2 SR=0.00 TE=300K 28 January 2022 Opr: NMR User; Solv: DMSO-d6;
The Best Applied NMR! On-Line: "http://nmr.ioc.ac.ru:8080"
/KANI IA1605.1
10.937
7.641 7.371 7.344 7.188 7.164 7.153 7.146 7.141 7.125 7.116 7.090 7.066 7.042 7.016 6.992 6.966 6.960 6.674 6.650 6.418 6.394
4.266 4.254 4.243 4.231 4.220 4.207 4.182 4.170 4.158 4.146 4.135 4.123 4.086 4.058 4.029 3.732 3.703 3.673 3.538 3.509 3.481 3.381
2.616 2.547 2.517 2.508 2.319 2.296 2.280 2.257 2.155 2.130 2.116 2.091 2.079 1.232 1.209 1.186 0.964 0.940 0.916
4j
ppm
0.999 1.060 1.084 8.328 2.106 2.050 3.161 1.080 1.150 3.280 2.306 3.173 3.220

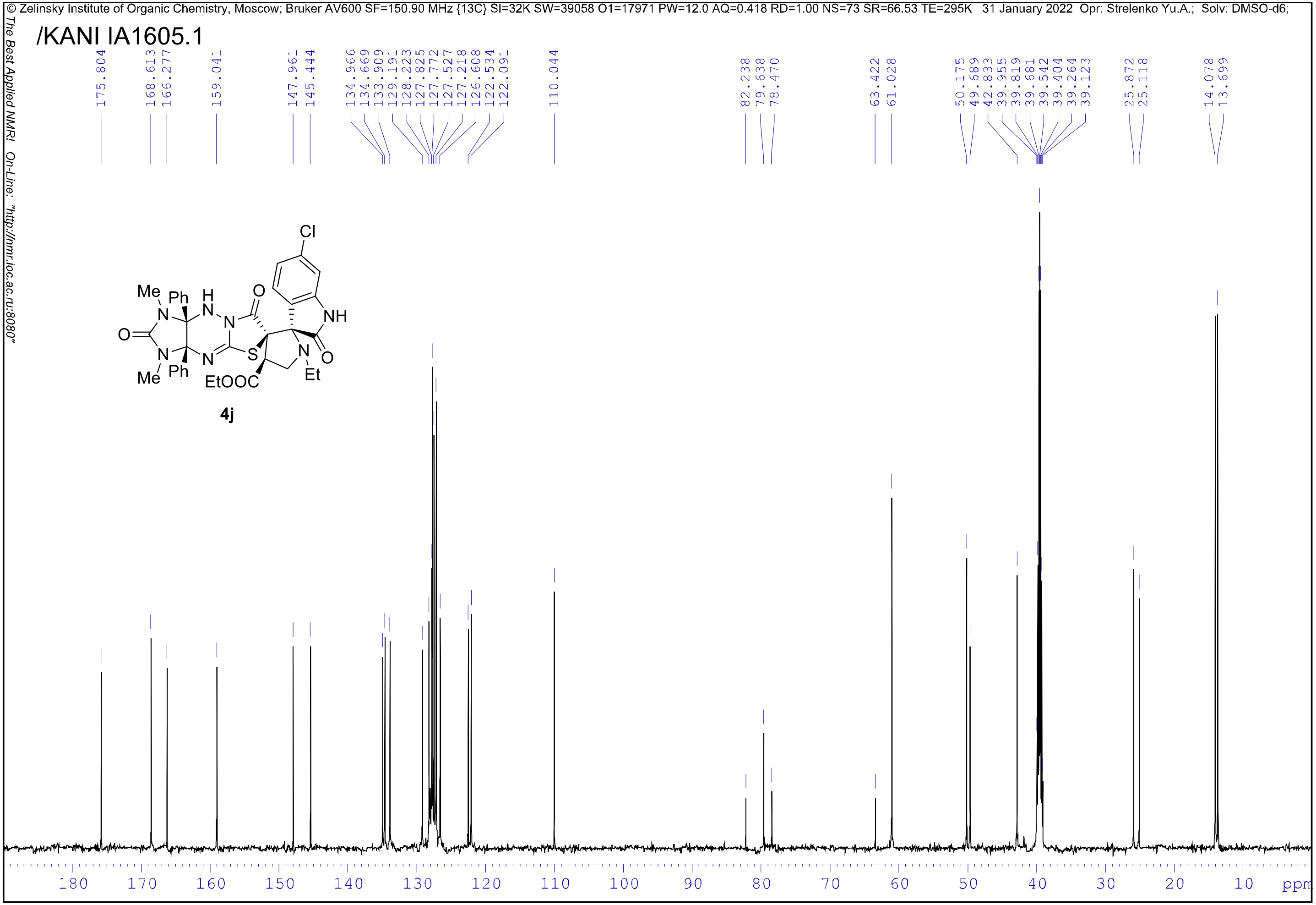
© Zelinsky Institute of Organic Chemistry, Moscow; Bruker AV600 SF=150.90 MHz {13C} SI=32K SW=39058 O1=17971 PW=12.0 AQ=0.418 RD=1.00 NS=73 SR=66.53 TE=295K 31 January 2022 Opr: Strelenko Yu.A.; Solv: DMSO-d6;
The Best Applied NMR! On-Line: "http://nmr.ioc.ac.ru:8080"
/KANI IA1605.1
175.804
168.613
166.277
159.041
147.961
145.444
134.966
134.669
133.909
129.191
128.223
127.825
127.772
127.527
127.218
126.608
122.534
122.091
110.044
82.238
79.638
78.470
63.422
61.028
50.175
49.689
42.833
39.955
39.819
39.681
39.542
39.404
39.264
39.123
25.872
25.118
14.078
13.699
Cl
Me
Ph
H
N
O
NH
EtOOC
S
Et
4j
180 170 160 150 140 130 120 110 100 90 80 70 60 50 40 30 20 10 ppm

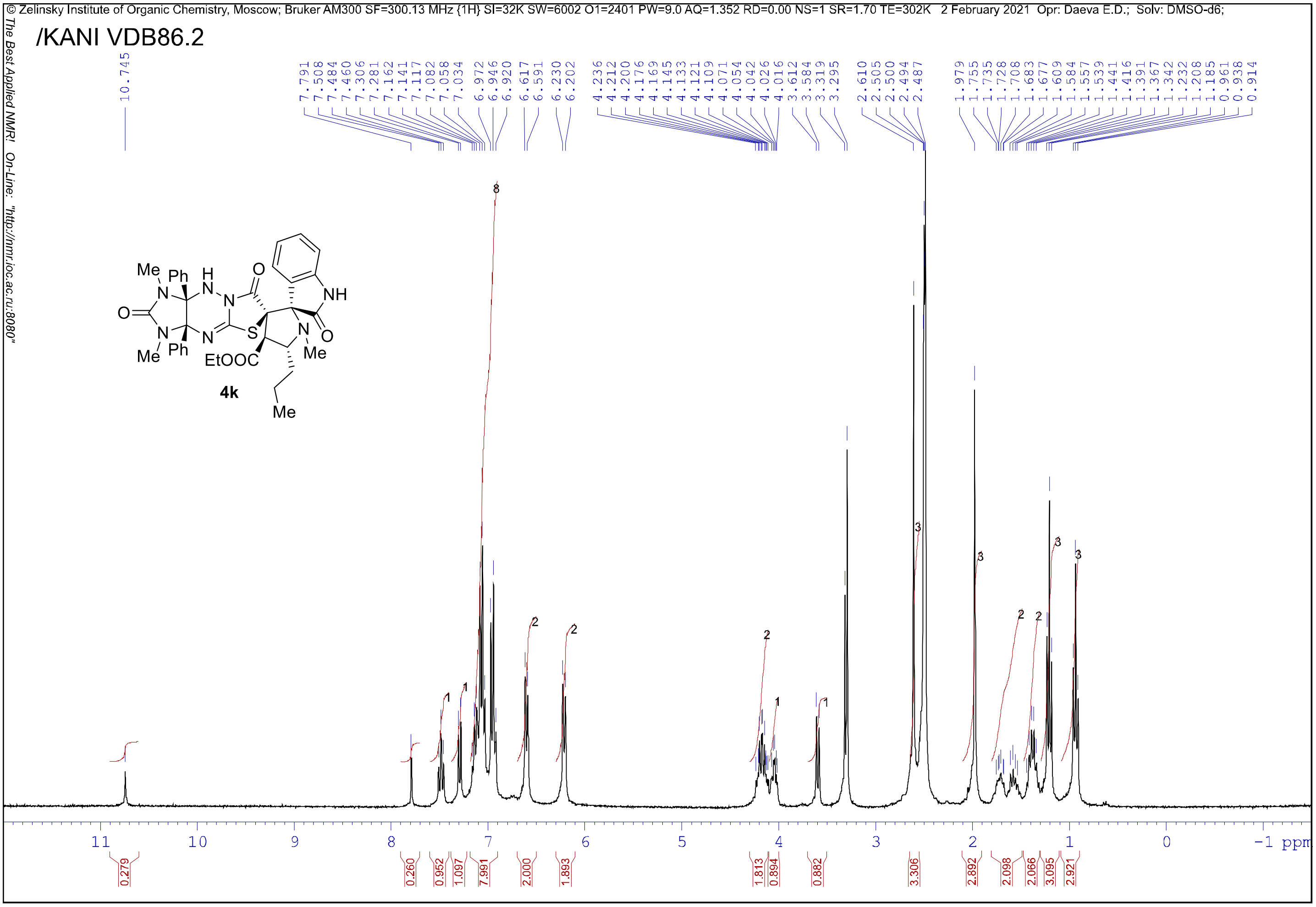
© Zelinsky Institute of Organic Chemistry, Moscow; Bruker AM300 SF=300.13 MHz {1H} SI=32K SW=6002 O1=2401 PW=9.0 AQ=1.352 RD=0.00 NS=1 SR=1.70 TE=302K 2 February 2021 Opr: Daeva E.D.; Solv: DMSO-d6;
The Best Applied NMR! On-Line: "http://nmr.ioc.ac.ru:8080"
/KANI VDB86.2
10.745
7.791 7.508 7.484 7.460 7.306 7.281 7.162 7.141 7.117 7.082 7.058 7.034 6.972 6.946 6.920 6.617 6.591 6.230 6.202
4.236 4.212 4.200 4.176 4.169 4.145 4.133 4.121 4.109 4.071 4.054 4.042 4.026 4.016 3.612 3.584 3.319 3.295
2.610 2.505 2.500 2.494 2.487
1.979 1.755 1.735 1.728 1.708 1.683 1.677 1.609 1.584 1.557 1.539 1.441 1.416 1.391 1.367 1.342 1.232 1.208 1.185 0.961 0.938 0.914
Me Ph H N N N O S NH O N Me EtOOC Me Ph O N N Me
4k
11 10 9 8 7 6 5 4 3 2 1 0 -1 ppm
0.279 0.260 0.952 1.097 7.991 2.000 1.893 1.813 0.894 0.882 3.306 2.892 2.098 2.066 3.095 2.921

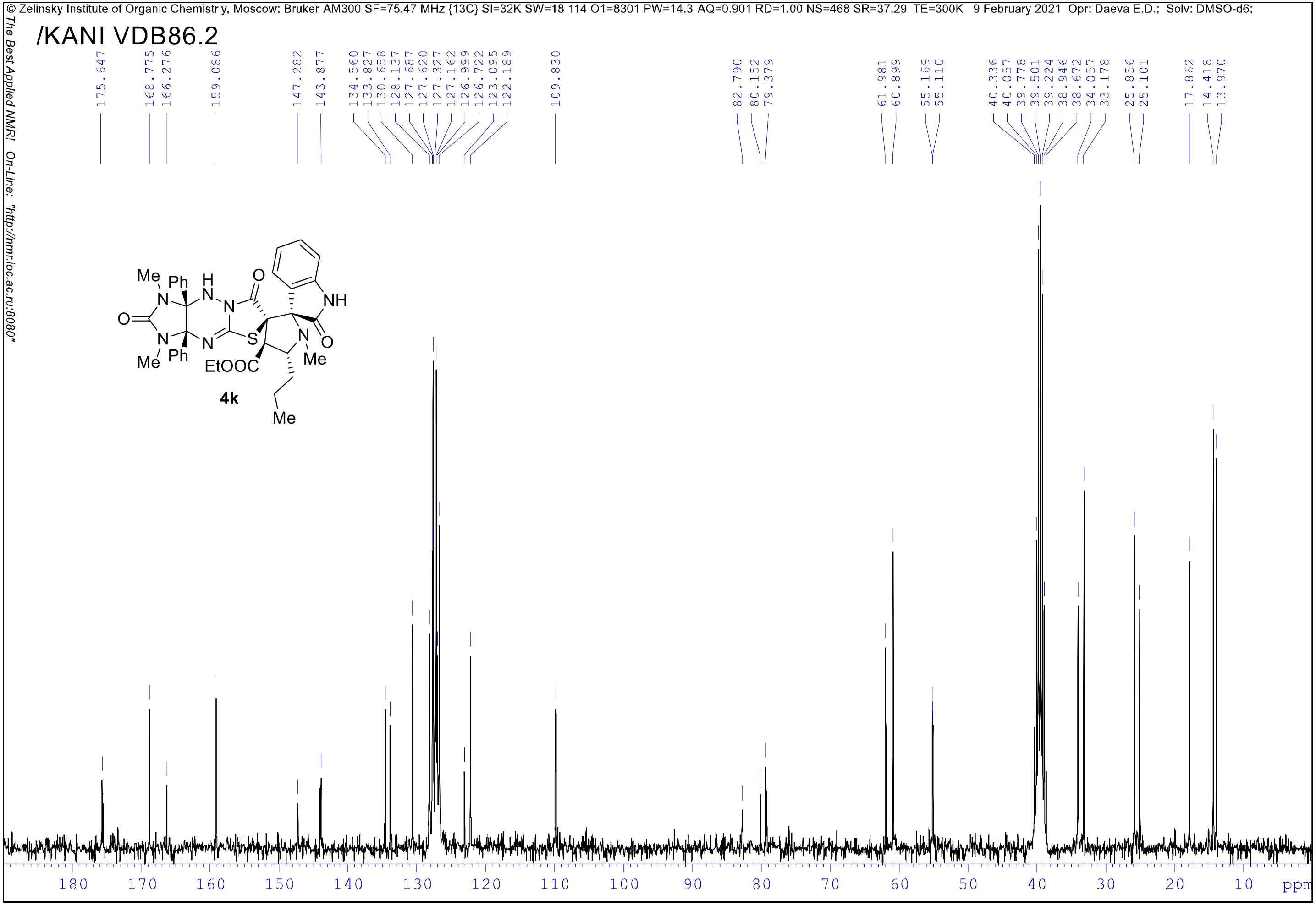
© Zelinsky Institute of Organic Chemistr y, Moscow; Bruker AM300 SF=75.47 MHz {13C} SI=32K SW=18 114 O1=8301 PW=14.3 AQ=0.901 RD=1.00 NS=468 SR=37.29 TE=300K 9 February 2021 Opr: Daeva E.D.; Solv: DMSO-d6;
The Best Applied NMR! On-Line: "http://nmr.ioc.ac.ru:8080"
/KANI VDB86.2
175.647
168.775
166.276
159.086
147.282
143.877
134.560
133.827
130.658
128.137
127.687
127.620
127.327
127.162
126.999
126.722
123.095
122.189
109.830
82.790
80.152
79.379
61.981
60.899
55.169
55.110
40.336
40.057
39.778
39.501
39.224
38.946
38.672
34.057
33.178
25.856
25.101
17.862
14.418
13.970
Me
Ph
H
N
O
NH
EtOOC
S
Me
4k
180 170 160 150 140 130 120 110 100 90 80 70 60 50 40 30 20 10 ppm

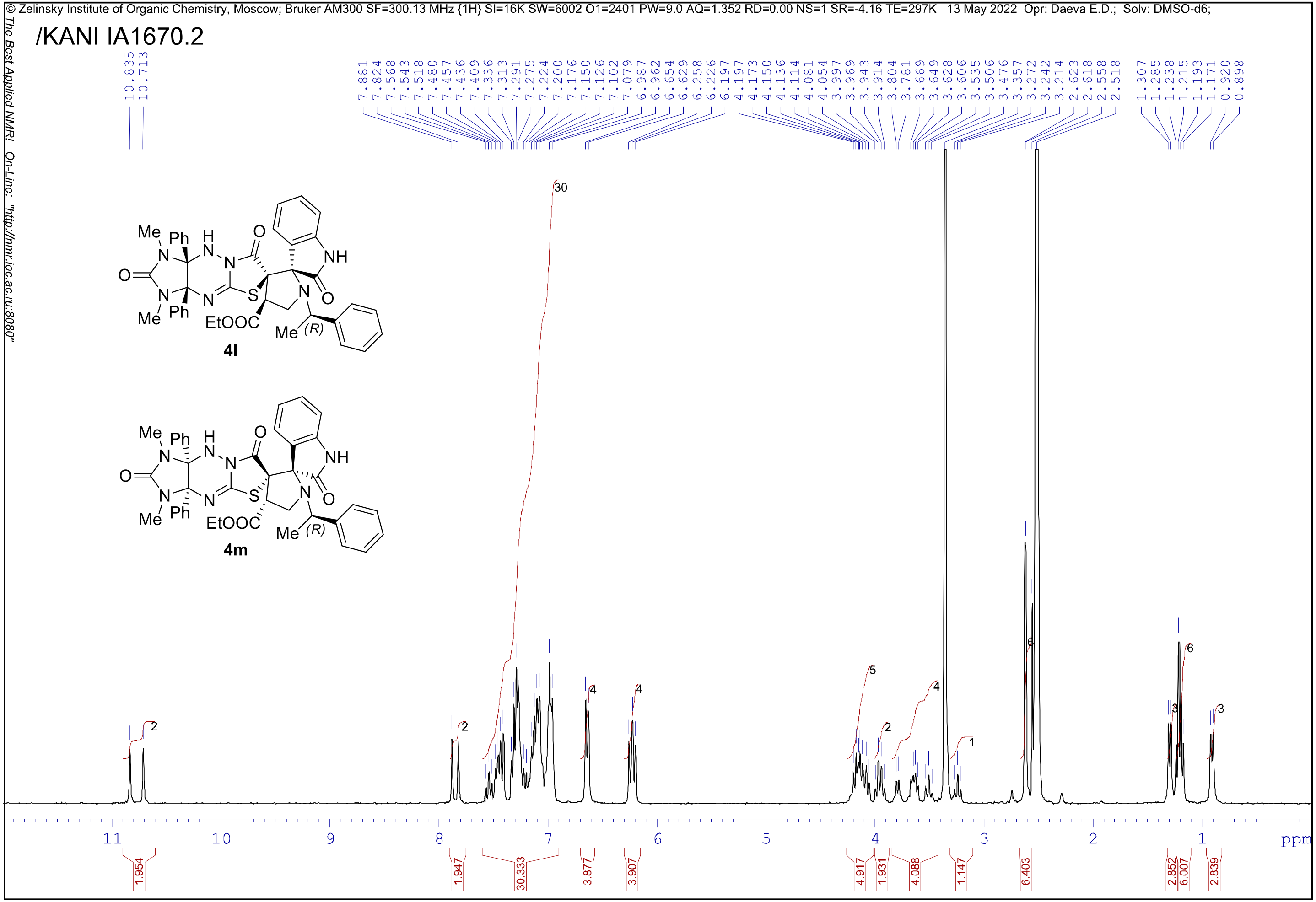
© Zelinsky Institute of Organic Chemistry, Moscow; Bruker AM300 SF=300.13 MHz {1H} SI=16K SW=6002 O1=2401 PW=9.0 AQ=1.352 RD=0.00 NS=1 SR=-4.16 TE=297K 13 May 2022 Opr: Daeva E.D.; Solv: DMSO-d6;
/KANI IA1670.2
The Best Applied NMR! On-Line: "http://nmr.ioc.ac.ru:8080"
10.835 10.713
7.881 7.824 7.568 7.543 7.518 7.480 7.457 7.436 7.409 7.336 7.313 7.291 7.275 7.224 7.200 7.176 7.150 7.126 7.102 7.079 6.987 6.962 6.654 6.629 6.258 6.226 6.197
4.197 4.173 4.150 4.136 4.114 4.081 4.054 3.997 3.969 3.943 3.914 3.804 3.781 3.669 3.649 3.628 3.606 3.535 3.506 3.476 3.357 3.272 3.242 3.214 2.623 2.618 2.558 2.518
1.307 1.285 1.238 1.215 1.193 1.171 0.920 0.898
Me Ph H N N O N NH O N S N O N Ph Me EtOOC Me (R)
4l
4m
2 2 30 4 4 5 2 4 1 6 3 6 3
11 10 9 8 7 6 5 4 3 2 1 ppm
1.954 1.947 30.333 3.877 3.907 4.917 1.931 4.088 1.147 6.403 2.852 6.007 2.839

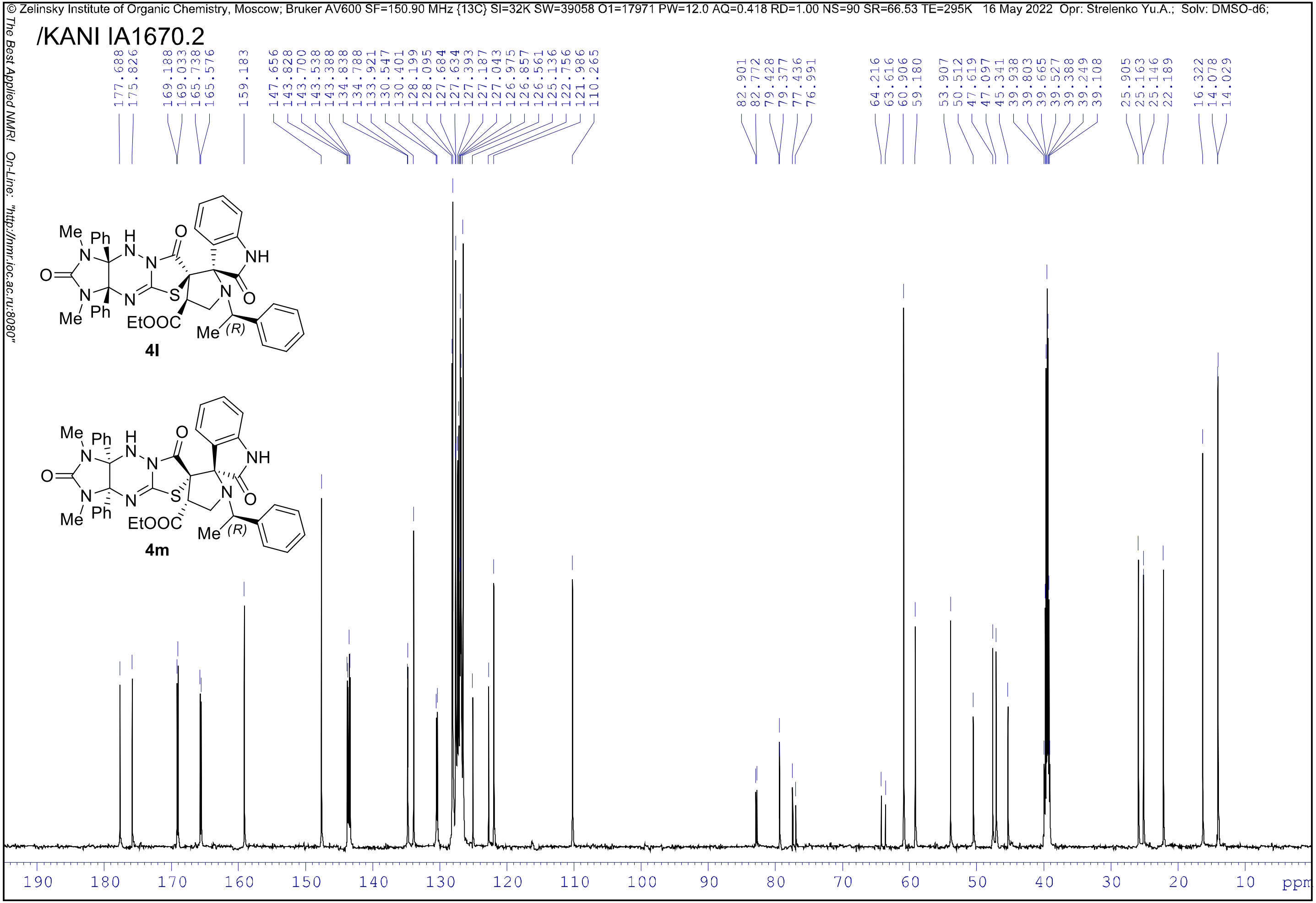
© Zelinsky Institute of Organic Chemistry, Moscow; Bruker AV600 SF=150.90 MHz {13C} SI=32K SW=39058 O1=17971 PW=12.0 AQ=0.418 RD=1.00 NS=90 SR=66.53 TE=295K 16 May 2022 Opr: Strelenko Yu.A.; Solv: DMSO-d6;
The Best Applied NMR! On-Line: "http://nmr.ioc.ac.ru:8080"
/KANI IA1670.2
177.688
175.826
169.188
169.033
165.738
165.576
159.183
147.656
143.828
143.700
143.538
143.388
134.838
134.788
133.921
130.547
130.401
128.199
128.095
127.684
127.634
127.393
127.187
127.043
126.975
126.857
126.561
125.136
122.756
121.986
110.265
82.901
82.772
79.428
79.377
77.436
76.991
64.216
63.616
60.906
59.180
53.907
50.512
47.619
47.097
45.341
39.938
39.803
39.665
39.527
39.388
39.249
39.108
25.905
25.163
25.146
22.189
16.322
14.078
14.029
4l
4m
190
180
170
160
150
140
130
120
110
100
90
80
70
60
50
40
30
20
10
ppm

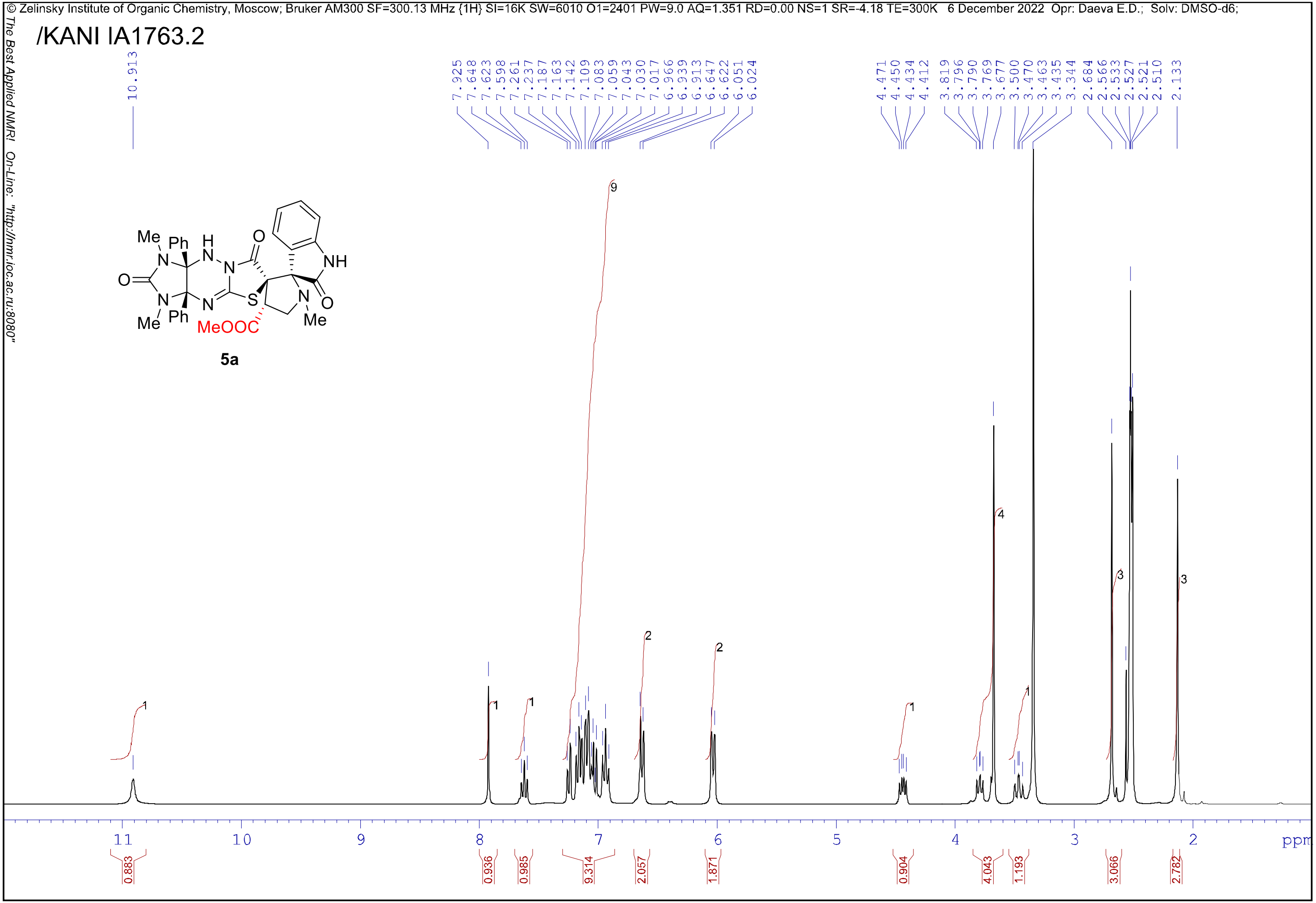

© Zelinsky Institute of Organic Chemistry, Moscow; Bruker AM300 SF=300.13 MHz {1H} SI=16K SW=6010 O1=2401 PW=9.0 AQ=1.351 RD=0.00 NS=1 SR=-4.18 TE=300K 6 December 2022 Opr: Daeva E.D.; Solv: DMSO-d6;
/KANI IA1763.2
The Best Applied NMR! On-Line: "http://nmr.ioc.ac.ru:8080"
10.913
7.925 7.648 7.623 7.598 7.261 7.237 7.187 7.163 7.142 7.109 7.083 7.059 7.043 7.030 7.017 6.966 6.939 6.913 6.647 6.622 6.051 6.024
4.471 4.450 4.434 4.412
3.819 3.796 3.790 3.769 3.677 3.500 3.470 3.463 3.435 3.344
2.684 2.566 2.533 2.527 2.521 2.510
2.133
Me
Ph
H
N
O
NH
S
MeOOC
5a
0.883 0.936 0.985 9.314 2.057 1.871 0.904 4.043 1.193 3.066 2.782
11 10 9 8 7 6 5 4 3 2 ppm

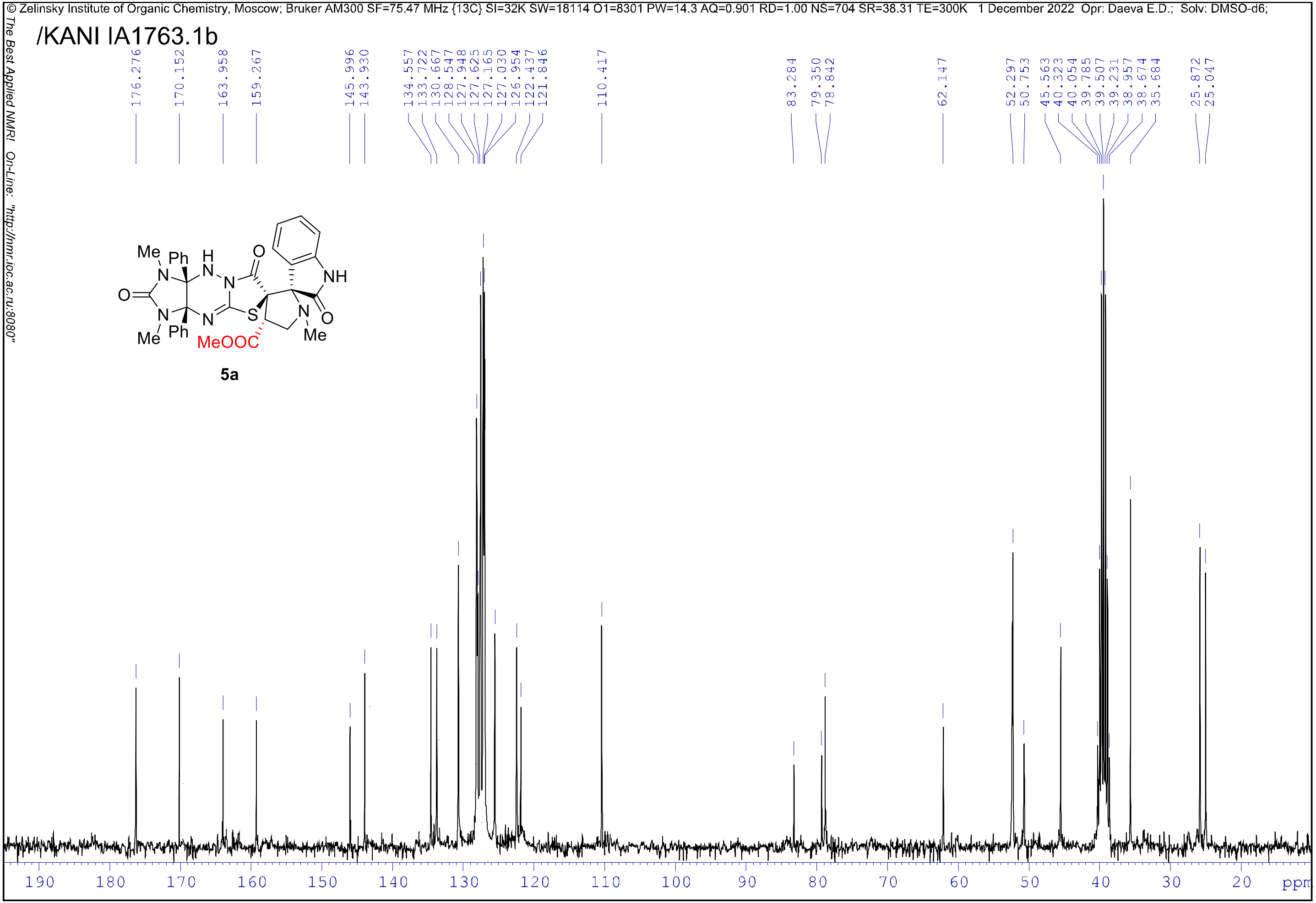

© Zelinsky Institute of Organic Chemistry, Moscow; Bruker AM300 SF=75.47 MHz {13C} SI=32K SW=18114 O1=8301 PW=14.3 AQ=0.901 RD=1.00 NS=704 SR=38.31 TE=300K 1 December 2022 Opr: Daeva E.D.; Solv: DMSO-d6;
The Best Applied NMR! On-Line: "http://nmr.ioc.ac.ru:8080"
/KANI IA1763.1b
176.276
170.152
163.958
159.267
145.996
143.930
134.557
133.722
130.667
128.547
127.948
127.625
127.165
127.030
126.954
122.437
121.846
110.417
83.284
79.350
78.842
62.147
52.297
50.753
45.563
40.323
40.054
39.785
39.507
39.231
38.957
38.674
35.684
25.872
25.047
Me
Ph
H
N
O
NH
O
S
Ph
MeOOC
Me
5a
190 180 170 160 150 140 130 120 110 100 90 80 70 60 50 40 30 20 ppm

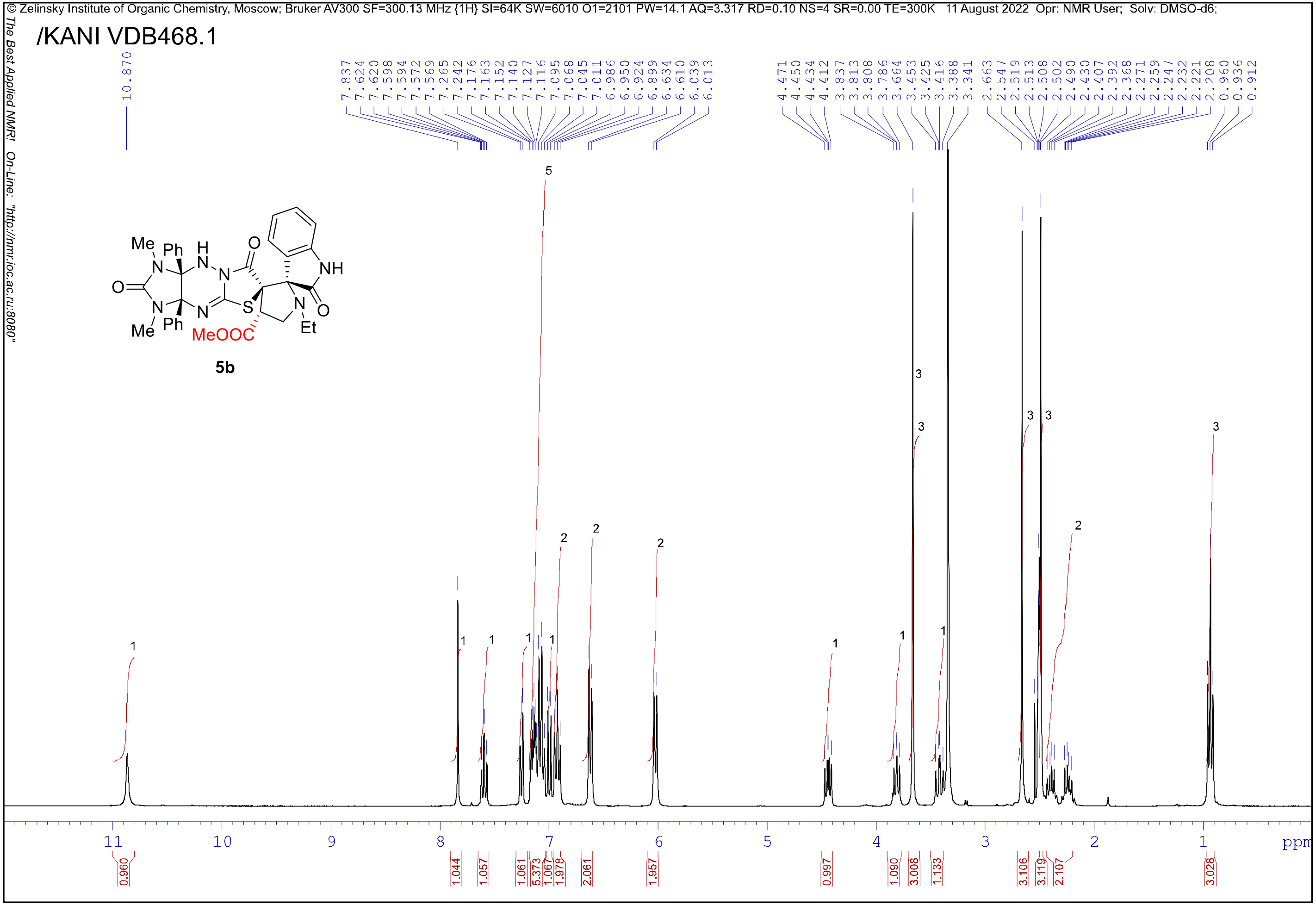
© Zelinsky Institute of Organic Chemistry, Moscow; Bruker AV300 SF=300.13 MHz {1H} SI=64K SW=6010 O1=2101 PW=14.1 AQ=3.317 RD=0.10 NS=4 SR=0.00 TE=300K 11 August 2022 Opr: NMR User; Solv: DMSO-d6;
The Best Applied NMR! On-Line: "http://nmr.ioc.ac.ru:8080"
/KANI VDB468.1
10.870
7.837 7.624 7.620 7.598 7.594 7.572 7.569 7.265 7.242 7.176 7.163 7.152 7.140 7.127 7.116 7.095 7.068 7.045 7.011 6.986 6.950 6.924 6.899 6.634 6.610 6.039 6.013
4.471 4.450 4.434 4.412 3.837 3.813 3.808 3.786 3.664 3.453 3.425 3.416 3.388 3.341
2.663 2.547 2.519 2.513 2.508 2.502 2.490 2.430 2.407 2.392 2.368 2.271 2.259 2.247 2.232 2.221 2.208 0.960 0.936 0.912
Me
Ph
H
N
O
NH
O
Ph
Me
MeOOC
S
Et
5b
ppm
11 10 9 8 7 6 5 4 3 2 1
0.960 1.044 1.057 1.061 5.373 1.067 1.978 2.061 1.957 0.997 1.090 3.008 1.133 3.106 3.119 2.107 3.028

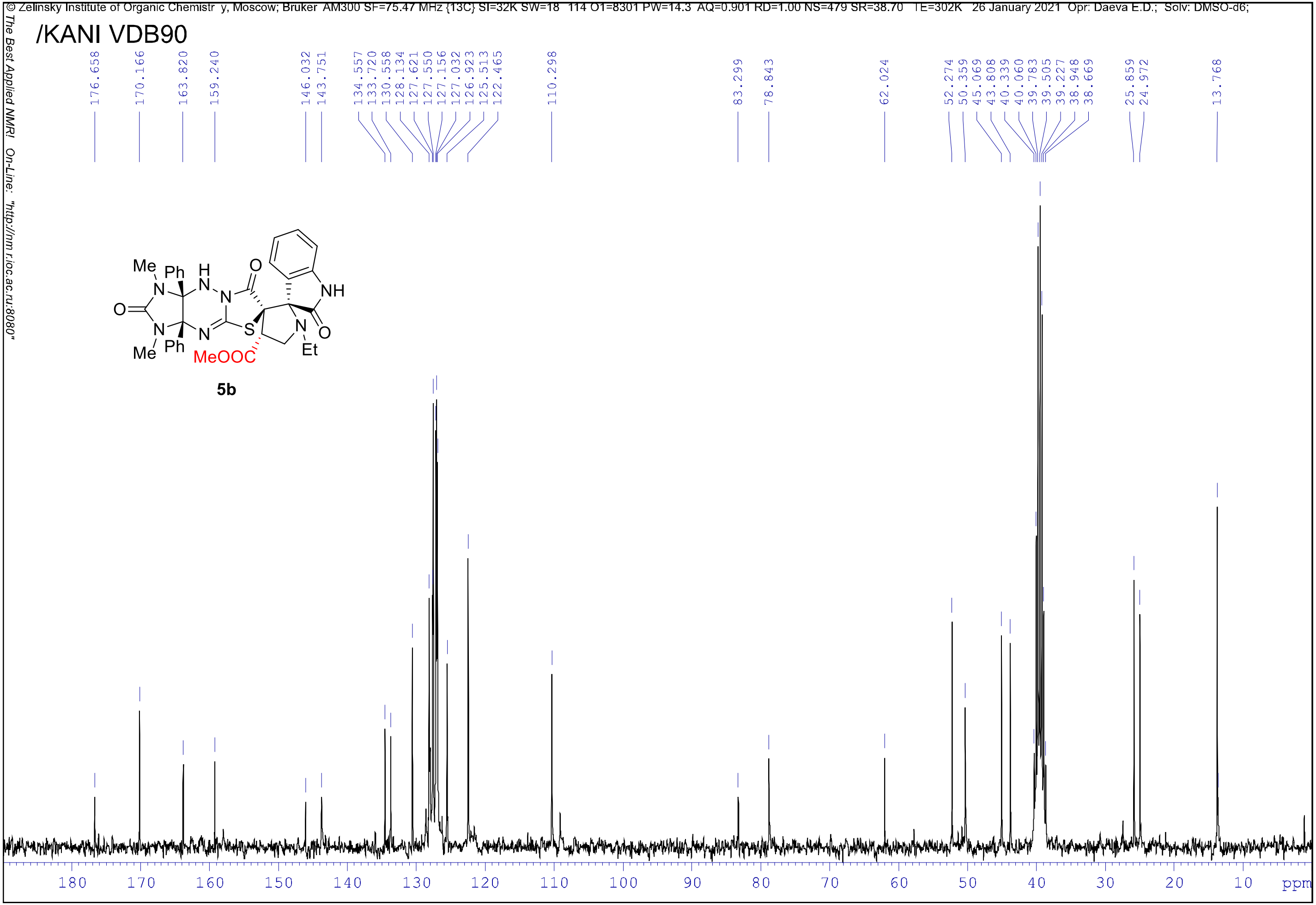
© Zelinsky Institute of Organic Chemistr y, Moscow; Bruker AM300 SF=75.47 MHz {13C} SI=32K SW=18 114 O1=8301 PW=14.3 AQ=0.901 RD=1.00 NS=479 SR=38.70 TE=302K 26 January 2021 Opr: Daeva E.D.; Solv: DMSO-d6;
The Best Applied NMR! On-Line: "http://nm r.ioc.ac.ru:8080"
/KANI VDB90
176.658
170.166
163.820
159.240
146.032
143.751
134.557
133.720
130.558
128.134
127.621
127.550
127.156
127.032
126.923
125.513
122.465
110.298
83.299
78.843
62.024
52.274
50.359
45.069
43.808
40.339
40.060
39.783
39.505
39.227
38.948
38.669
25.859
24.972
13.768
Me
Ph
H
N
O
N
N
NH
O
N
S
N
O
N
Me
Ph
MeOOC
Et
5b
180
170
160
150
140
130
120
110
100
90
80
70
60
50
40
30
20
10
ppm

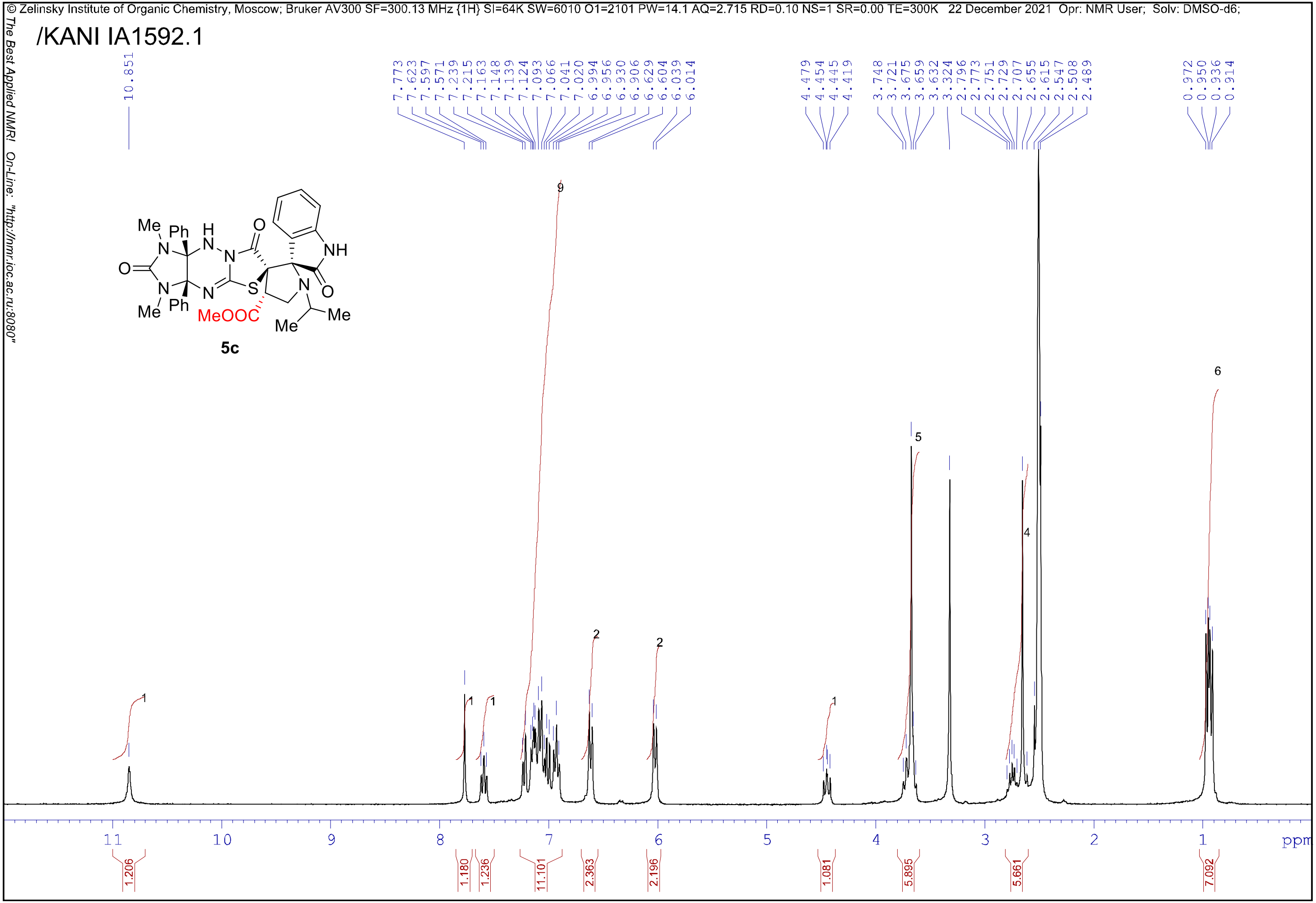
© Zelinsky Institute of Organic Chemistry, Moscow; Bruker AV300 SF=300.13 MHz {1H} SI=64K SW=6010 O1=2101 PW=14.1 AQ=2.715 RD=0.10 NS=1 SR=0.00 TE=300K 22 December 2021 Opr: NMR User; Solv: DMSO-d6;
The Best Applied NMR! On-Line: "http://nmr.ioc.ac.ru:8080"
/KANI IA1592.1
10.851
7.773 7.623 7.597 7.571 7.239 7.215 7.163 7.148 7.139 7.124 7.093 7.066 7.041 7.020 6.994 6.956 6.930 6.906 6.629 6.604 6.039 6.014
4.479 4.454 4.445 4.419
3.748 3.721 3.675 3.659 3.632 3.324 2.796 2.773 2.751 2.729 2.707 2.655 2.615 2.547 2.508 2.489
0.972 0.950 0.936 0.914
Me Ph H N N N O O N S N NH O N Me Ph MeOOC Me Me
5c
1 1 1 9 2 2 1 5 4 6
11 10 9 8 7 6 5 4 3 2 1 ppm
1.206 1.180 1.236 11.101 2.363 2.196 1.081 5.895 5.661 7.092

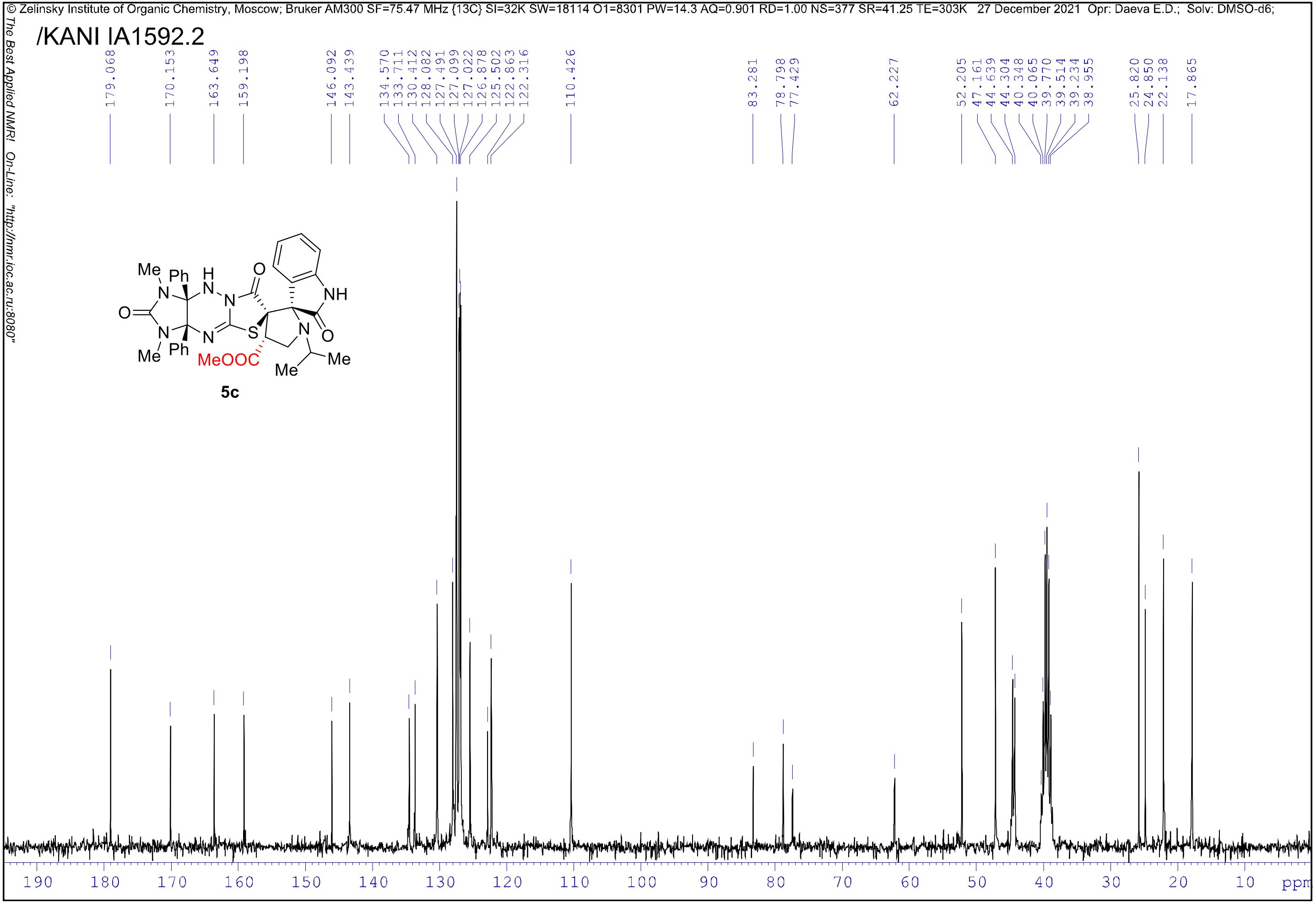
© Zelinsky Institute of Organic Chemistry, Moscow; Bruker AM300 SF=75.47 MHz {13C} SI=32K SW=18114 O1=8301 PW=14.3 AQ=0.901 RD=1.00 NS=377 SR=41.25 TE=303K 27 December 2021 Opr: Daeva E.D.; Solv: DMSO-d6;
The Best Applied NMR! On-Line: "http://nmr.ioc.ac.ru:8080"
/KANI IA1592.2
179.068
170.153
163.649
159.198
146.092
143.439
134.570
133.711
130.412
128.082
127.491
127.099
127.022
126.878
125.502
122.863
122.316
110.426
83.281
78.798
77.429
62.227
52.205
47.161
44.639
44.304
40.348
40.065
39.770
39.514
39.234
38.955
25.820
24.850
22.138
17.865
Me
Ph
H
N
O
N
N
NH
O
N
N
S
N
O
Me
Ph
MeOOC
Me
Me
5c
190
180
170
160
150
140
130
120
110
100
90
80
70
60
50
40
30
20
10
ppm

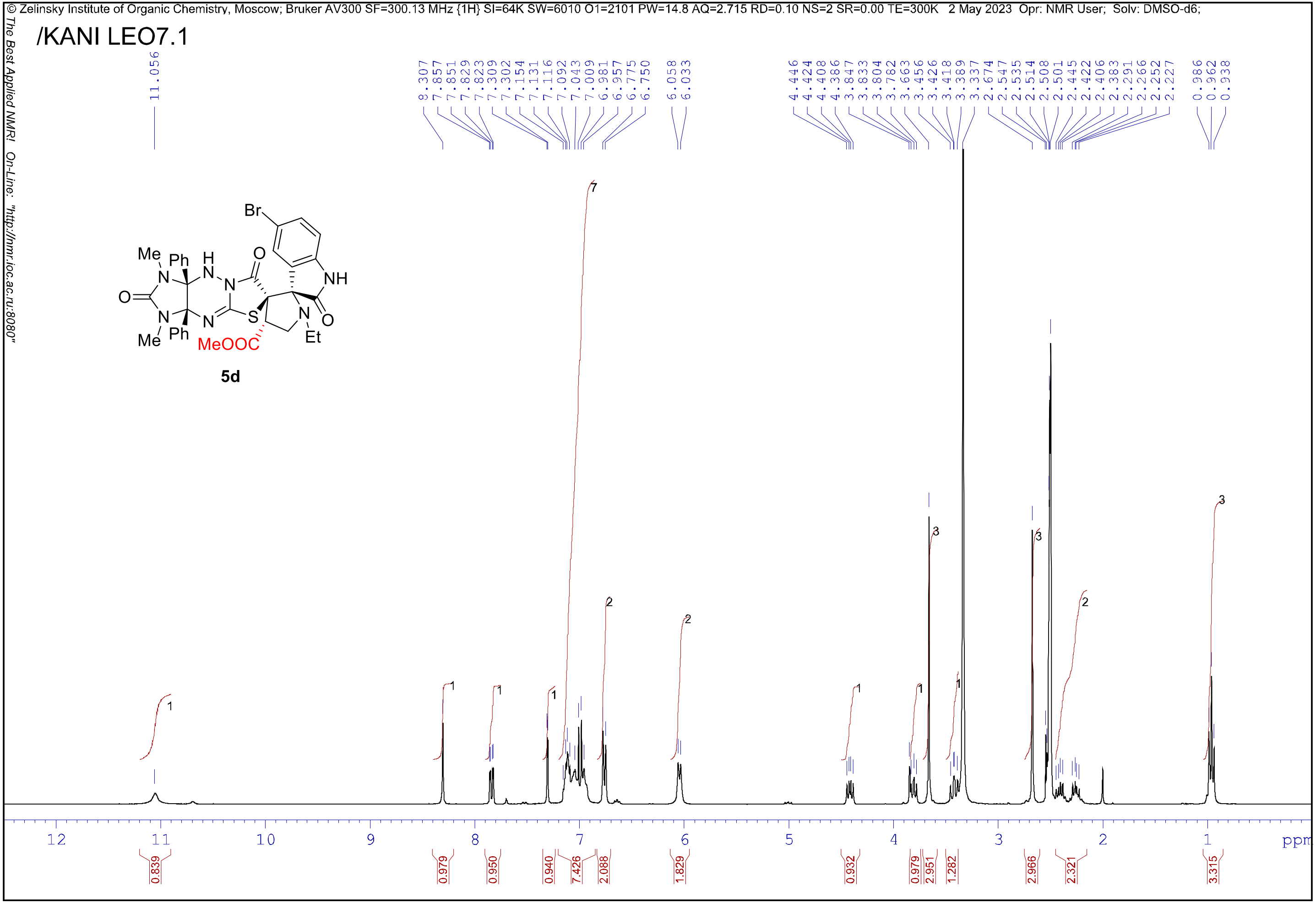
© Zelinsky Institute of Organic Chemistry, Moscow; Bruker AV300 SF=300.13 MHz {1H} SI=64K SW=6010 O1=2101 PW=14.8 AQ=2.715 RD=0.10 NS=2 SR=0.00 TE=300K 2 May 2023 Opr: NMR User; Solv: DMSO-d6;
The Best Applied NMR! On-Line: "http://nmr.ioc.ac.ru:8080"
/KANI LEO7.1
11.056
8.307 7.857 7.851 7.829 7.823 7.309 7.302 7.154 7.131 7.116 7.092 7.043 7.009 6.981 6.957 6.775 6.750
6.058 6.033
4.446 4.424 4.408 4.386 3.847 3.833 3.804 3.782 3.663 3.456 3.426 3.418 3.389 3.337 2.674 2.547 2.535 2.514 2.508 2.501 2.445 2.422 2.406 2.383 2.291 2.266 2.252 2.227
0.986 0.962 0.938
Br
Me
Ph
H
N
O
NH
Et
MeOOC
S
5d
12 11 10 9 8 7 6 5 4 3 2 1 ppm
0.839 0.979 0.950 0.940 7.426 2.088 1.829 0.932 0.979 2.951 1.282 2.966 2.321 3.315

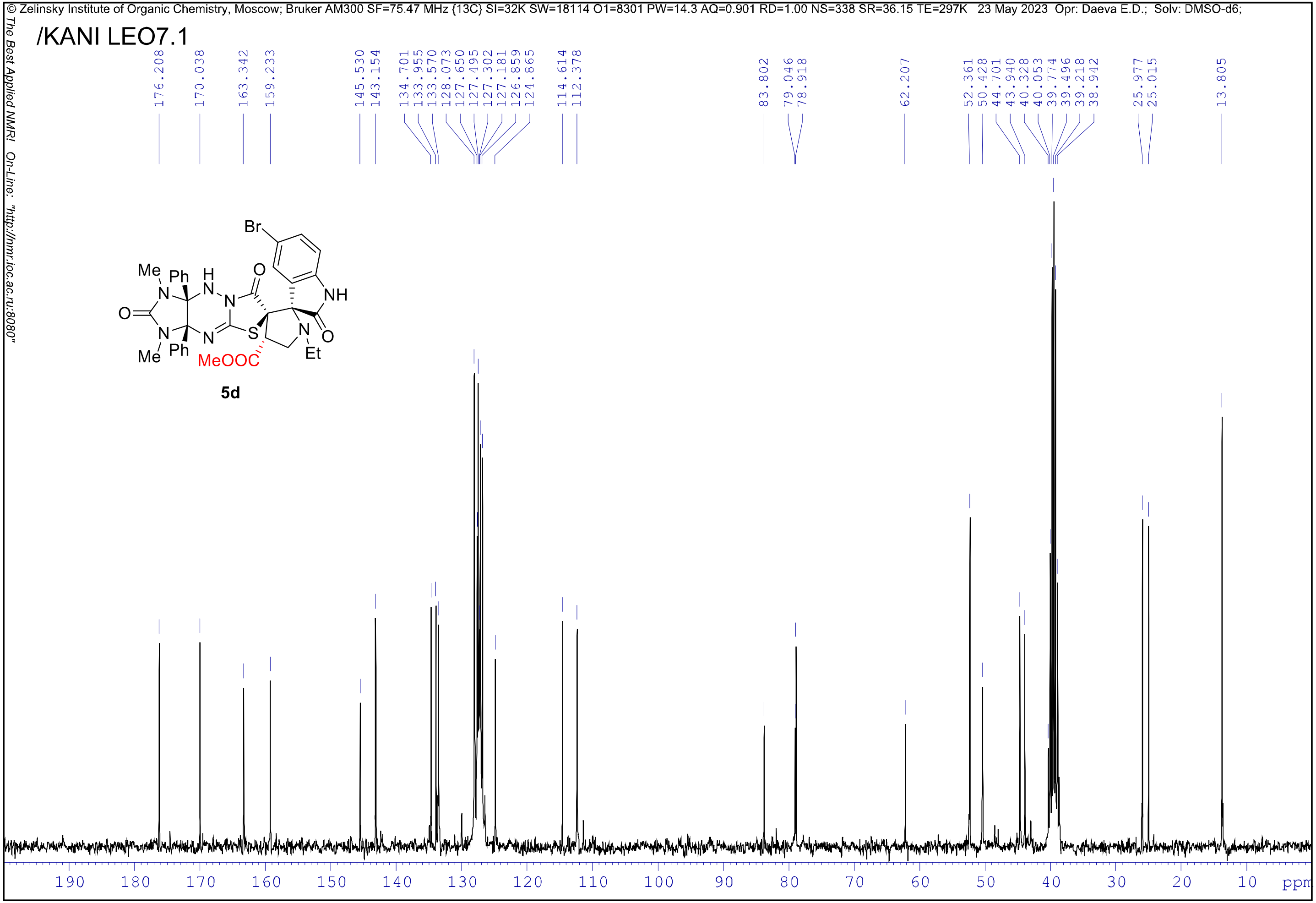
© Zelinsky Institute of Organic Chemistry, Moscow; Bruker AM300 SF=75.47 MHz {13C} SI=32K SW=18114 O1=8301 PW=14.3 AQ=0.901 RD=1.00 NS=338 SR=36.15 TE=297K 23 May 2023 Opr: Daeva E.D.; Solv: DMSO-d6;
The Best Applied NMR! On-Line: "http://nmr.ioc.ac.ru:8080"
/KANI LEO7.1
176.208
170.038
163.342
159.233
145.530
143.154
134.701
133.955
133.570
128.073
127.650
127.495
127.302
127.181
126.859
124.865
114.614
112.378
83.802
79.046
78.918
62.207
52.361
50.428
44.701
43.940
40.328
40.053
39.774
39.496
39.218
38.942
25.977
25.015
13.805
Br
Me
Ph
H
N
O
NH
S
Et
MeOOC
5d
190 180 170 160 150 140 130 120 110 100 90 80 70 60 50 40 30 20 10 ppm

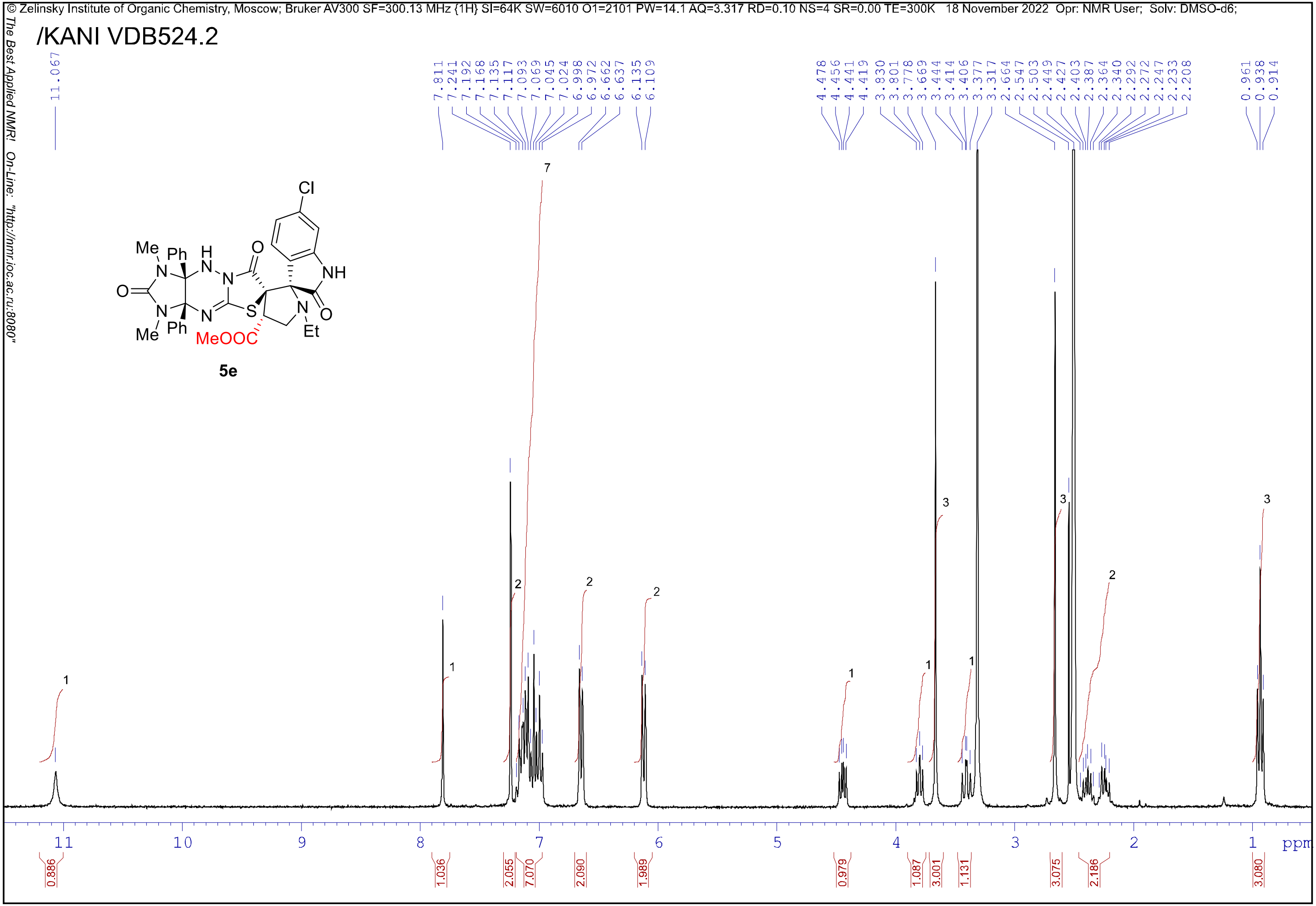
© Zelinsky Institute of Organic Chemistry, Moscow; Bruker AV300 SF=300.13 MHz {1H} SI=64K SW=6010 O1=2101 PW=14.1 AQ=3.317 RD=0.10 NS=4 SR=0.00 TE=300K 18 November 2022 Opr: NMR User; Solv: DMSO-d6;
/KANI VDB524.2
The Best Applied NMR! On-Line: "http://nmr.ioc.ac.ru:8080"
11.067
7.811 7.241 7.192 7.168 7.135 7.117 7.093 7.069 7.045 7.024 6.998 6.972 6.662 6.637 6.135 6.109
4.478 4.456 4.441 4.419 3.830 3.801 3.778 3.669 3.444 3.414 3.406 3.377 3.317 2.664 2.547 2.503 2.449 2.427 2.403 2.387 2.364 2.340 2.292 2.272 2.247 2.233 2.208
0.961 0.938 0.914
5e
ppm
0.886 1.036 2.055 7.070 2.090 1.989 0.979 1.087 3.001 1.131 3.075 2.186 3.080

© Zelinsky Institute of Organic Chemistry, Moscow; Bruker AV600 SF=150.90 MHz {13C} SI=32K SW=39058 O1=17971 PW=12.0 AQ=0.418 RD=1.00 NS=208 SR=66.53 TE=297K 21 November 2022 Opr: Strelenko Yu.A.; Solv: DMSO-d6;

The Best Applied NMR! On-Line: "http://nmr.ioc.ac.ru:8080"

/KANI VDB524.2

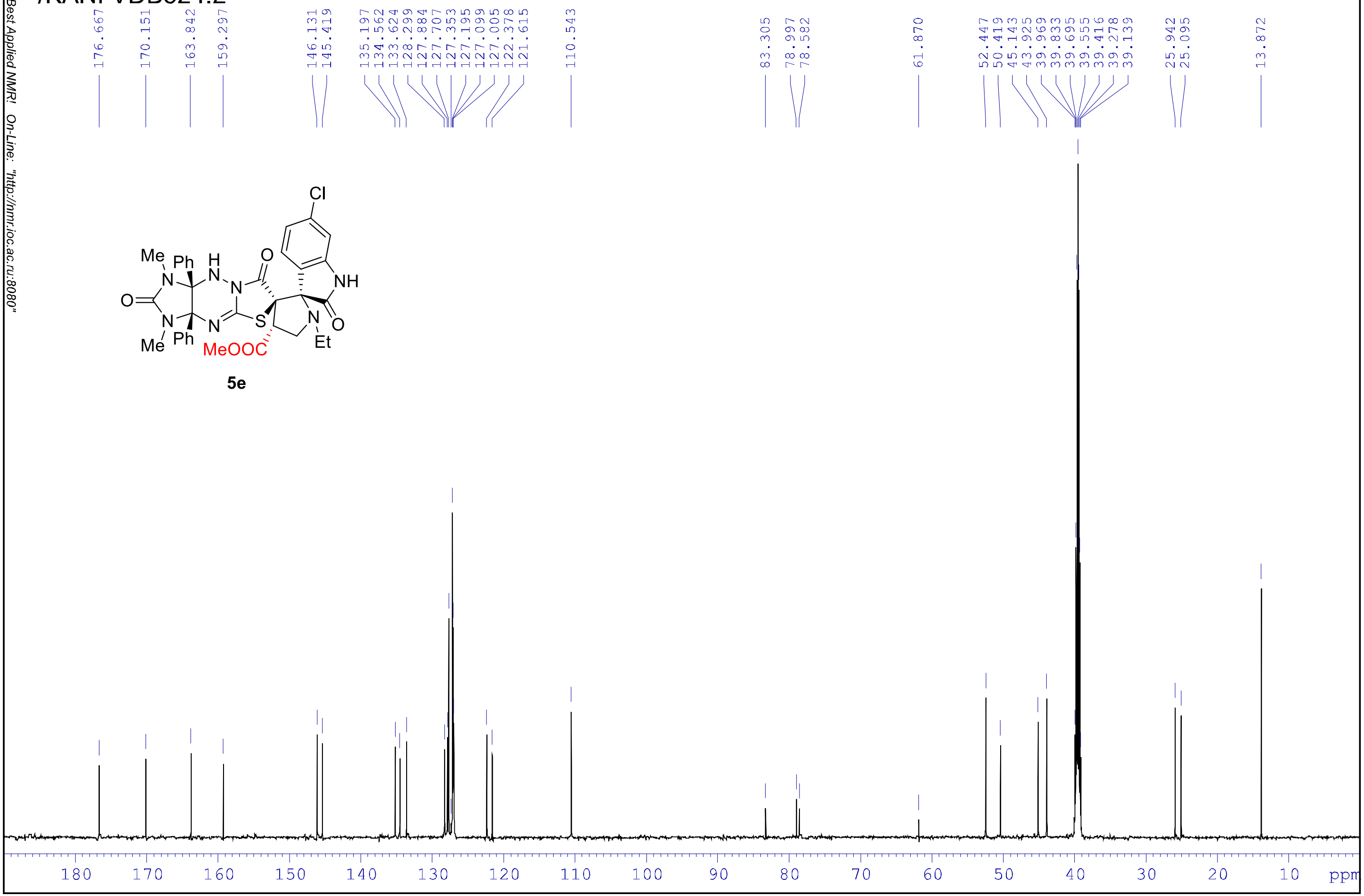

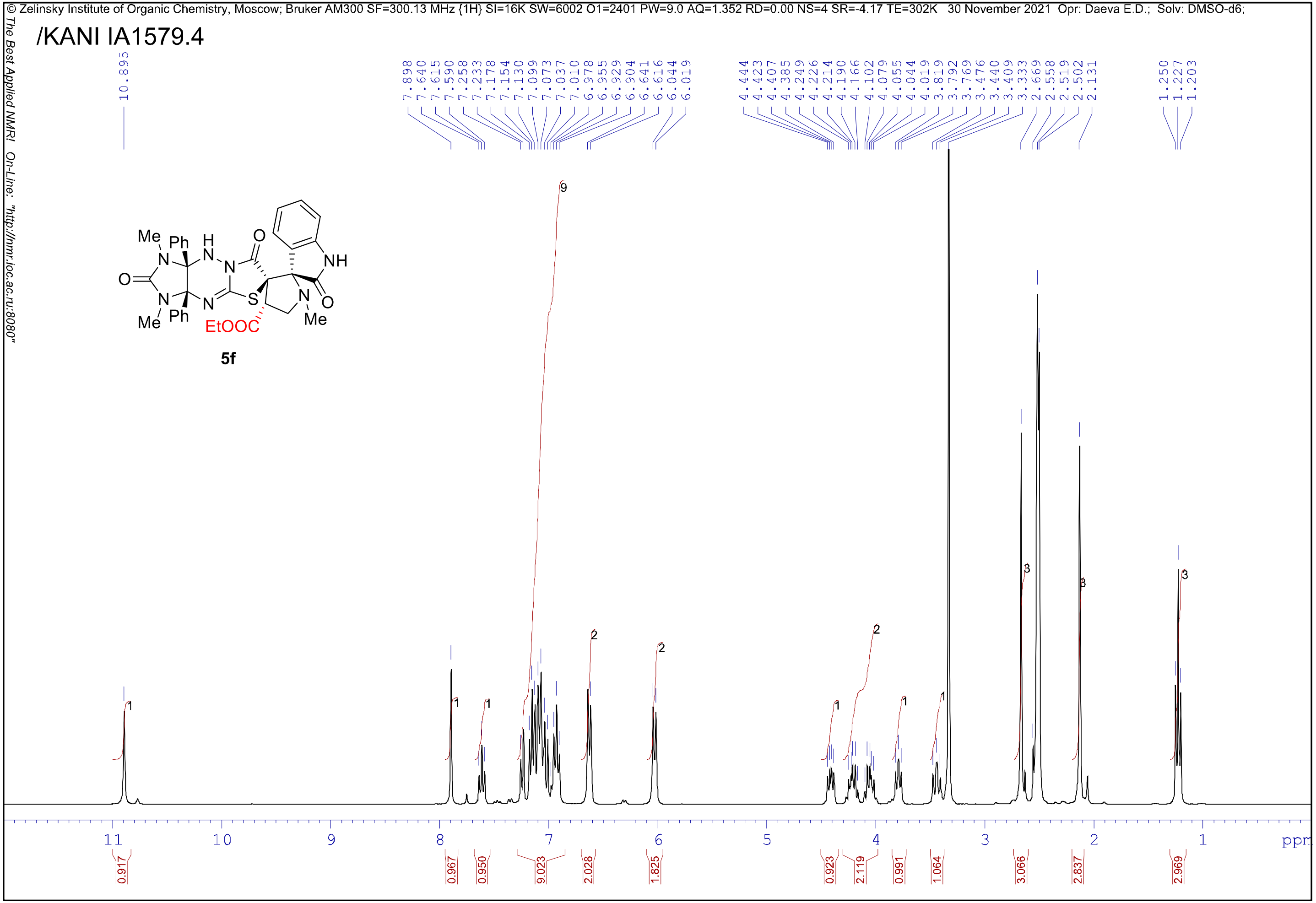
© Zelinsky Institute of Organic Chemistry, Moscow; Bruker AM300 SF=300.13 MHz {1H} SI=16K SW=6002 O1=2401 PW=9.0 AQ=1.352 RD=0.00 NS=4 SR=-4.17 TE=302K 30 November 2021 Opr: Daeva E.D.; Solv: DMSO-d6;
/KANI IA1579.4
The Best Applied NMR! On-Line: "http://nmr.ioc.ac.ru:8080"
10.895
7.898 7.640 7.615 7.590 7.258 7.233 7.178 7.154 7.130 7.099 7.073 7.037 7.010 6.978 6.955 6.929 6.904 6.641 6.616 6.044 6.019
4.444 4.423 4.407 4.385 4.249 4.226 4.214 4.190 4.166 4.102 4.079 4.055 4.044 4.019 3.819 3.792 3.769 3.476 3.440 3.409 3.333 2.669 2.558 2.519 2.502 2.131
1.250 1.227 1.203
Me
Ph
H
O
N
N
N
NH
O
N
N
S
N
O
Me
Ph
EtOOC
Me
5f
0.917
0.967
0.950
9.023
2.028
1.825
0.923
2.119
0.991
1.064
3.066
2.837
2.969
ppm

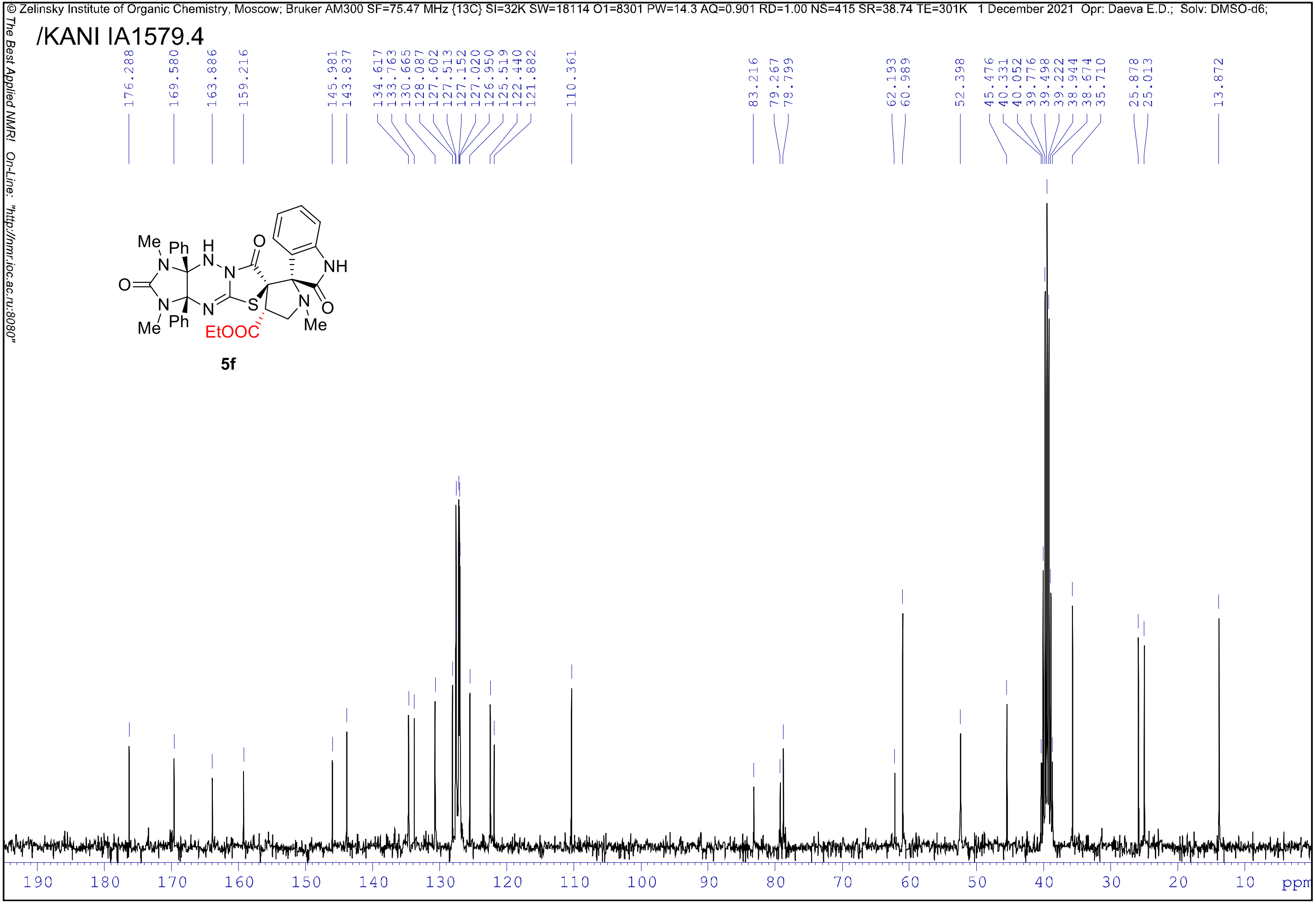
© Zelinsky Institute of Organic Chemistry, Moscow; Bruker AM300 SF=75.47 MHz {13C} SI=32K SW=18114 O1=8301 PW=14.3 AQ=0.901 RD=1.00 NS=415 SR=38.74 TE=301K 1 December 2021 Opr: Daeva E.D.; Solv: DMSO-d6;
The Best Applied NMR! On-Line: "http://nmr.ioc.ac.ru:8080"
/KANI IA1579.4
176.288
169.580
163.886
159.216
145.981
143.837
134.617
133.763
130.665
128.087
127.602
127.513
127.152
127.020
126.950
125.519
122.440
121.882
110.361
83.216
79.267
78.799
62.193
60.989
52.398
45.476
40.331
40.052
39.776
39.498
39.222
38.944
38.674
35.710
25.878
25.013
13.872
EtOOC
5f
190 180 170 160 150 140 130 120 110 100 90 80 70 60 50 40 30 20 10 ppm

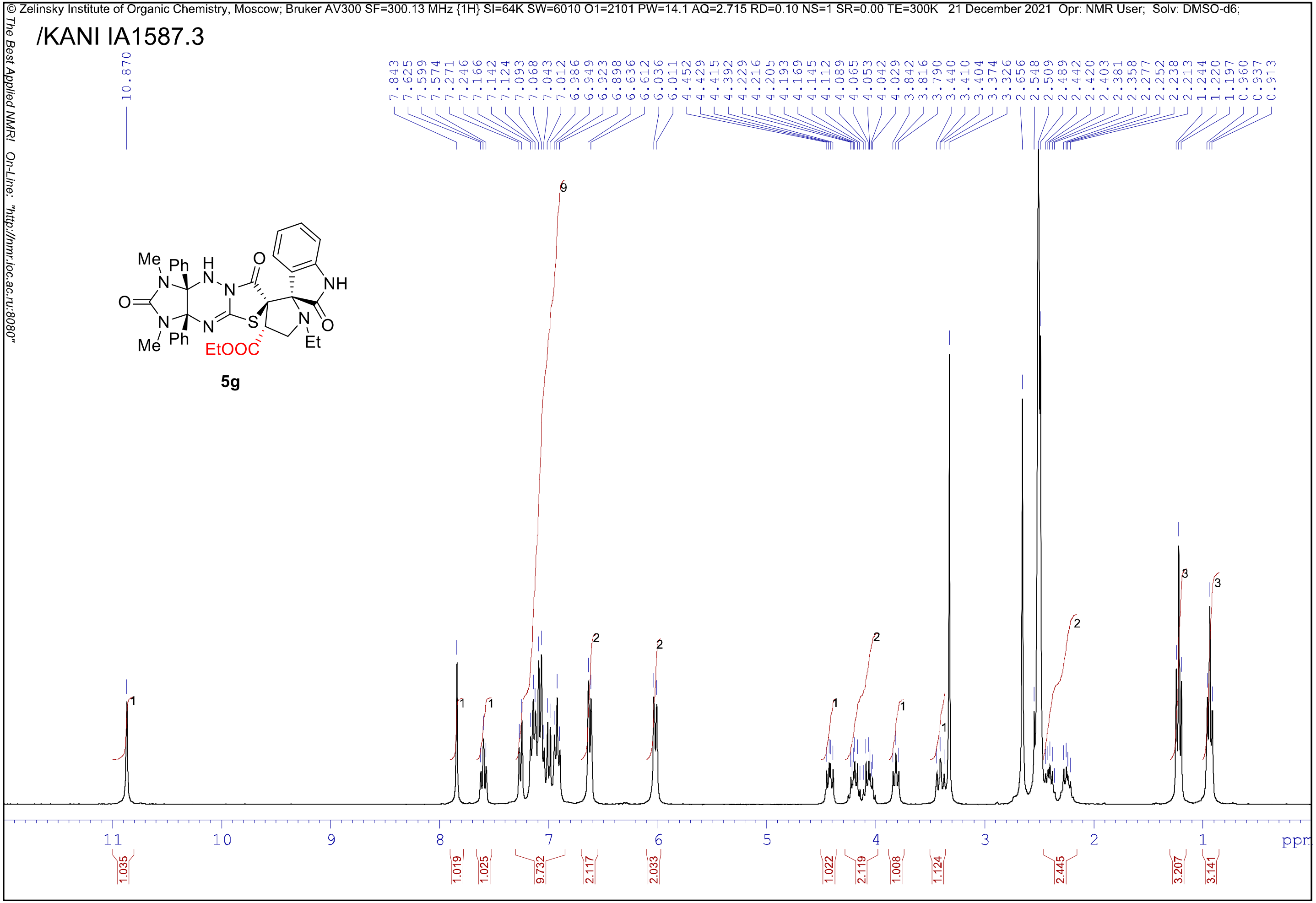
© Zelinsky Institute of Organic Chemistry, Moscow; Bruker AV300 SF=300.13 MHz {1H} SI=64K SW=6010 O1=2101 PW=14.1 AQ=2.715 RD=0.10 NS=1 SR=0.00 TE=300K 21 December 2021 Opr: NMR User; Solv: DMSO-d6;
The Best Applied NMR! On-Line: "http://nmr.ioc.ac.ru:8080"
/KANI IA1587.3
10.870
7.843 7.625 7.599 7.574 7.271 7.246 7.166 7.142 7.124 7.093 7.068 7.043 7.012 6.986 6.949 6.923 6.898 6.636 6.612 6.036 6.011 4.452 4.429 4.415 4.392 4.229 4.216 4.205 4.193 4.169 4.145 4.112 4.089 4.065 4.053 4.042 4.029 3.842 3.816 3.790 3.440 3.410 3.404 3.374 3.326 2.656 2.548 2.509 2.489 2.442 2.420 2.403 2.381 2.358 2.277 2.252 2.238 2.213 1.244 1.220 1.197 0.960 0.937 0.913
Me
Ph
H
N
O
N
N
NH
O
N
N
S
N
O
Me
Ph
EtOOC
Et
5g
1.035 1.019 1.025 9.732 2.117 2.033 1.022 2.119 1.008 1.124 2.445 3.207 3.141
ppm

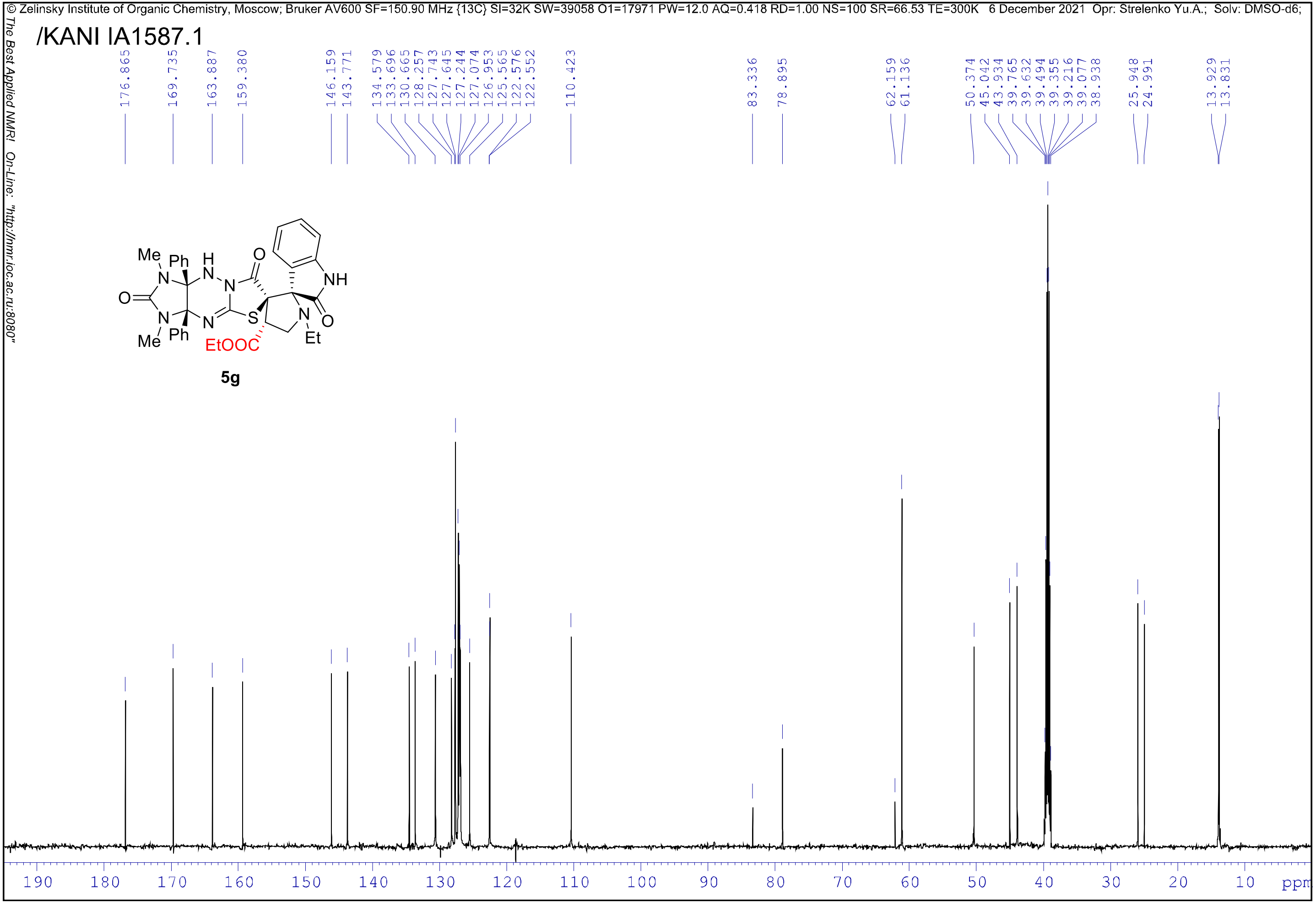
© Zelinsky Institute of Organic Chemistry, Moscow; Bruker AV600 SF=150.90 MHz {13C} SI=32K SW=39058 O1=17971 PW=12.0 AQ=0.418 RD=1.00 NS=100 SR=66.53 TE=300K 6 December 2021 Opr: Strelenko Yu.A.; Solv: DMSO-d6;
The Best Applied NMR! On-Line: "http://nmr.ioc.ac.ru:8080"
/KANI IA1587.1
176.865
169.735
163.887
159.380
146.159
143.771
134.579
133.696
130.665
128.257
127.743
127.645
127.244
127.074
126.953
125.565
122.576
122.552
110.423
83.336
78.895
62.159
61.136
50.374
45.042
43.934
39.765
39.632
39.494
39.355
39.216
39.077
38.938
25.948
24.991
13.929
13.831
Me
Ph
H
N
O
NH
S
EtOOC
Et
5g
190 180 170 160 150 140 130 120 110 100 90 80 70 60 50 40 30 20 10 ppm

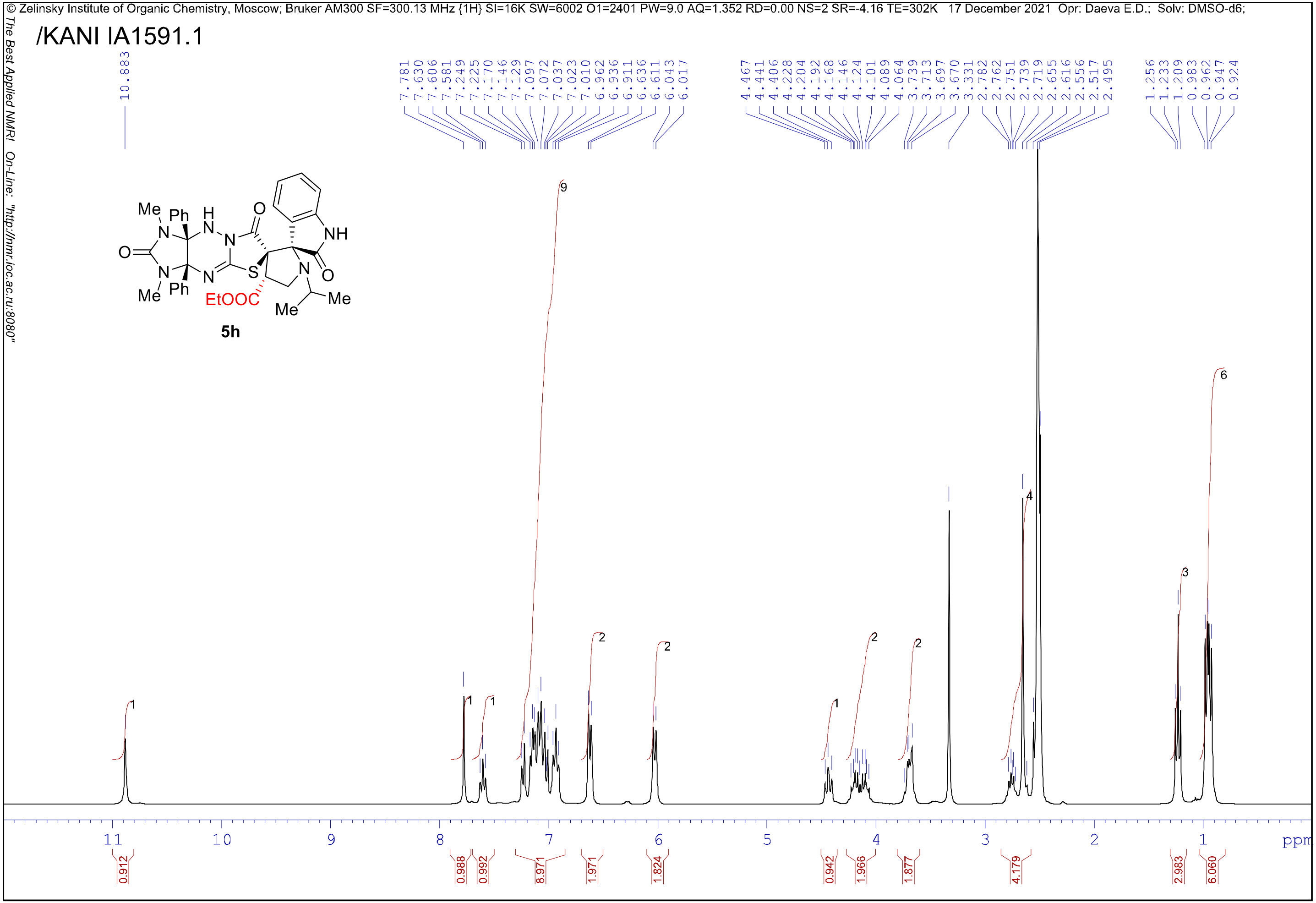

© Zelinsky Institute of Organic Chemistry, Moscow; Bruker AM300 SF=300.13 MHz {1H} SI=16K SW=6002 O1=2401 PW=9.0 AQ=1.352 RD=0.00 NS=2 SR=-4.16 TE=302K 17 December 2021 Opr: Daeva E.D.; Solv: DMSO-d6;
The Best Applied NMR! On-Line: "http://nmr.ioc.ac.ru:8080"
/KANI IA1591.1
10.883
7.781 7.630 7.606 7.581 7.249 7.225 7.170 7.146 7.129 7.097 7.072 7.037 7.023 7.010 6.962 6.936 6.911 6.636 6.611 6.043 6.017
4.467 4.441 4.406 4.228 4.204 4.192 4.168 4.146 4.124 4.101 4.089 4.064 3.739 3.713 3.697 3.670 3.331 2.782 2.762 2.751 2.739 2.719 2.655 2.616 2.556 2.517 2.495
1.256 1.233 1.209 0.983 0.962 0.947 0.924
5h
ppm
0.912 0.988 0.992 8.971 1.971 1.824 0.942 1.966 1.877 4.179 2.983 6.060

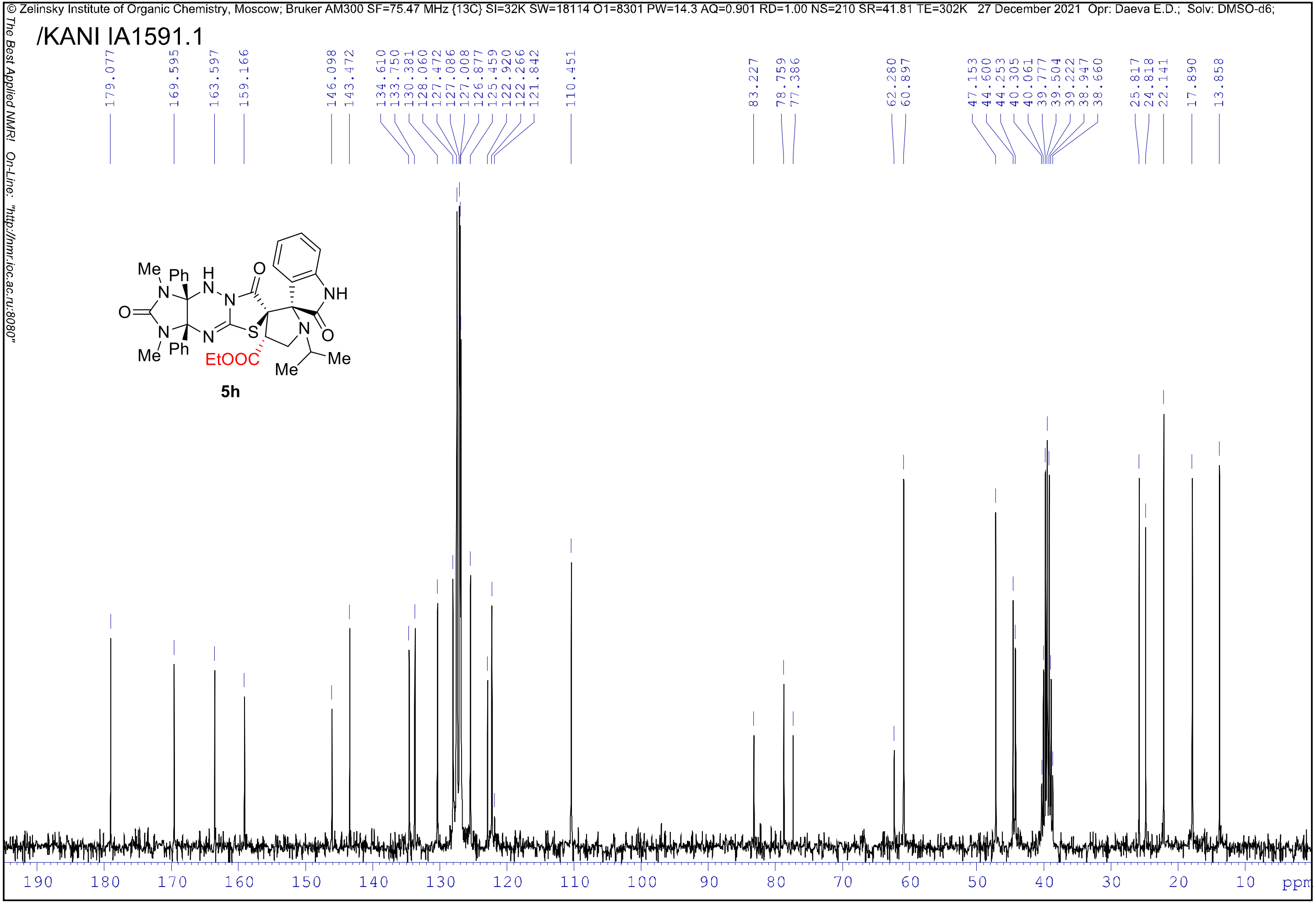
© Zelinsky Institute of Organic Chemistry, Moscow; Bruker AM300 SF=75.47 MHz {13C} SI=32K SW=18114 O1=8301 PW=14.3 AQ=0.901 RD=1.00 NS=210 SR=41.81 TE=302K 27 December 2021 Opr: Daeva E.D.; Solv: DMSO-d6;
The Best Applied NMR! On-Line: "http://nmr.ioc.ac.ru:8080"
/KANI IA1591.1
179.077
169.595
163.597
159.166
146.098
143.472
134.610
133.750
130.381
128.060
127.472
127.086
127.008
126.877
125.459
122.920
122.266
121.842
110.451
83.227
78.759
77.386
62.280
60.897
47.153
44.600
44.253
40.305
40.061
39.777
39.504
39.222
38.947
38.660
25.817
24.818
22.141
17.890
13.858
Me
Ph
H
N
O
NH
EtOOC
S
Me
5h
190 180 170 160 150 140 130 120 110 100 90 80 70 60 50 40 30 20 10 ppm

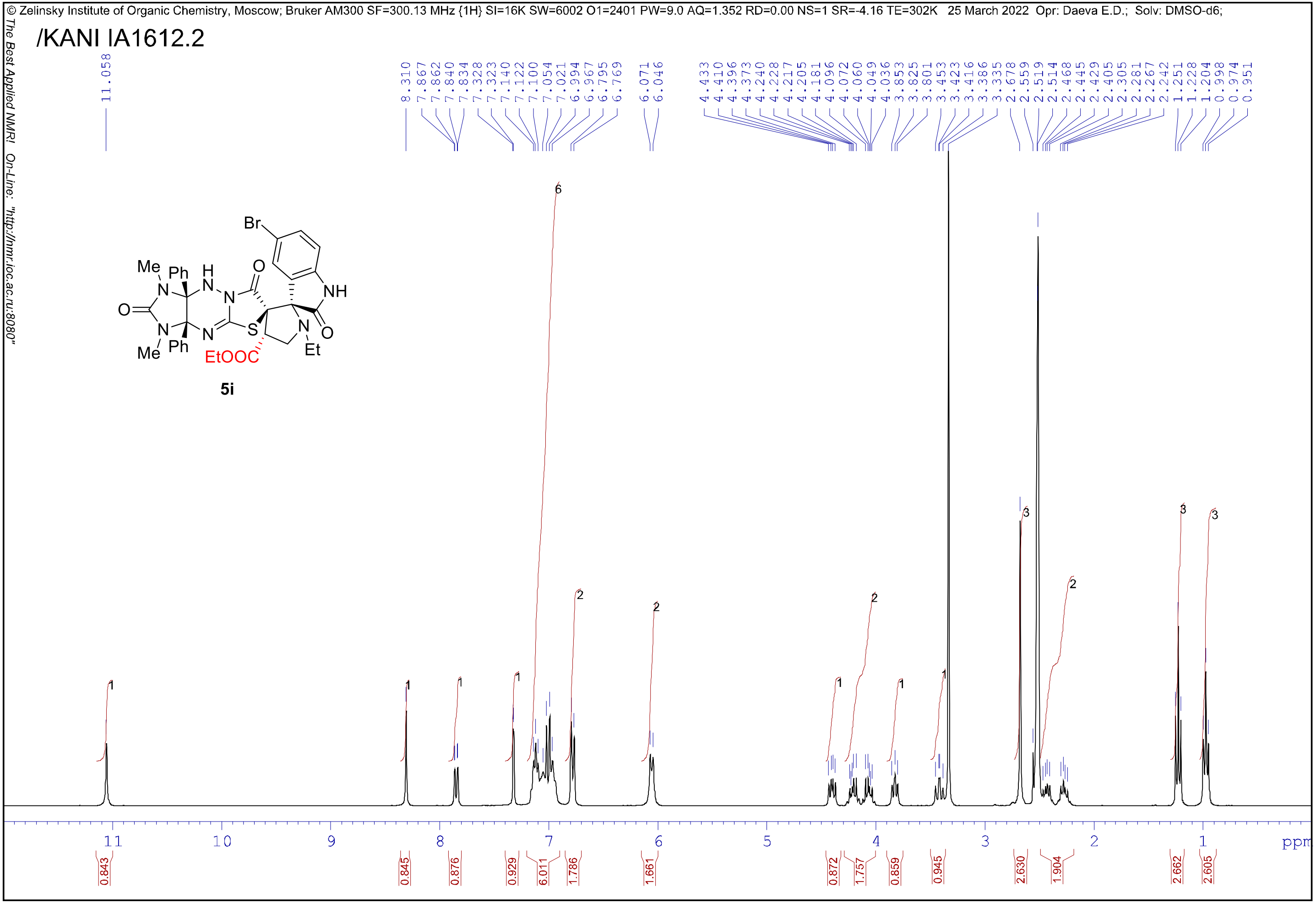
© Zelinsky Institute of Organic Chemistry, Moscow; Bruker AM300 SF=300.13 MHz {1H} SI=16K SW=6002 O1=2401 PW=9.0 AQ=1.352 RD=0.00 NS=1 SR=-4.16 TE=302K 25 March 2022 Opr: Daeva E.D.; Solv: DMSO-d6;
The Best Applied NMR! On-Line: "http://nmr.ioc.ac.ru:8080"
/KANI IA1612.2
11.058
8.310 7.867 7.862 7.840 7.834 7.328 7.323 7.140 7.122 7.100 7.054 7.021 6.994 6.967 6.795 6.769
6.071 6.046
4.433 4.410 4.396 4.373 4.240 4.228 4.217 4.205 4.181 4.096 4.072 4.060 4.049 4.036 3.853 3.825 3.801 3.453 3.423 3.416 3.386 3.335 2.678 2.559 2.519 2.514 2.468 2.445 2.429 2.405 2.305 2.281 2.267 2.242 1.251 1.228 1.204 0.998 0.974 0.951
Br
Me
Ph
H
N
O
NH
EtOOC
S
Et
5i
ppm
0.843 0.845 0.876 0.929 6.011 1.786 1.661 0.872 1.757 0.859 0.945 2.630 1.904 2.662 2.605

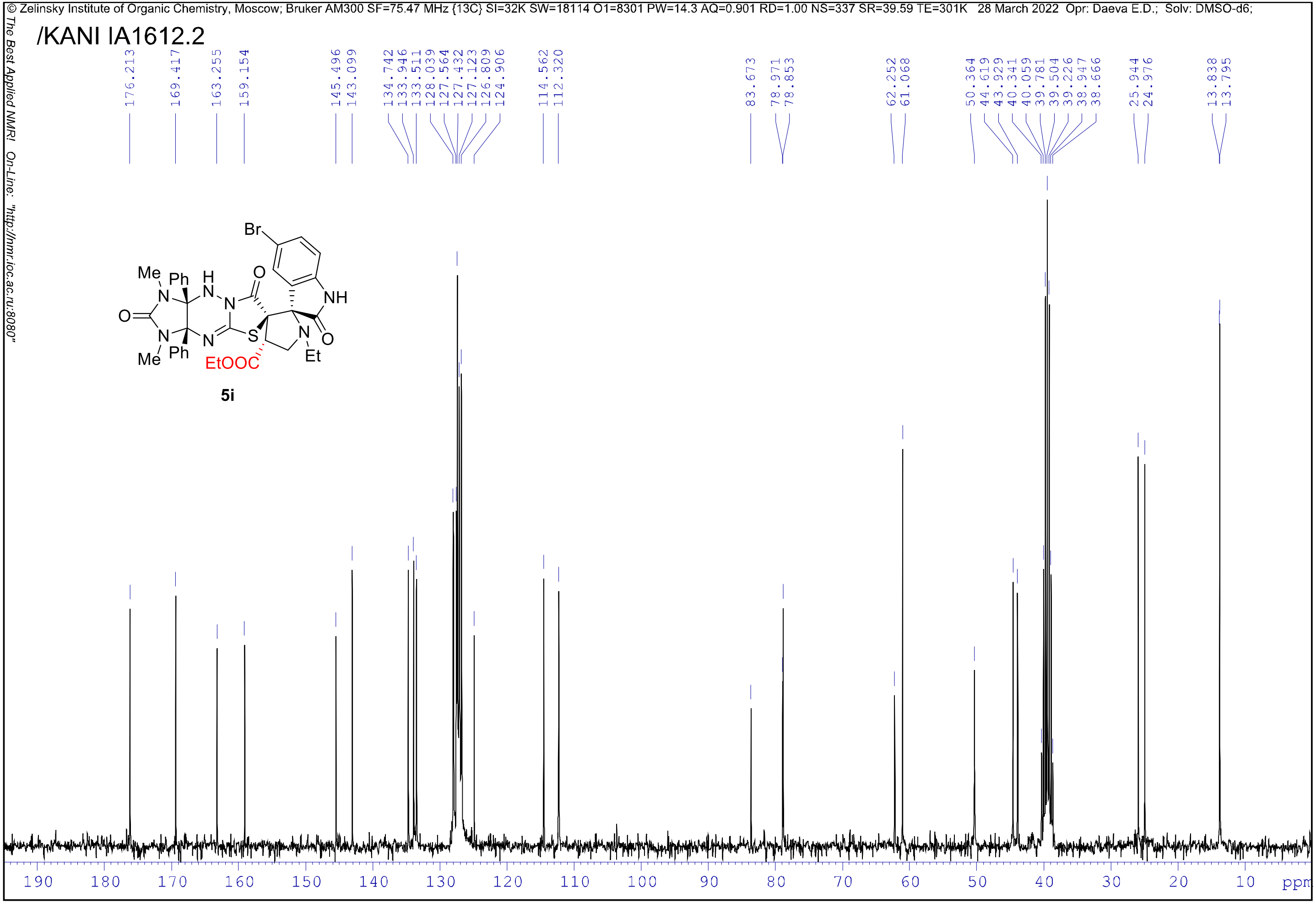
© Zelinsky Institute of Organic Chemistry, Moscow; Bruker AM300 SF=75.47 MHz {13C} SI=32K SW=18114 O1=8301 PW=14.3 AQ=0.901 RD=1.00 NS=337 SR=39.59 TE=301K 28 March 2022 Opr: Daeva E.D.; Solv: DMSO-d6;
The Best Applied NMR! On-Line: "http://nmr.ioc.ac.ru:8080"
/KANI IA1612.2
176.213
169.417
163.255
159.154
145.496
143.099
134.742
133.946
133.511
128.039
127.564
127.432
127.123
126.809
124.906
114.562
112.320
83.673
78.971
78.853
62.252
61.068
50.364
44.619
43.929
40.341
40.059
39.781
39.504
39.226
38.947
38.666
25.944
24.976
13.838
13.795
Br
Me
Ph
H
N
O
NH
EtOOC
Et
S
5i
190 180 170 160 150 140 130 120 110 100 90 80 70 60 50 40 30 20 10 ppm

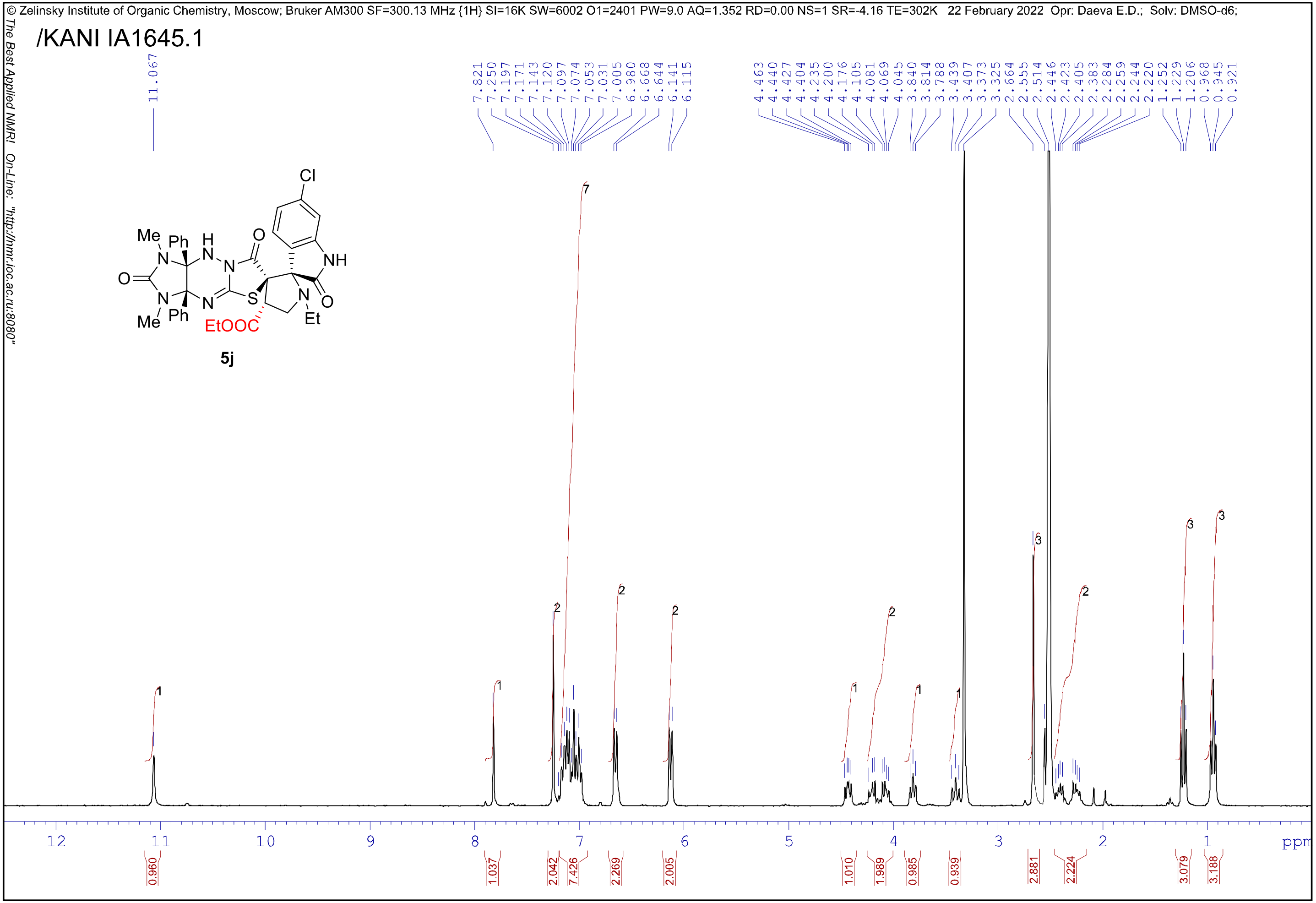
© Zelinsky Institute of Organic Chemistry, Moscow; Bruker AM300 SF=300.13 MHz {1H} SI=16K SW=6002 O1=2401 PW=9.0 AQ=1.352 RD=0.00 NS=1 SR=-4.16 TE=302K 22 February 2022 Opr: Daeva E.D.; Solv: DMSO-d6;
The Best Applied NMR! On-Line: "http://nmr.ioc.ac.ru:8080"
/KANI IA1645.1
11.067
7.821 7.250 7.197 7.171 7.143 7.120 7.097 7.074 7.053 7.031 7.005 6.980 6.668 6.644 6.141 6.115
4.463 4.440 4.427 4.404 4.235 4.200 4.176 4.105 4.081 4.069 4.045 3.840 3.814 3.788 3.439 3.407 3.373 3.325 2.664 2.555 2.514 2.446 2.423 2.405 2.383 2.284 2.259 2.244 2.220 1.252 1.229 1.206 0.968 0.945 0.921
Cl
NH
O
Me
Ph
H
N
O
S
N
Et
EtOOC
5j
0.960 1.037 2.042 7.426 2.269 2.005 1.010 1.989 0.985 0.939 2.881 2.224 3.079 3.188
12 11 10 9 8 7 6 5 4 3 2 1 ppm

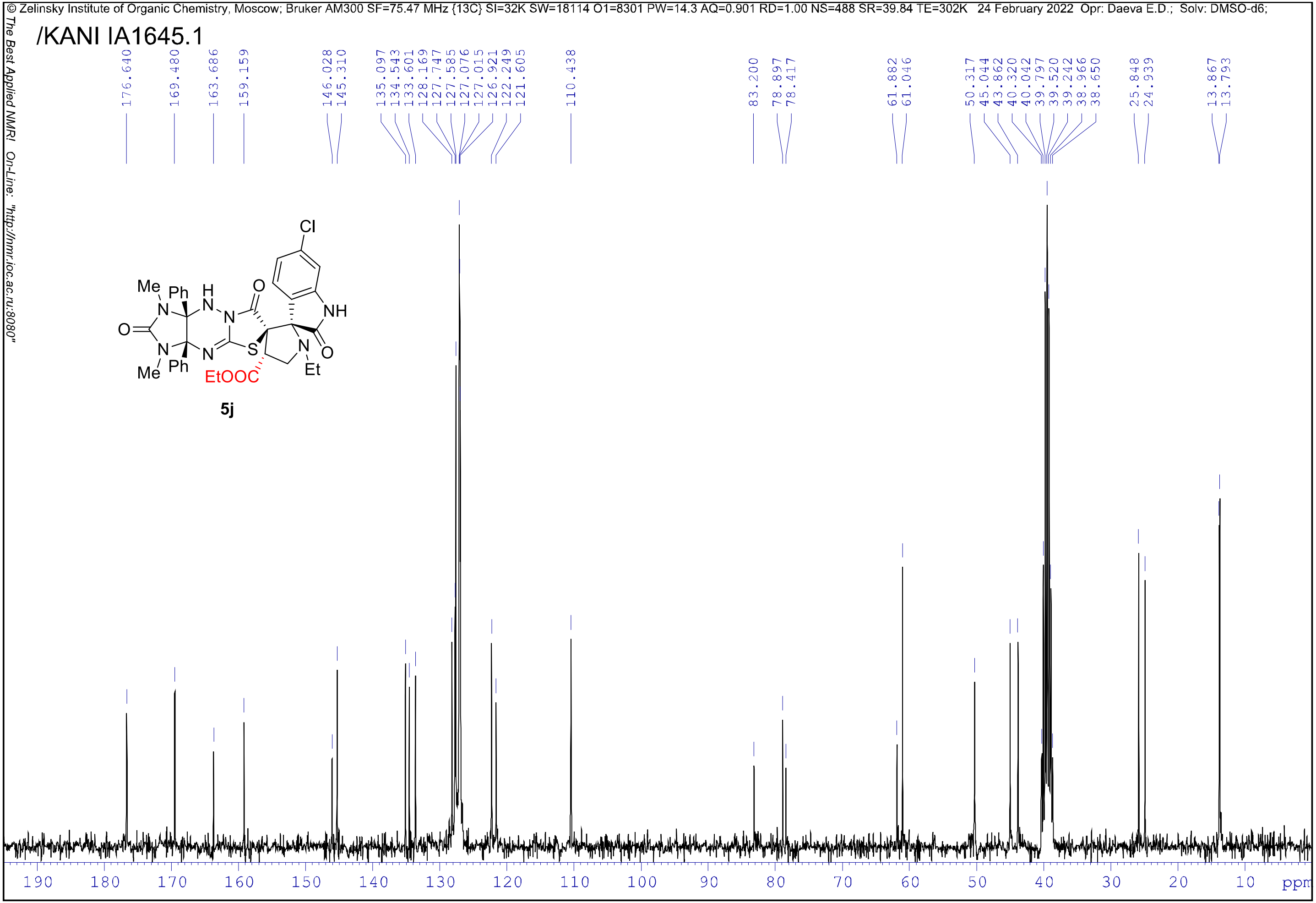
© Zelinsky Institute of Organic Chemistry, Moscow; Bruker AM300 SF=75.47 MHz {13C} SI=32K SW=18114 O1=8301 PW=14.3 AQ=0.901 RD=1.00 NS=488 SR=39.84 TE=302K 24 February 2022 Opr: Daeva E.D.; Solv: DMSO-d6;
The Best Applied NMR! On-Line: "http://nmr.ioc.ac.ru:8080"
/KANI IA1645.1
176.640
169.480
163.686
159.159
146.028
145.310
135.097
134.543
133.601
128.169
127.747
127.585
127.076
127.015
126.921
122.249
121.605
110.438
83.200
78.897
78.417
61.882
61.046
50.317
45.044
43.862
40.320
40.042
39.797
39.520
39.242
38.966
38.650
25.848
24.939
13.867
13.793
Cl
Me
Ph
H
N
O
NH
EtOOC
S
Et
5j
190
180
170
160
150
140
130
120
110
100
90
80
70
60
50
40
30
20
10
ppm

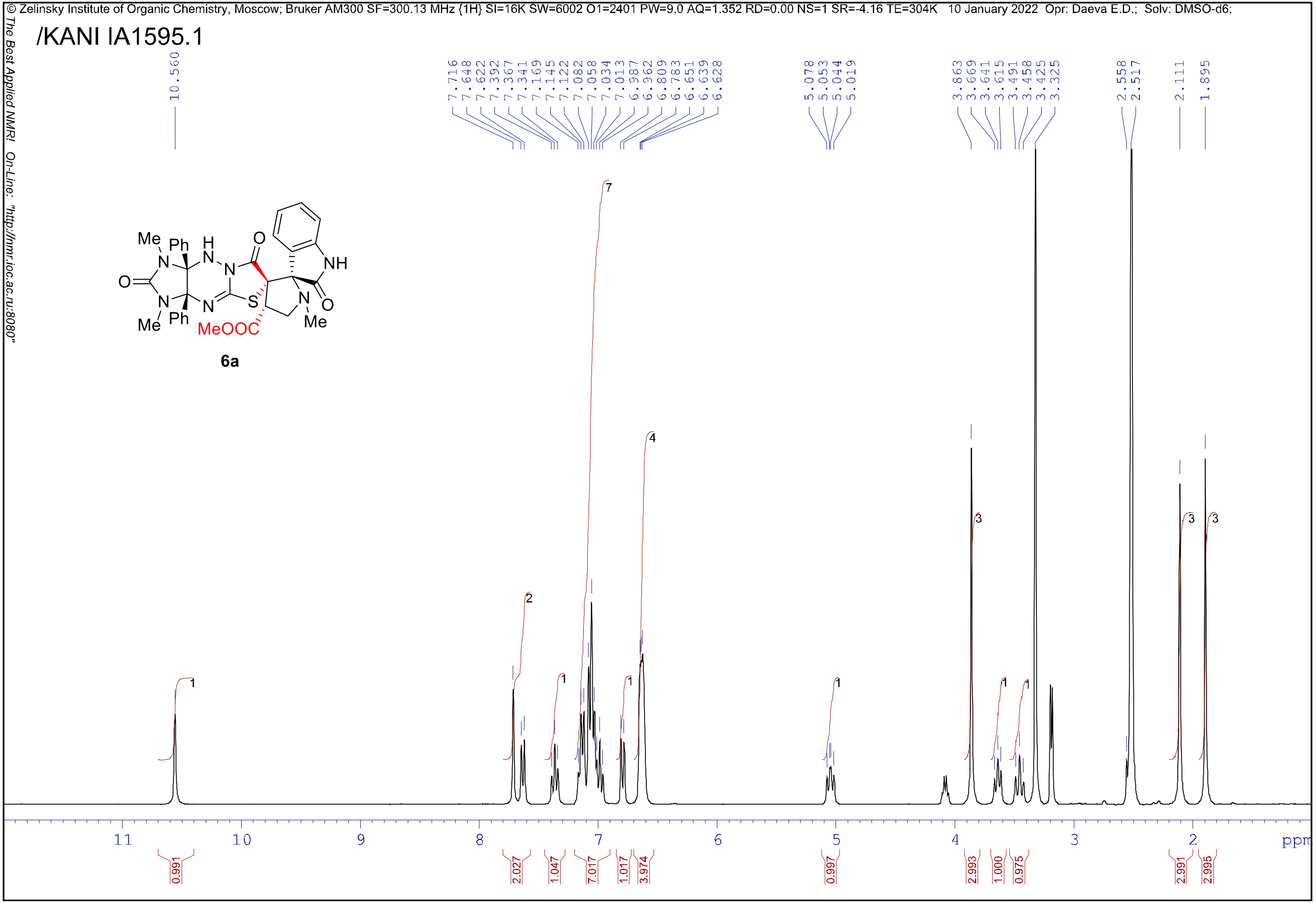
© Zelinsky Institute of Organic Chemistry, Moscow; Bruker AM300 SF=300.13 MHz {1H} SI=16K SW=6002 O1=2401 PW=9.0 AQ=1.352 RD=0.00 NS=1 SR=-4.16 TE=304K 10 January 2022 Opr: Daeva E.D.; Solv: DMSO-d6;
The Best Applied NMR! On-Line: "http://nmr.ioc.ac.ru:8080"
/KANI IA1595.1
10.560
7.716 7.648 7.622 7.392 7.367 7.341 7.169 7.145 7.122 7.082 7.058 7.034 7.013 6.987 6.962 6.809 6.783 6.651 6.639 6.628
5.078 5.053 5.044 5.019
3.863 3.669 3.641 3.615 3.491 3.458 3.425 3.325
2.558 2.517
2.111
1.895
MeOOC
6a
11 10 9 8 7 6 5 4 3 2 ppm
0.991 2.027 1.047 7.017 1.017 3.974 0.997 2.993 1.000 0.975 2.991 2.995

© Zelinsky Institute of Organic Chemistry, Moscow; Bruker AM300 SF=75.47 MHz {13C}APT SI=32K SW=18114 O1=8301 PW=13.0 AQ=0.900 RD=1.00 NS=365 SR=38.86 TE=304K 10 January 2022 Opr: Daeva E.D.; Solv: DMSO-d6;

The Best Applied NMR! On-Line: "http://nmr.ioc.ac.ru:8080"

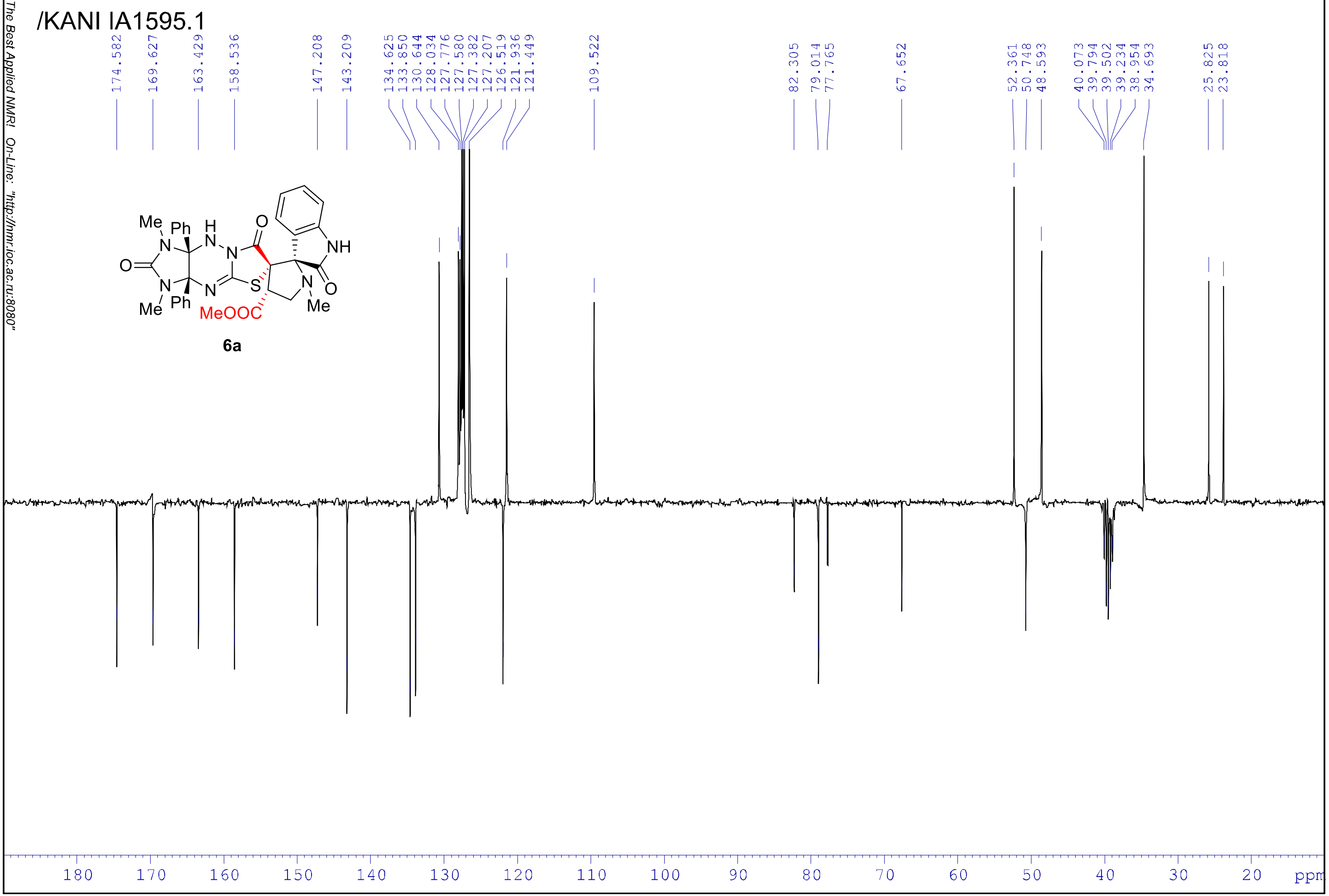

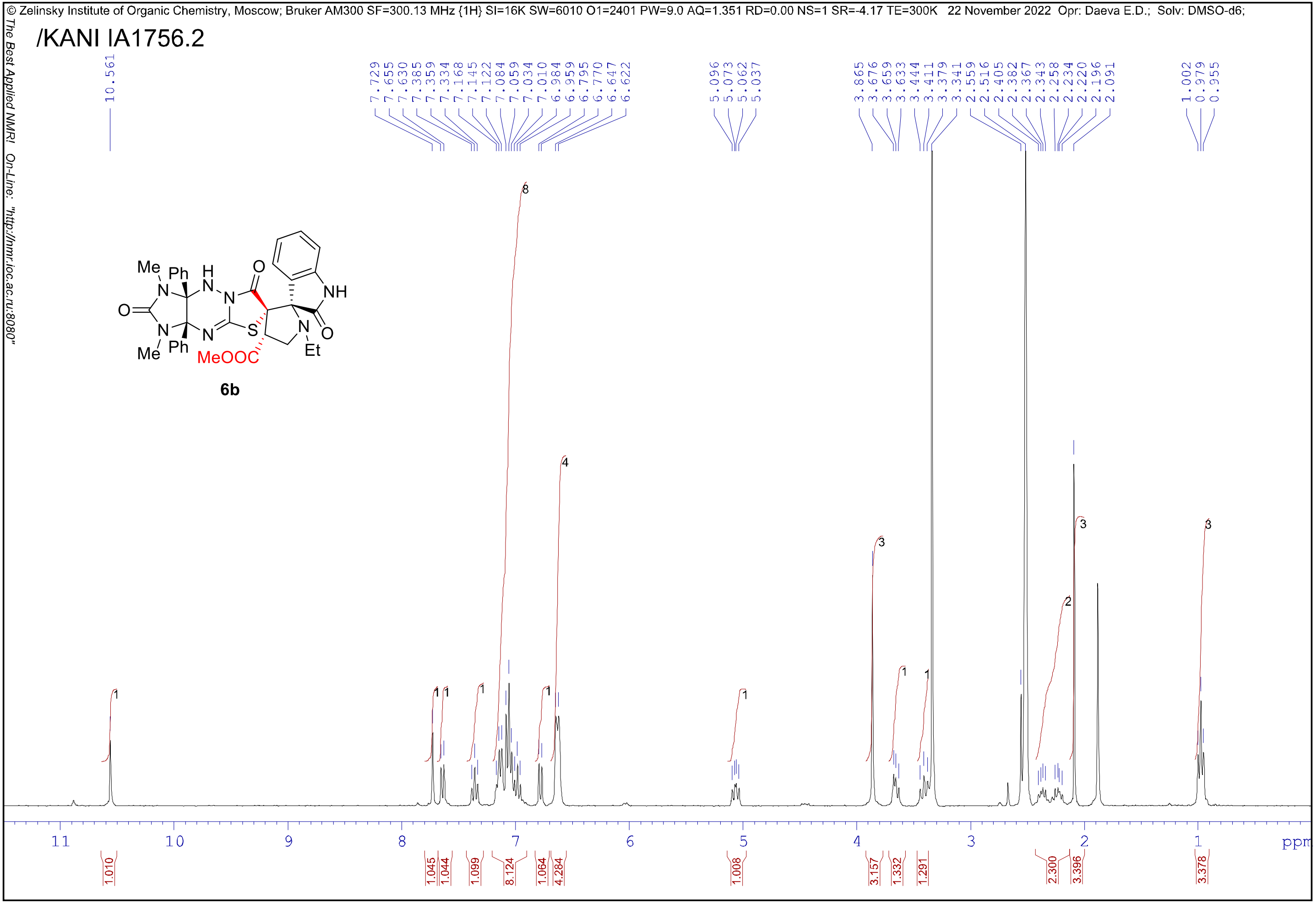
© Zelinsky Institute of Organic Chemistry, Moscow; Bruker AM300 SF=300.13 MHz {1H} SI=16K SW=6010 O1=2401 PW=9.0 AQ=1.351 RD=0.00 NS=1 SR=-4.17 TE=300K 22 November 2022 Opr: Daeva E.D.; Solv: DMSO-d6;
The Best Applied NMR! On-Line: "http://nmr.ioc.ac.ru:8080"
/KANI IA1756.2
10.561
7.729 7.655 7.630 7.385 7.359 7.334 7.168 7.145 7.122 7.084 7.059 7.034 7.010 6.984 6.959 6.795 6.770 6.647 6.622
5.096 5.073 5.062 5.037
3.865 3.676 3.659 3.633 3.444 3.411 3.379 3.341 2.559 2.516 2.405 2.382 2.367 2.343 2.258 2.234 2.220 2.196 2.091
1.002 0.979 0.955
MeOOC
6b
ppm
1.010 1.045 1.044 1.099 8.124 1.064 4.284 1.008 3.157 1.332 1.291 2.300 3.396 3.378

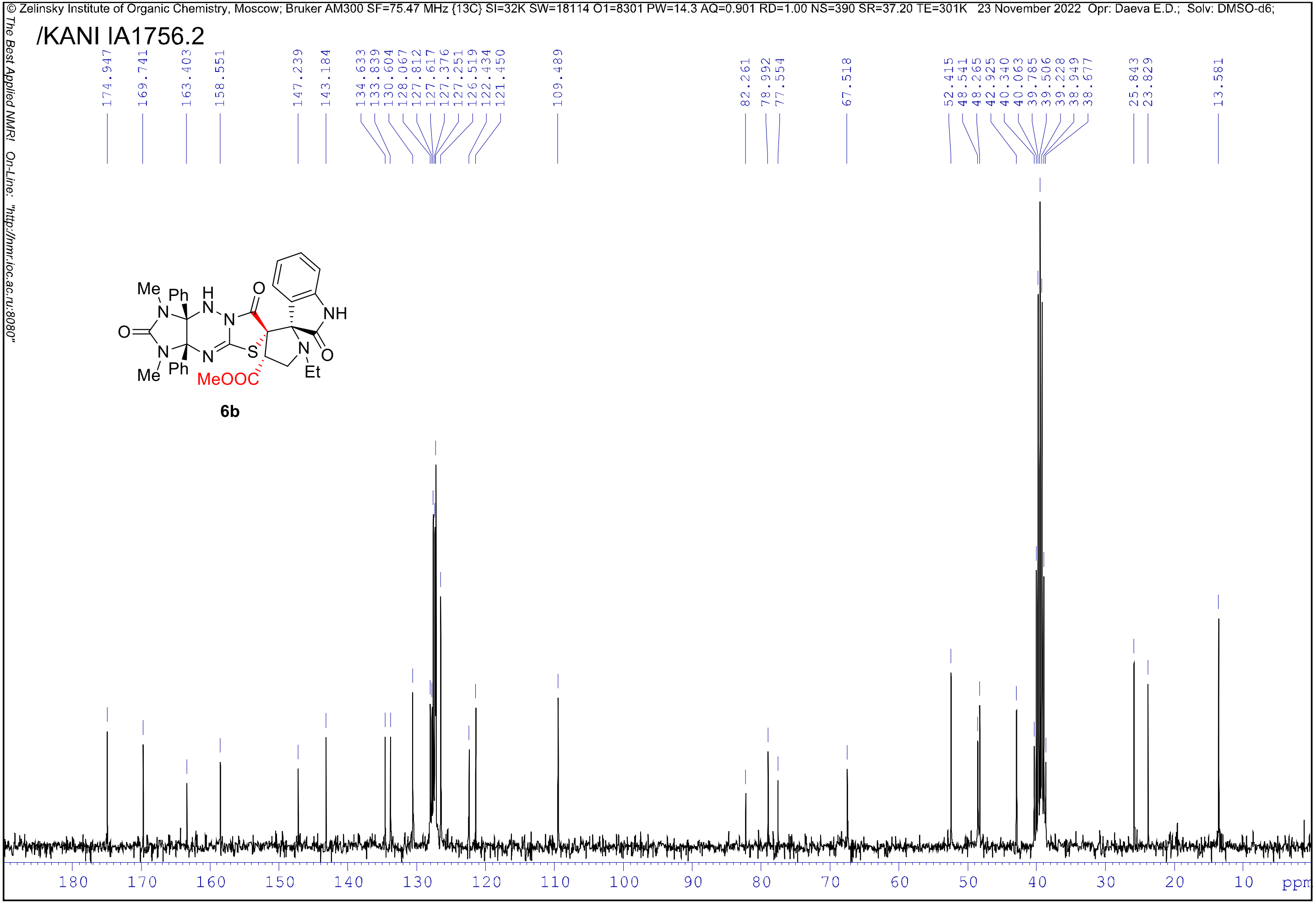
© Zelinsky Institute of Organic Chemistry, Moscow; Bruker AM300 SF=75.47 MHz {13C} SI=32K SW=18114 O1=8301 PW=14.3 AQ=0.901 RD=1.00 NS=390 SR=37.20 TE=301K 23 November 2022 Opr: Daeva E.D.; Solv: DMSO-d6;
The Best Applied NMR! On-Line: "http://nmr.ioc.ac.ru:8080"
/KANI IA1756.2
174.947
169.741
163.403
158.551
147.239
143.184
134.633
133.839
130.604
128.067
127.812
127.617
127.376
127.251
126.519
122.434
121.450
109.489
82.261
78.992
77.554
67.518
52.415
48.541
48.265
42.925
40.340
40.063
39.785
39.506
39.228
38.949
38.677
25.843
23.829
13.581
180 170 160 150 140 130 120 110 100 90 80 70 60 50 40 30 20 10 ppm
6b

© Zelinsky Institute of Organic Chemistry, Moscow; Bruker AV300 SF=300.13 MHz {1H} SI=64K SW=6010 O1=2101 PW=14.1 AQ=3.317 RD=0.10 NS=4 SR=0.00 TE=300K 18 November 2022 Opr: NMR User; Solv: DMSO-d6;

The Best Applied NMR! On-Line: "http://nmr.ioc.ac.ru:8080"

/KANI VDB524.1.1

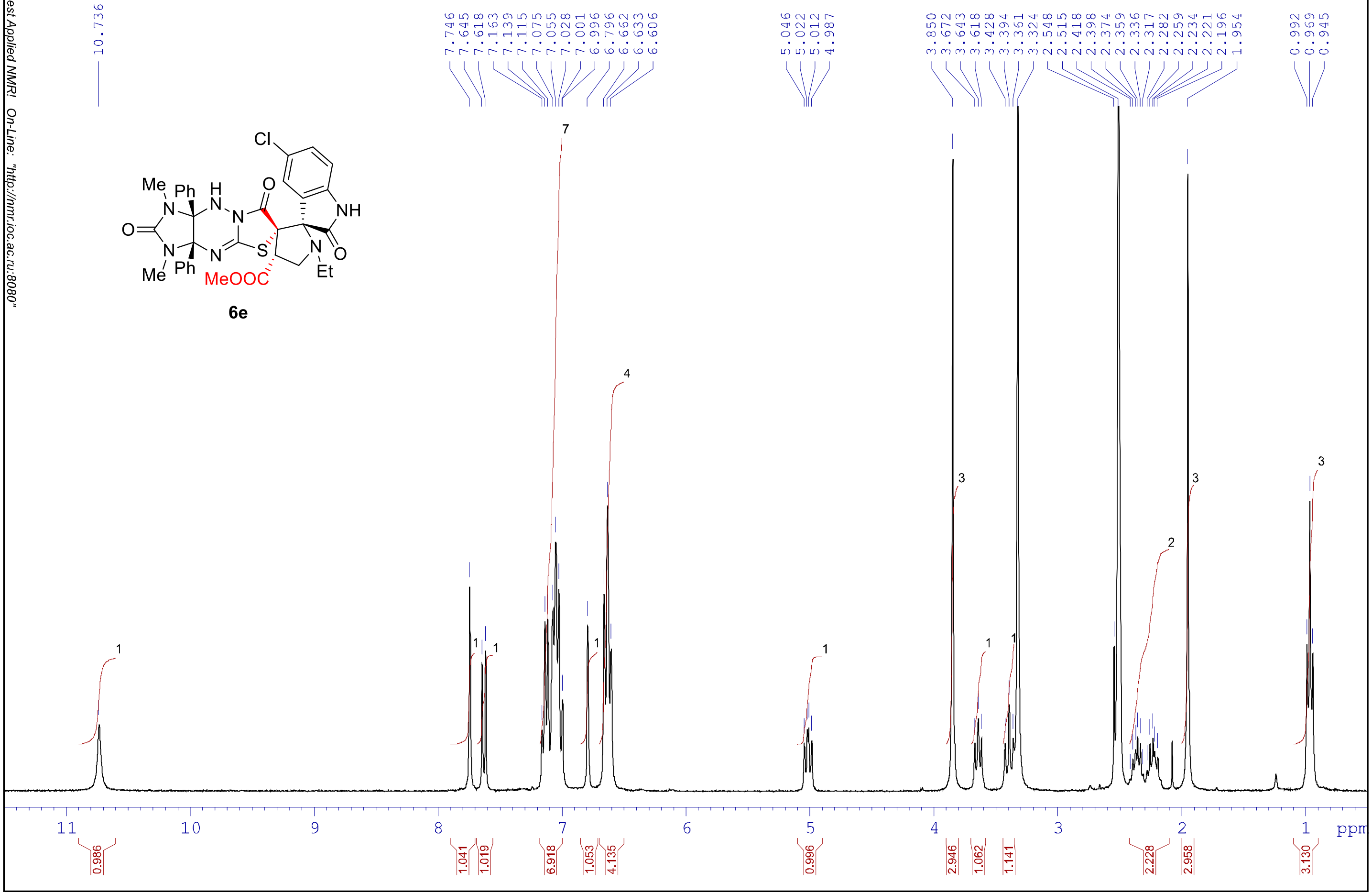

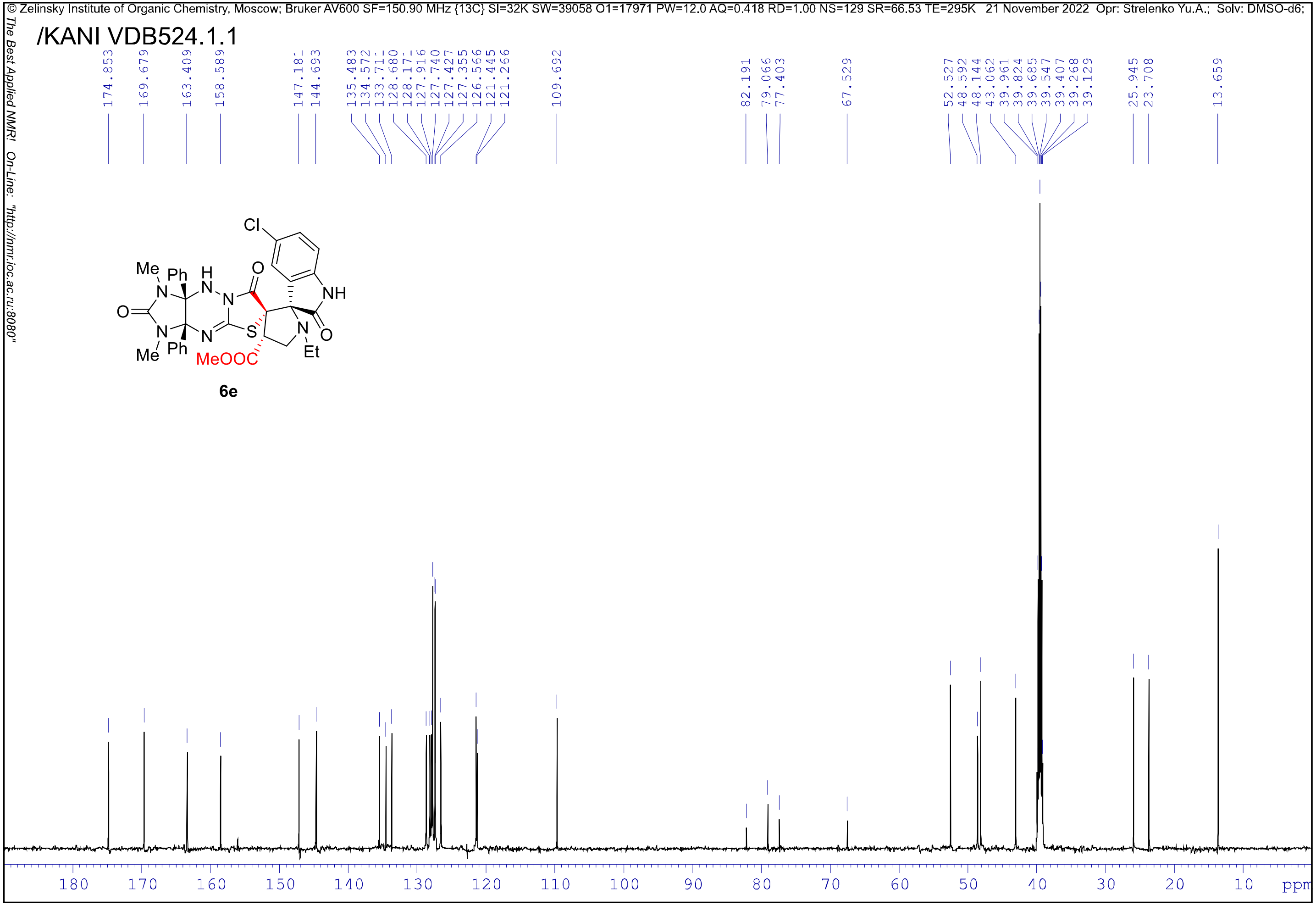
© Zelinsky Institute of Organic Chemistry, Moscow; Bruker AV600 SF=150.90 MHz {13C} SI=32K SW=39058 O1=17971 PW=12.0 AQ=0.418 RD=1.00 NS=129 SR=66.53 TE=295K 21 November 2022 Opr: Strelenko Yu.A.; Solv: DMSO-d6;
The Best Applied NMR! On-Line: "http://nmr.ioc.ac.ru:8080"
/KANI VDB524.1.1
174.853
169.679
163.409
158.589
147.181
144.693
135.483
134.572
133.711
128.680
128.171
127.916
127.740
127.427
127.355
126.566
121.445
121.266
109.692
82.191
79.066
77.403
67.529
52.527
48.592
48.144
43.062
39.961
39.824
39.685
39.547
39.407
39.268
39.129
25.945
23.708
13.659
Cl
Me
Ph
H
N
O
NH
MeOOC
S
Et
6e
180 170 160 150 140 130 120 110 100 90 80 70 60 50 40 30 20 10 ppm

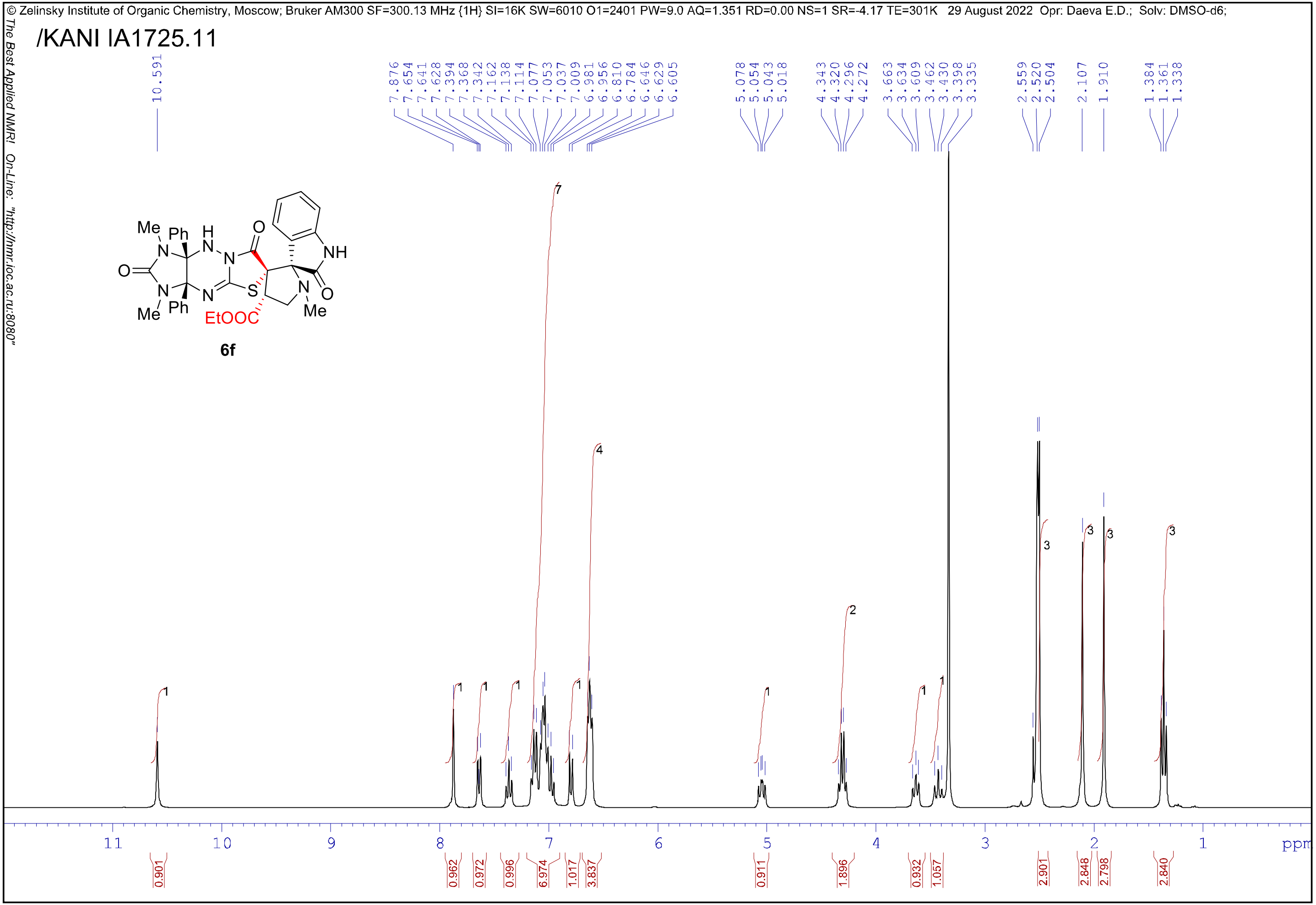
© Zelinsky Institute of Organic Chemistry, Moscow; Bruker AM300 SF=300.13 MHz {1H} SI=16K SW=6010 O1=2401 PW=9.0 AQ=1.351 RD=0.00 NS=1 SR=-4.17 TE=301K 29 August 2022 Opr: Daeva E.D.; Solv: DMSO-d6;
The Best Applied NMR! On-Line: "http://nmr.ioc.ac.ru:8080"
/KANI IA1725.11
10.591
7.876 7.654 7.641 7.628 7.394 7.368 7.342 7.162 7.138 7.114 7.077 7.053 7.037 7.009 6.981 6.956 6.810 6.784 6.646 6.629 6.605
5.078 5.054 5.043 5.018
4.343 4.320 4.296 4.272
3.663 3.634 3.609 3.462 3.430 3.398 3.335
2.559 2.520 2.504
2.107
1.910
1.384 1.361 1.338
Me
Ph
H
N
O
EtOOC
S
NH
Me
6f
ppm
0.901 0.962 0.972 0.996 6.974 1.017 3.837 0.911 1.896 0.932 1.057 2.901 2.848 2.798 2.840

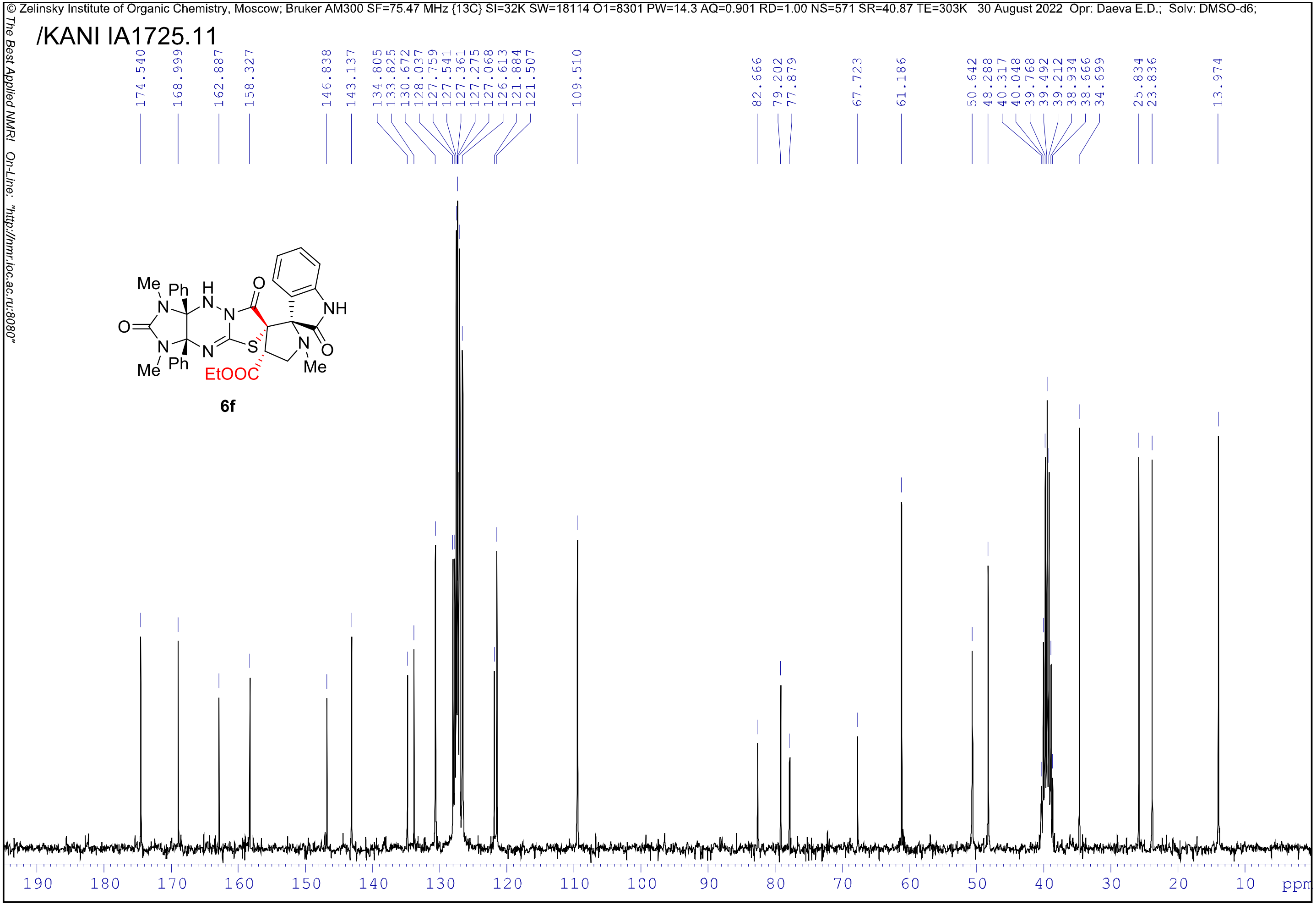

© Zelinsky Institute of Organic Chemistry, Moscow; Bruker AM300 SF=75.47 MHz {13C} SI=32K SW=18114 O1=8301 PW=14.3 AQ=0.901 RD=1.00 NS=571 SR=40.87 TE=303K 30 August 2022 Opr: Daeva E.D.; Solv: DMSO-d6;
The Best Applied NMR! On-Line: "http://nmr.ioc.ac.ru:8080"
/KANI IA1725.11
174.540
168.999
162.887
158.327
146.838
143.137
134.805
133.825
130.672
128.037
127.759
127.541
127.361
127.275
127.068
126.613
121.884
121.507
109.510
82.666
79.202
77.879
67.723
61.186
50.642
48.288
40.317
40.048
39.768
39.492
39.212
38.934
38.666
34.699
25.834
23.836
13.974
Me
Ph
H
N
O
NH
EtOOC
S
6f
190
180
170
160
150
140
130
120
110
100
90
80
70
60
50
40
30
20
10
ppm

© Zelinsky Institute of Organic Chemistry, Moscow; Bruker AM300 SF=300.13 MHz {1H} SI=32K SW=6010 O1=2401 PW=9.0 AQ=1.351 RD=0.00 NS=1 SR=-4.18 TE=302K 26 January 2023 Opr: Daeva E.D.; Solv: DMSO-d6;

The Best Applied NMR! On-Line: "http://nmr.ioc.ac.ru:8080"

/KANI IA1771.6

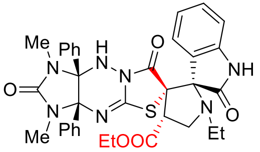

6g

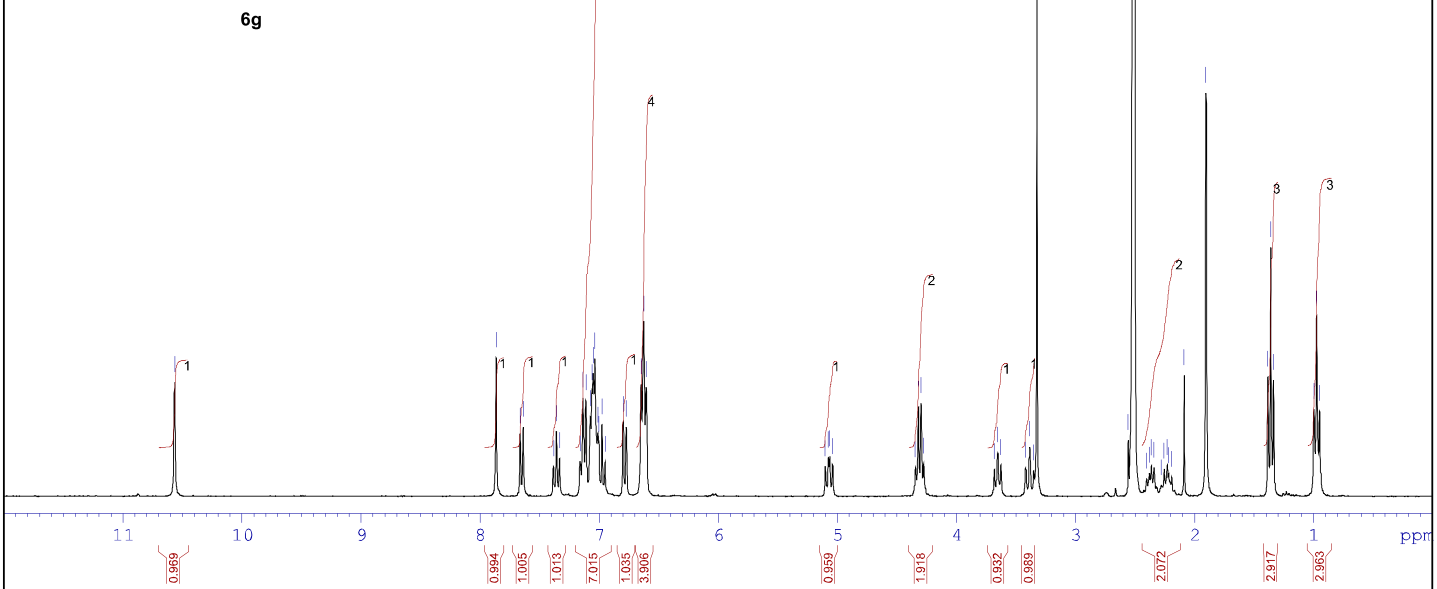

© Zelinsky Institute of Organic Chemistry, Moscow; Bruker AM300 SF=75.47 MHz {13C} SI=32K SW=18114 O1=8301 PW=14.3 AQ=0.901 RD=1.00 NS=591 SR=38.64 TE=301K 26 January 2023 Opr: Daeva E.D.; Solv: DMSO-d6;

The Best Applied NMR! On-Line: "http://nmr.ioc.ac.ru:8080"

/KANI IA1771.6

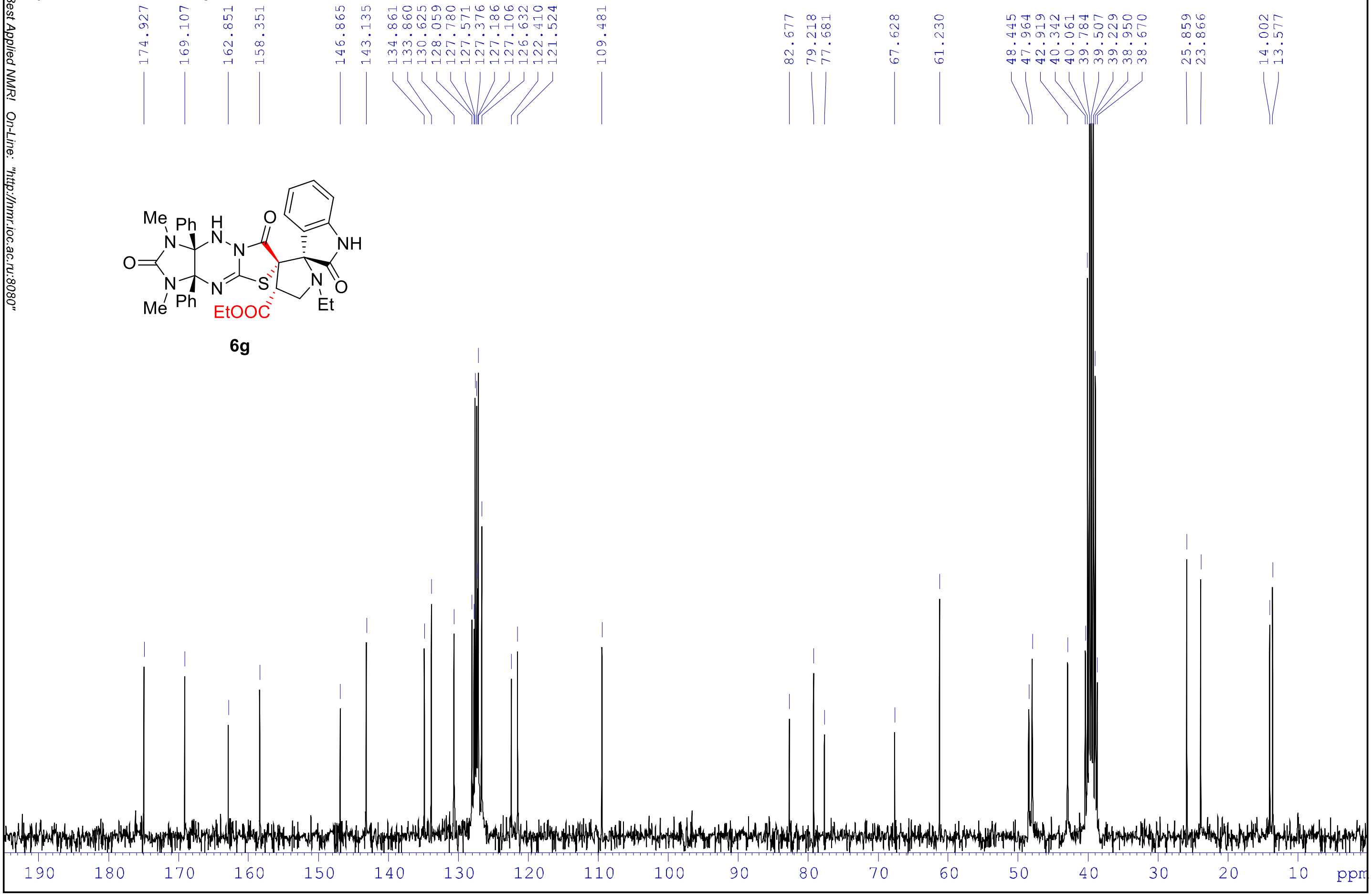

© Zelinsky Institute of Organic Chemistry, Moscow; Bruker AM300 SF=300.13 MHz {1H} SI=16K SW=6002 O1=2401 PW=9.0 AQ=1.352 RD=0.00 NS=1 SR=8.45 TE=301K 21 March 2022 Opr: Daeva E.D.; Solv: DMSO-d6;

The Best Applied NMR! On-Line: "http://nmr.ioc.ac.ru:8080"

/KANI IA1656.1

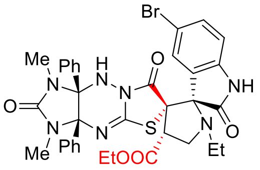

6i

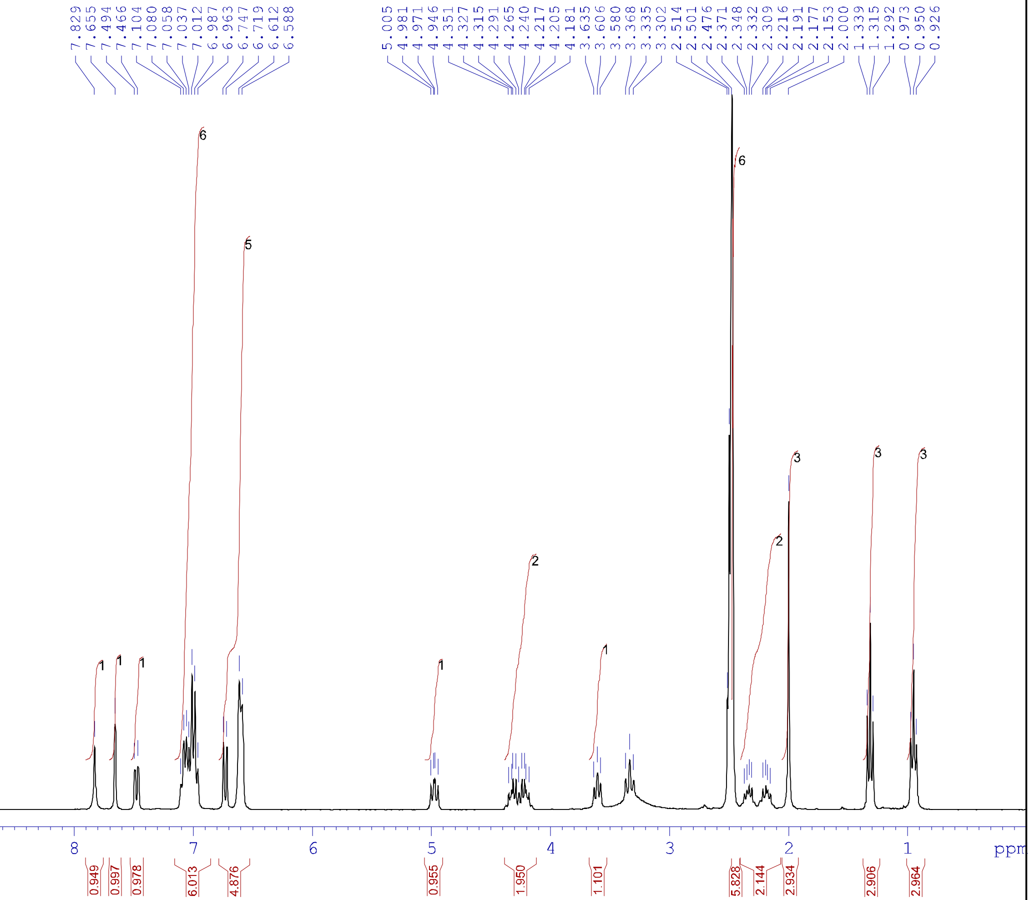

© Zelinsky Institute of Organic Chemistry, Moscow; Bruker AM300 SF=75.47 MHz {13C} SI=32K SW=18114 O1=8301 PW=14.3 AQ=0.901 RD=1.00 NS=550 SR=40.96 TE=302K 22 March 2022 Opr: Daeva E.D.; Solv: DMSO-d6;

The Best Applied NMR! On-Line: "http://nmr.ioc.ac.ru:8080"

/KANI IA1656.1

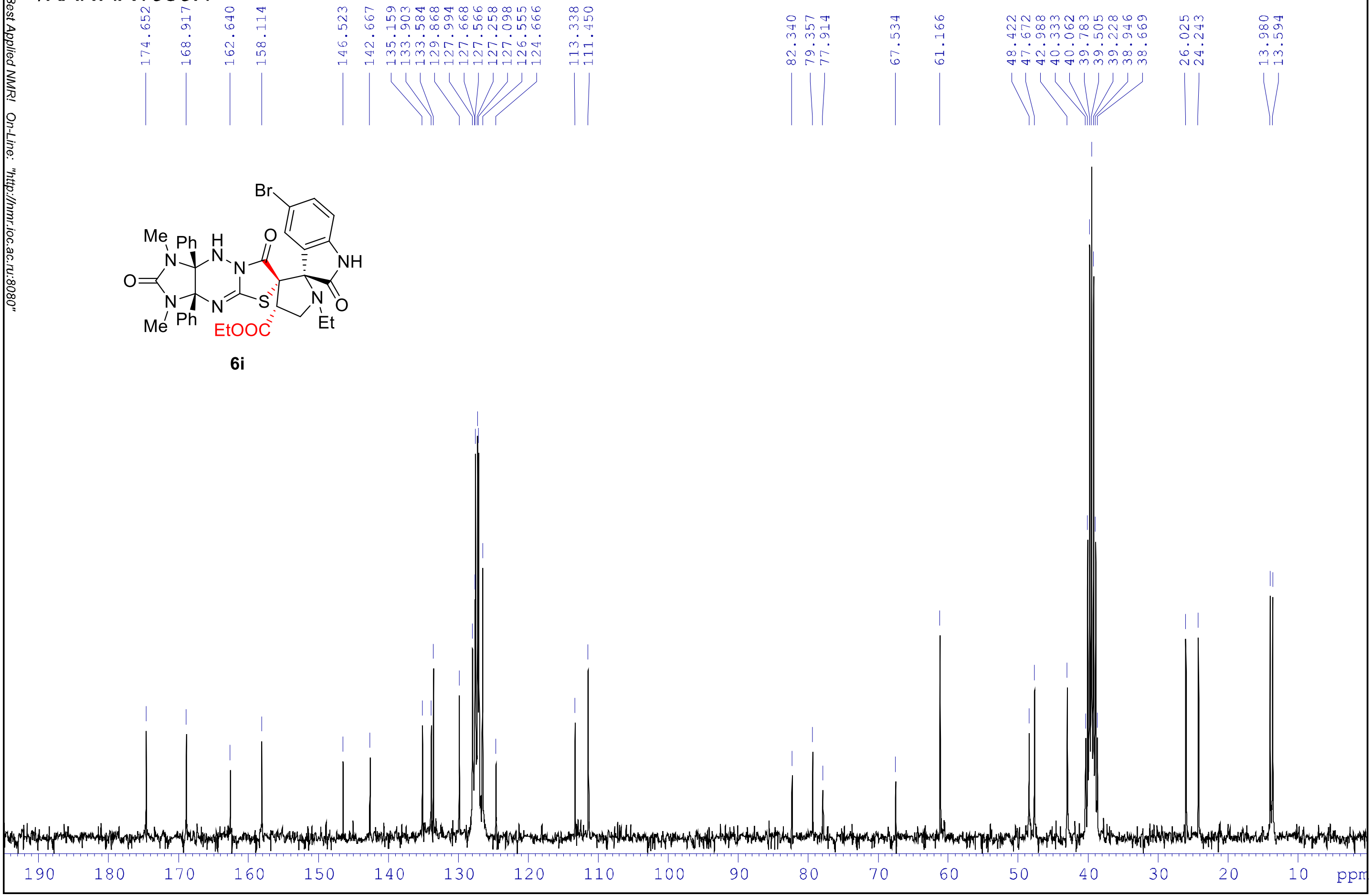

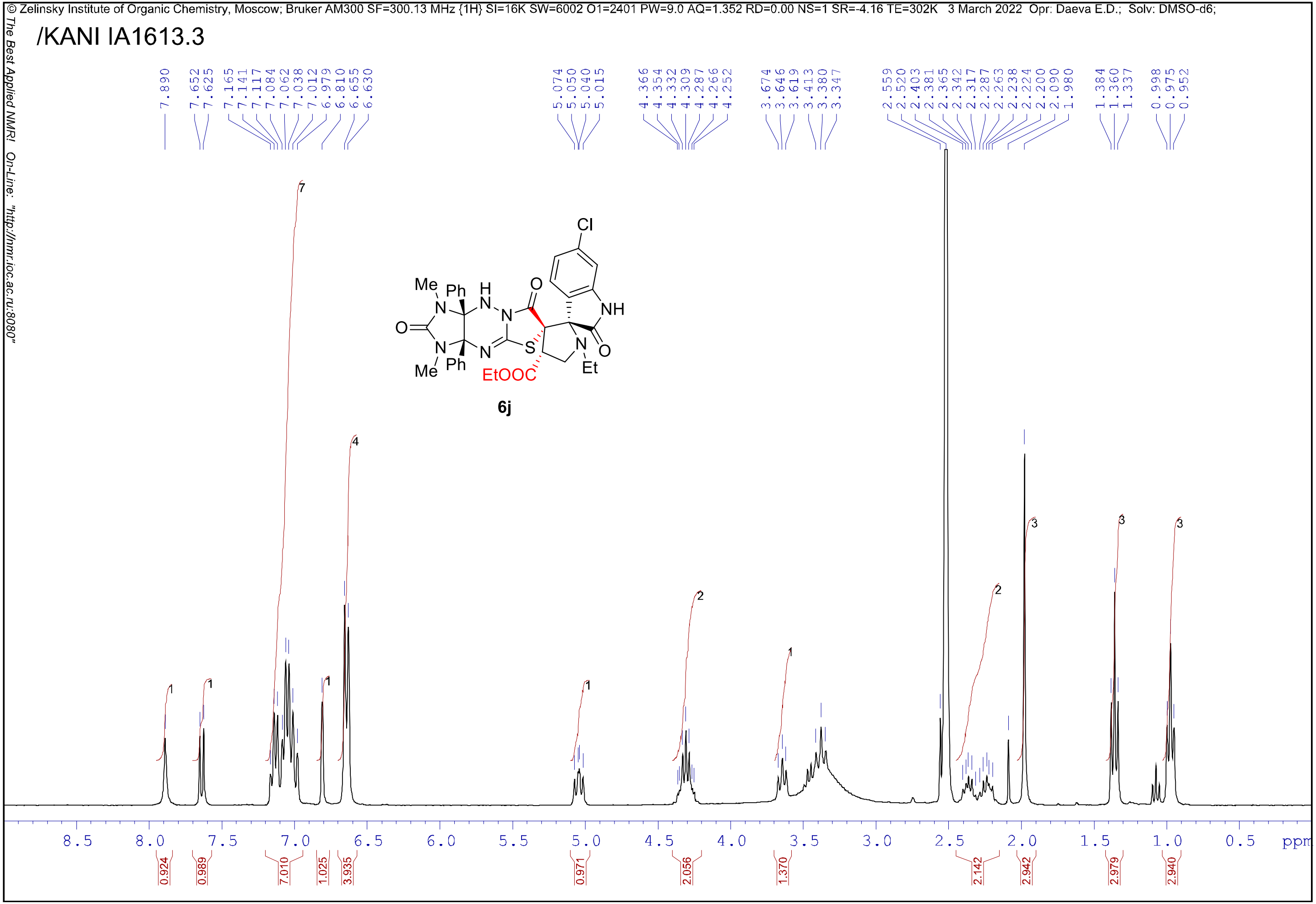
© Zelinsky Institute of Organic Chemistry, Moscow; Bruker AM300 SF=300.13 MHz {1H} SI=16K SW=6002 O1=2401 PW=9.0 AQ=1.352 RD=0.00 NS=1 SR=-4.16 TE=302K 3 March 2022 Opr: Daeva E.D.; Solv: DMSO-d6;
The Best Applied NMR! On-Line: "http://nmr.ioc.ac.ru:8080"
/KANI IA1613.3
7.890 7.652 7.625 7.165 7.141 7.117 7.084 7.062 7.038 7.012 6.979 6.810 6.655 6.630
5.074 5.050 5.040 5.015
4.366 4.354 4.332 4.309 4.287 4.266 4.252
3.674 3.646 3.619 3.413 3.380 3.347
2.559 2.520 2.403 2.381 2.365 2.342 2.317 2.287 2.263 2.238 2.224 2.200 2.090 1.980
1.384 1.360 1.337
0.998 0.975 0.952
Cl
NH
O
Me
Ph
H
N
S
EtOOC
Et
6j
ppm
8.5 8.0 7.5 7.0 6.5 6.0 5.5 5.0 4.5 4.0 3.5 3.0 2.5 2.0 1.5 1.0 0.5
0.924 0.989 7.010 1.025 3.935 0.971 2.056 1.370 2.142 2.942 2.979 2.940

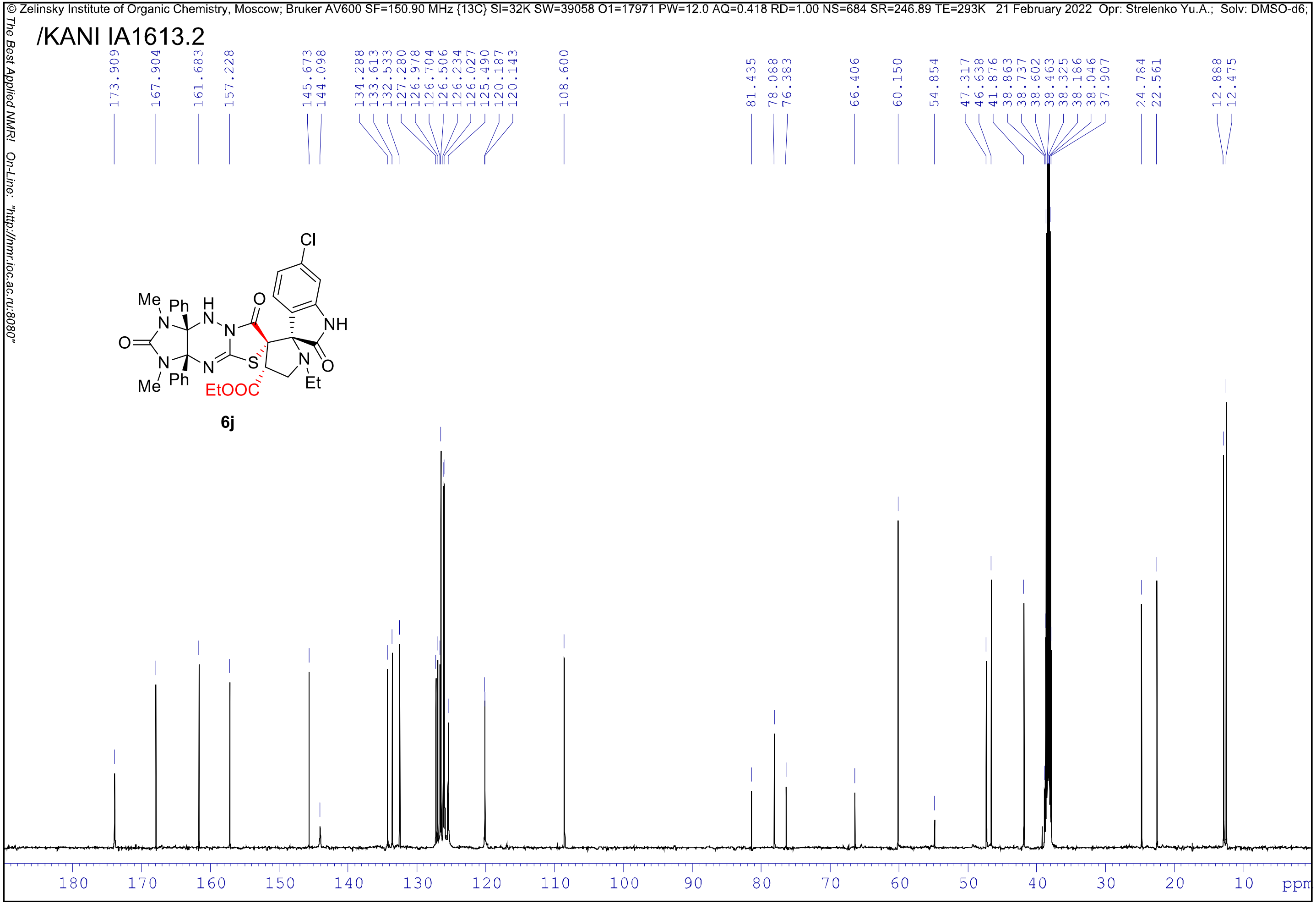
© Zelinsky Institute of Organic Chemistry, Moscow; Bruker AV600 SF=150.90 MHz {13C} SI=32K SW=39058 O1=17971 PW=12.0 AQ=0.418 RD=1.00 NS=684 SR=246.89 TE=293K 21 February 2022 Opr: Strelenko Yu.A.; Solv: DMSO-d6;
The Best Applied NMR! On-Line: "http://nmr.ioc.ac.ru:8080"
/KANI IA1613.2
173.909
167.904
161.683
157.228
145.673
144.098
134.288
133.613
132.533
127.280
126.978
126.704
126.506
126.234
126.027
125.490
120.187
120.143
108.600
81.435
78.088
76.383
66.406
60.150
54.854
47.317
46.638
41.876
38.863
38.737
38.602
38.463
38.325
38.186
38.046
37.907
24.784
22.561
12.888
12.475
Cl
Me
Ph
H
N
O
NH
O
N
S
N
O
N
Ph
Me
EtOOC
Et
6j
180 170 160 150 140 130 120 110 100 90 80 70 60 50 40 30 20 10 ppm

© Zelinsky Institute of Organic Chemistr y, Moscow; Bruker AM300 SF=300.13 MHz {1H} SI=32K SW=6002 O1=2401 PW=9.0 AQ=1.352 RD=0.00 NS=1 SR=1.70 TE=301K 25 January 2021 Opr: Daeva E.D.; Solv: DMSO-d6;

/KANI VDB108.1

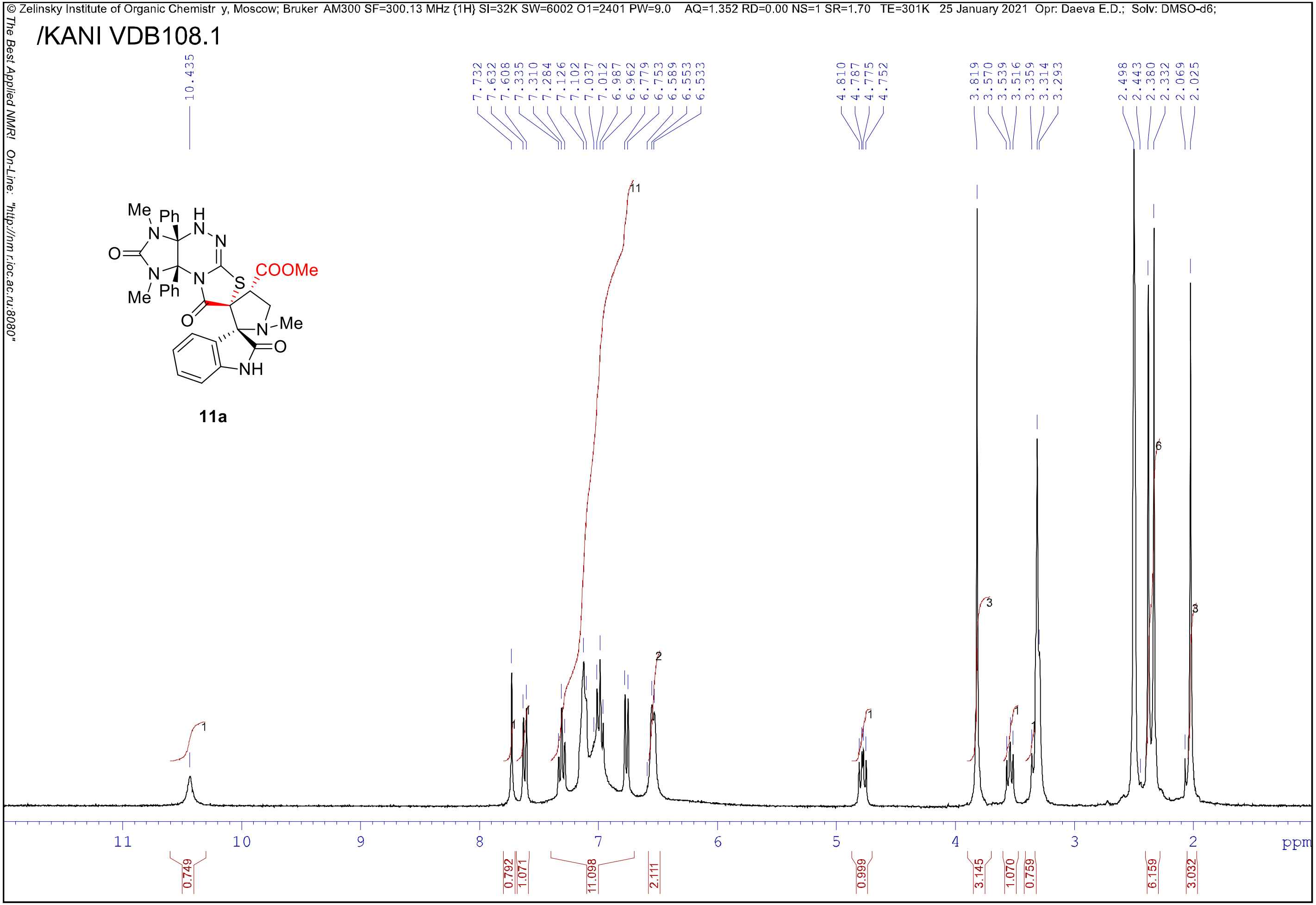

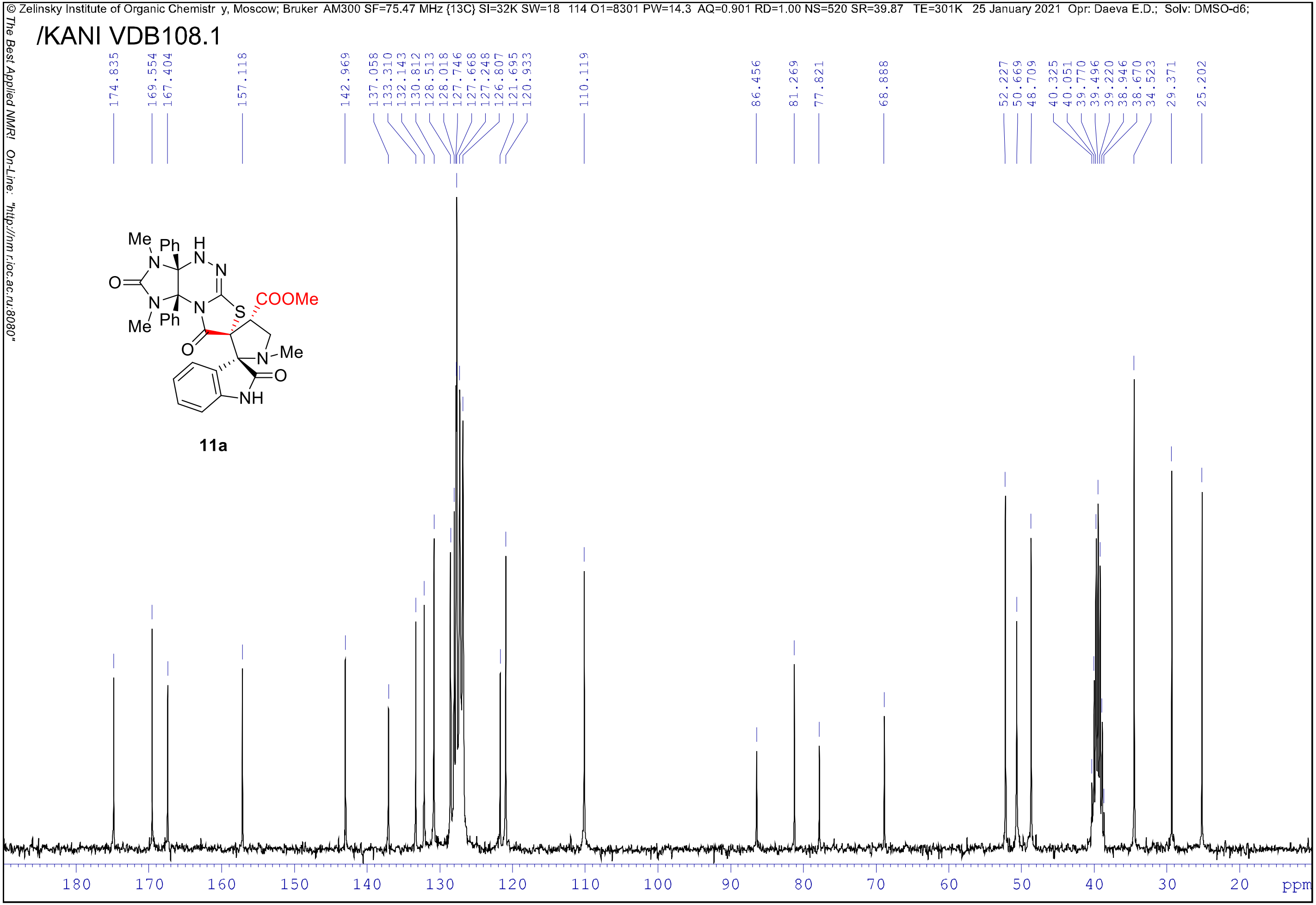
© Zelinsky Institute of Organic Chemistr y, Moscow; Bruker AM300 SF=75.47 MHz {13C} SI=32K SW=18 114 O1=8301 PW=14.3 AQ=0.901 RD=1.00 NS=520 SR=39.87 TE=301K 25 January 2021 Opr: Daeva E.D.; Solv: DMSO-d6;
/KANI VDB108.1
The Best Applied NMR! On-Line: "http://nm r.ioc.ac.ru:8080"
174.835
169.554
167.404
157.118
142.969
137.058
133.310
132.143
130.812
128.513
128.018
127.746
127.668
127.248
126.807
121.695
120.933
110.119
86.456
81.269
77.821
68.888
52.227
50.669
48.709
40.325
40.051
39.770
39.496
39.220
38.946
38.670
34.523
29.371
25.202
Me
Ph
H
N
N
N
O
COOMe
N
S
N
Me
Ph
N
O
N-Me
O
NH
11a
180
170
160
150
140
130
120
110
100
90
80
70
60
50
40
30
20
ppm

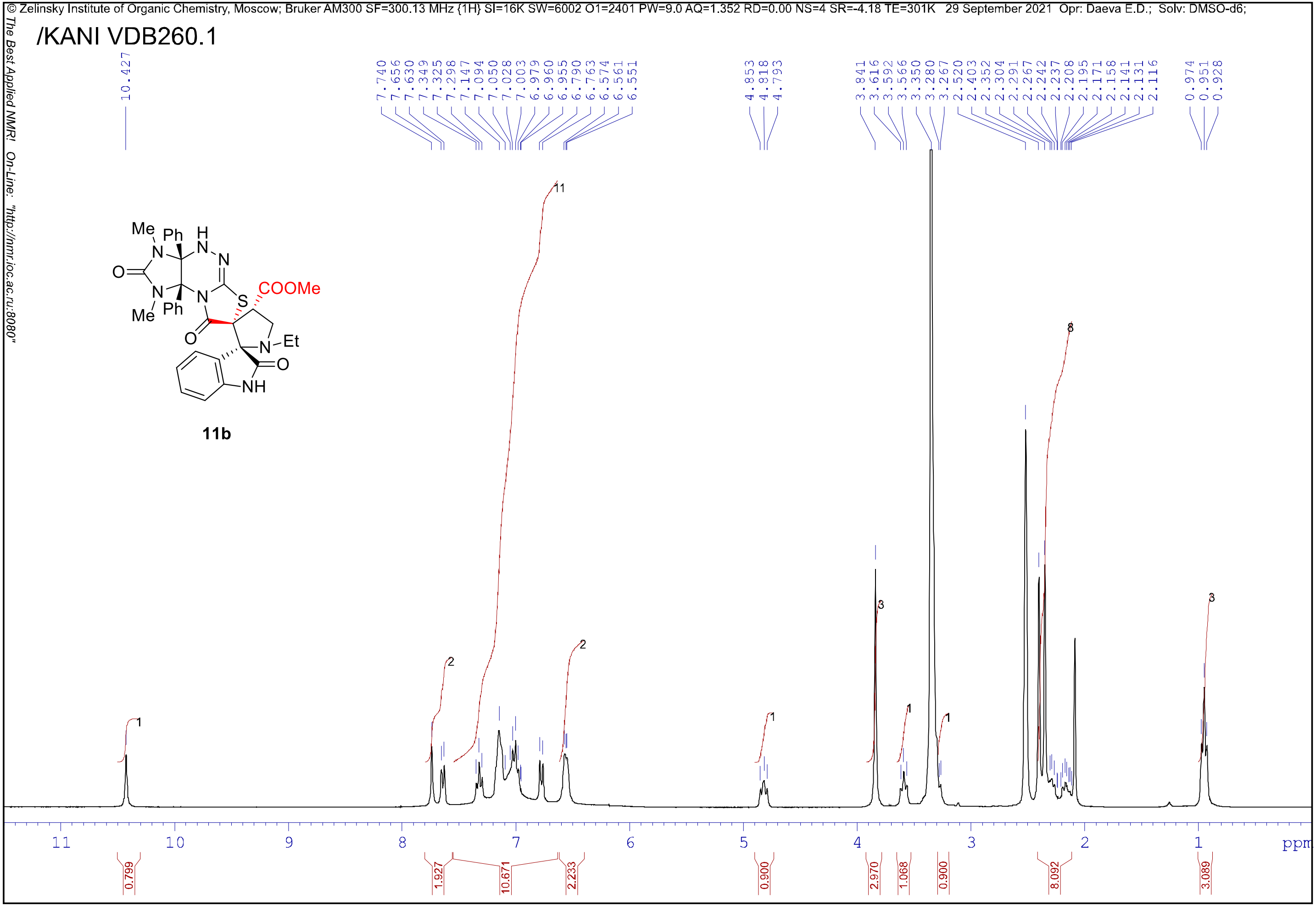
© Zelinsky Institute of Organic Chemistry, Moscow; Bruker AM300 SF=300.13 MHz {1H} SI=16K SW=6002 O1=2401 PW=9.0 AQ=1.352 RD=0.00 NS=4 SR=-4.18 TE=301K 29 September 2021 Opr: Daeva E.D.; Solv: DMSO-d6;
The Best Applied NMR! On-Line: "http://nmr.ioc.ac.ru:8080"
/KANI VDB260.1
10.427
7.740 7.656 7.630 7.349 7.325 7.298 7.147 7.094 7.050 7.028 7.003 6.979 6.960 6.955 6.790 6.763 6.574 6.561 6.551
4.853 4.818 4.793
3.841 3.616 3.592 3.566 3.350 3.280 3.267 2.520 2.403 2.352 2.304 2.291 2.267 2.242 2.237 2.208 2.195 2.171 2.158 2.141 2.131 2.116
0.974 0.951 0.928
Me
Ph
H
N
N
N
O
COOMe
S
Ph
Me
Et
NH
11b
ppm
0.799
1.927
10.671
2.233
0.900
2.970
1.068
0.900
8.092
3.089

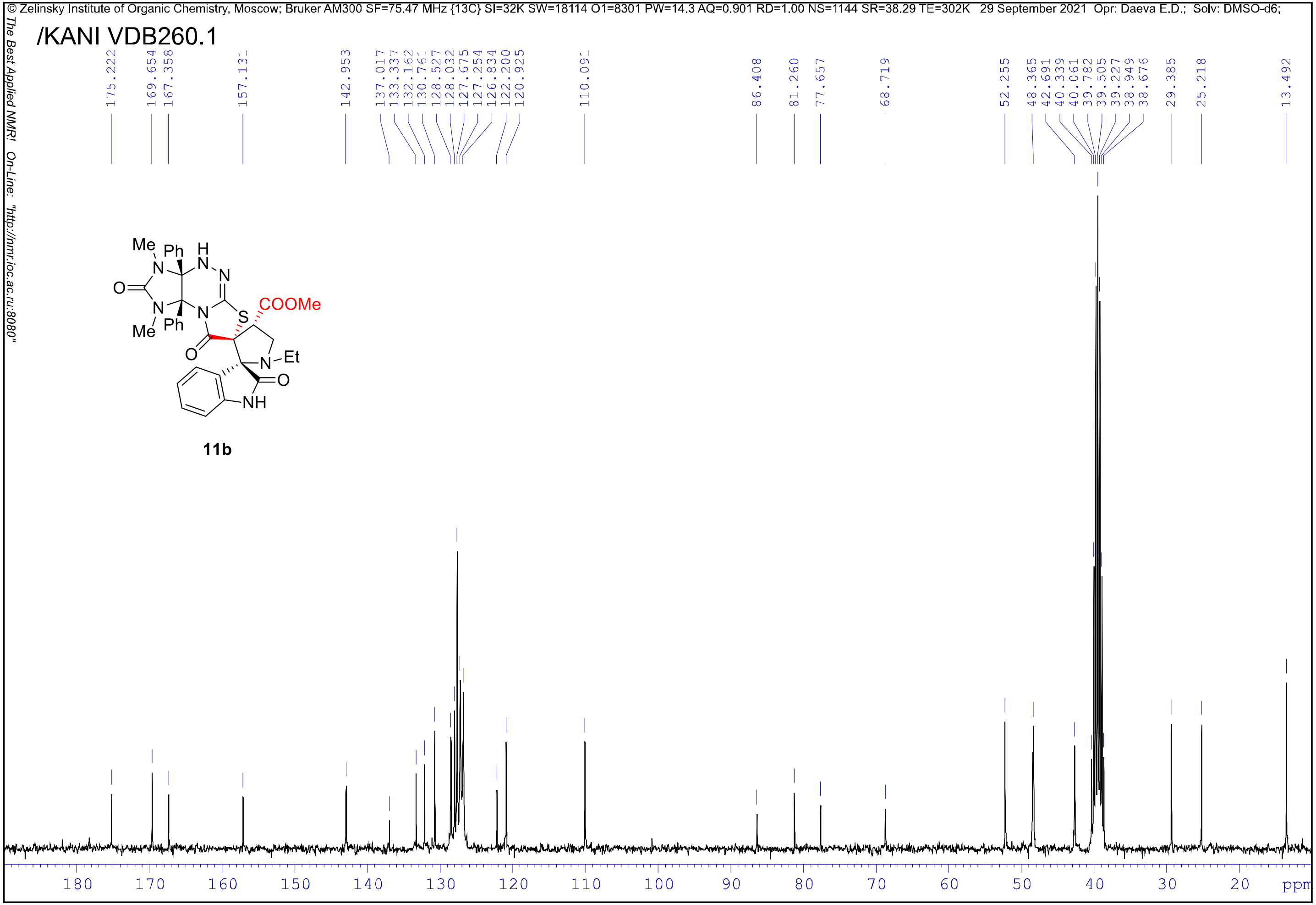
© Zelinsky Institute of Organic Chemistry, Moscow; Bruker AM300 SF=75.47 MHz {13C} SI=32K SW=18114 O1=8301 PW=14.3 AQ=0.901 RD=1.00 NS=1144 SR=38.29 TE=302K 29 September 2021 Opr: Daeva E.D.; Solv: DMSO-d6;
The Best Applied NMR! On-Line: "http://nmr.ioc.ac.ru:8080"
/KANI VDB260.1
175.222
169.654
167.358
157.131
142.953
137.017
133.337
132.162
130.761
128.527
128.032
127.675
127.254
126.834
122.200
120.925
110.091
86.408
81.260
77.657
68.719
52.255
48.365
42.691
40.339
40.061
39.782
39.505
39.227
38.949
38.676
29.385
25.218
13.492
Me
Ph
H
N
N
N
O
N
N
S
COOMe
Me
Ph
O
N
Et
O
NH
11b
180
170
160
150
140
130
120
110
100
90
80
70
60
50
40
30
20
ppm

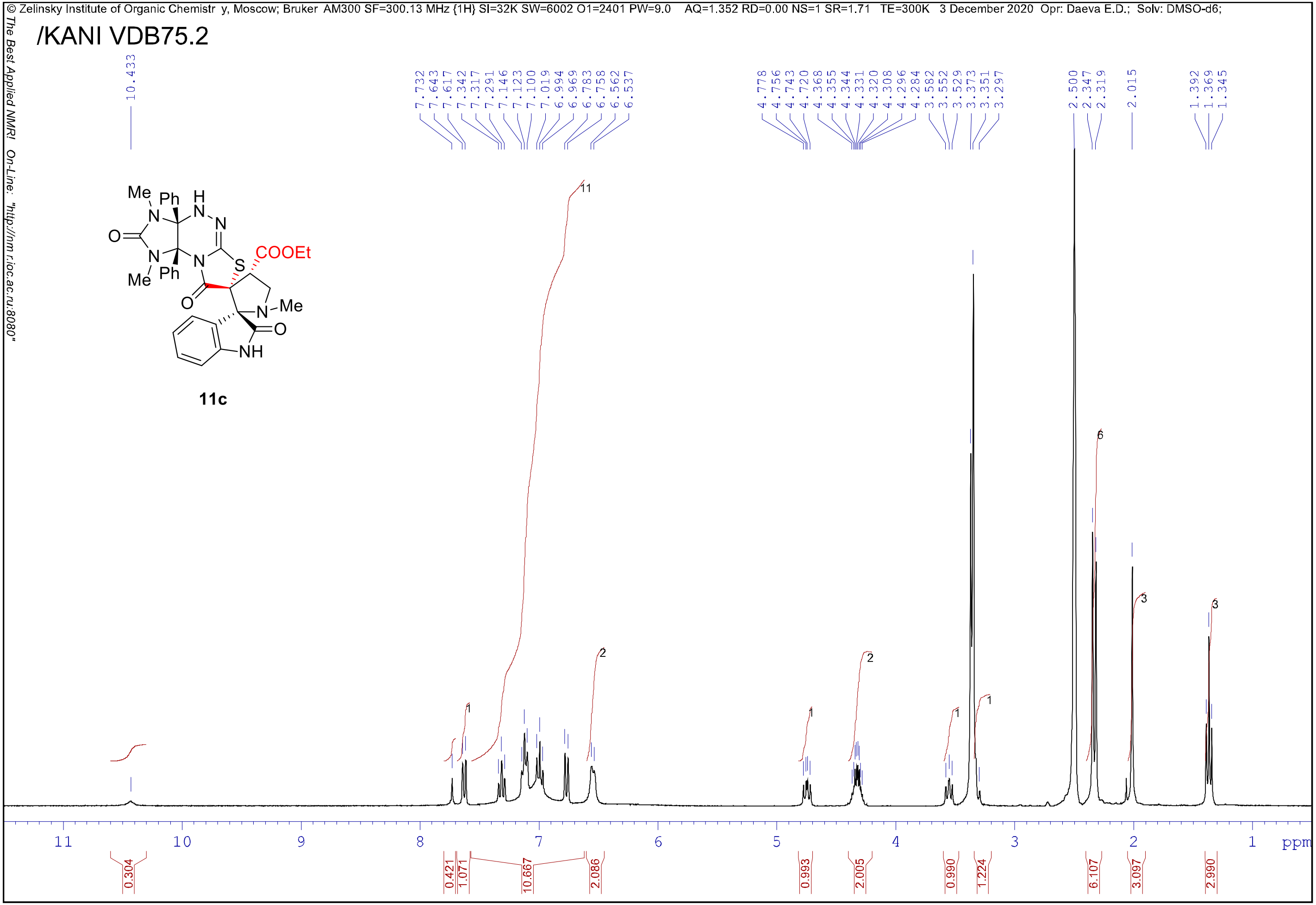
© Zelinsky Institute of Organic Chemistr y, Moscow; Bruker AM300 SF=300.13 MHz {1H} SI=32K SW=6002 O1=2401 PW=9.0 AQ=1.352 RD=0.00 NS=1 SR=1.71 TE=300K 3 December 2020 Opr: Daeva E.D.; Solv: DMSO-d6;
The Best Applied NMR! On-Line: "http://nmr.ioc.ac.ru:8080"
/KANI VDB75.2
10.433
7.732 7.643 7.617 7.342 7.317 7.291 7.146 7.123 7.100 7.019 6.994 6.969 6.783 6.758 6.562 6.537
4.778 4.756 4.743 4.720 4.368 4.355 4.344 4.331 4.320 4.308 4.296 4.284 3.582 3.552 3.529 3.373 3.351 3.297
2.500 2.347 2.319 2.015 1.392 1.369 1.345
Me
Ph
H
N
N
N
O
COOEt
S
Me
NH
11c
0.304 0.421 1.071 10.667 2.086 0.993 2.005 0.990 1.224 6.107 3.097 2.990
ppm

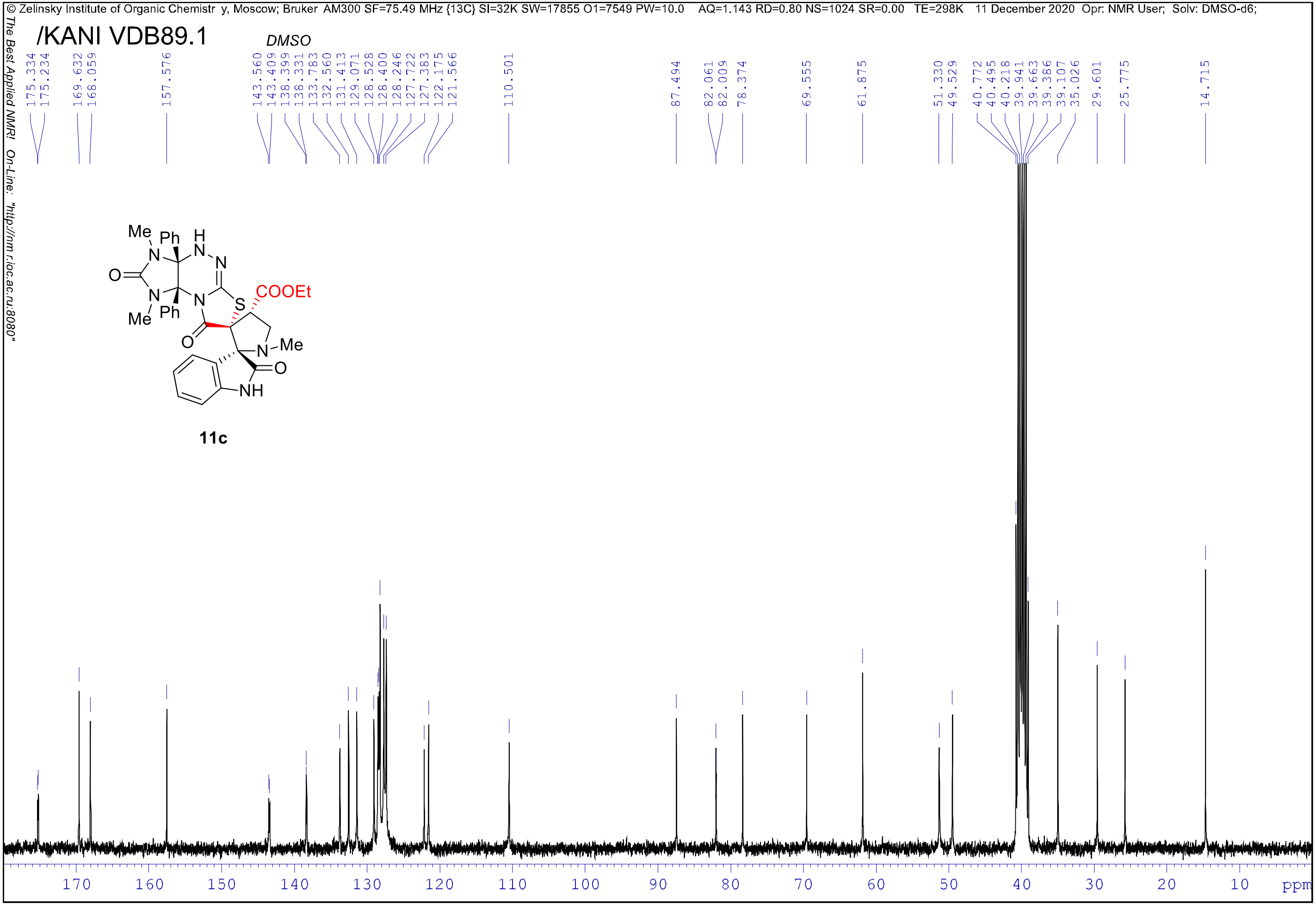
© Zelinsky Institute of Organic Chemistr y, Moscow; Bruker AM300 SF=75.49 MHz {13C} SI=32K SW=17855 O1=7549 PW=10.0 AQ=1.143 RD=0.80 NS=1024 SR=0.00 TE=298K 11 December 2020 Opr: NMR User; Solv: DMSO-d6;
The Best Applied NMR! On-Line: "http://nm r.ioc.ac.ru:8080"
/KANI VDB89.1
DMSO
175.334 175.234 169.632 168.059 157.576 143.560 143.409 138.399 138.331 133.783 132.560 131.413 129.071 128.528 128.400 128.246 127.722 127.383 122.175 121.566 110.501 87.494 82.061 82.009 78.374 69.555 61.875 51.330 49.529 40.772 40.495 40.218 39.941 39.663 39.386 39.107 35.026 29.601 25.775 14.715
Me
Ph
H
N
N
N
O
N
N
S
COOEt
Me
Ph
O
N
Me
O
NH
11c
170 160 150 140 130 120 110 100 90 80 70 60 50 40 30 20 10 ppm

© Zelinsky Institute of Organic Chemistry, Moscow; Bruker AV300 SF=300.13 MHz {1H} SI=64K SW=6010 O1=2101 PW=15.0 AQ=2.715 RD=0.10 NS=1 SR=0.00 TE=300K 12 August 2021 Opr: NMR User; Solv: DMSO-d6;

The Best Applied NMR! On-Line: "http://nmr.ioc.ac.ru:8080"

/KANI VDB221.1

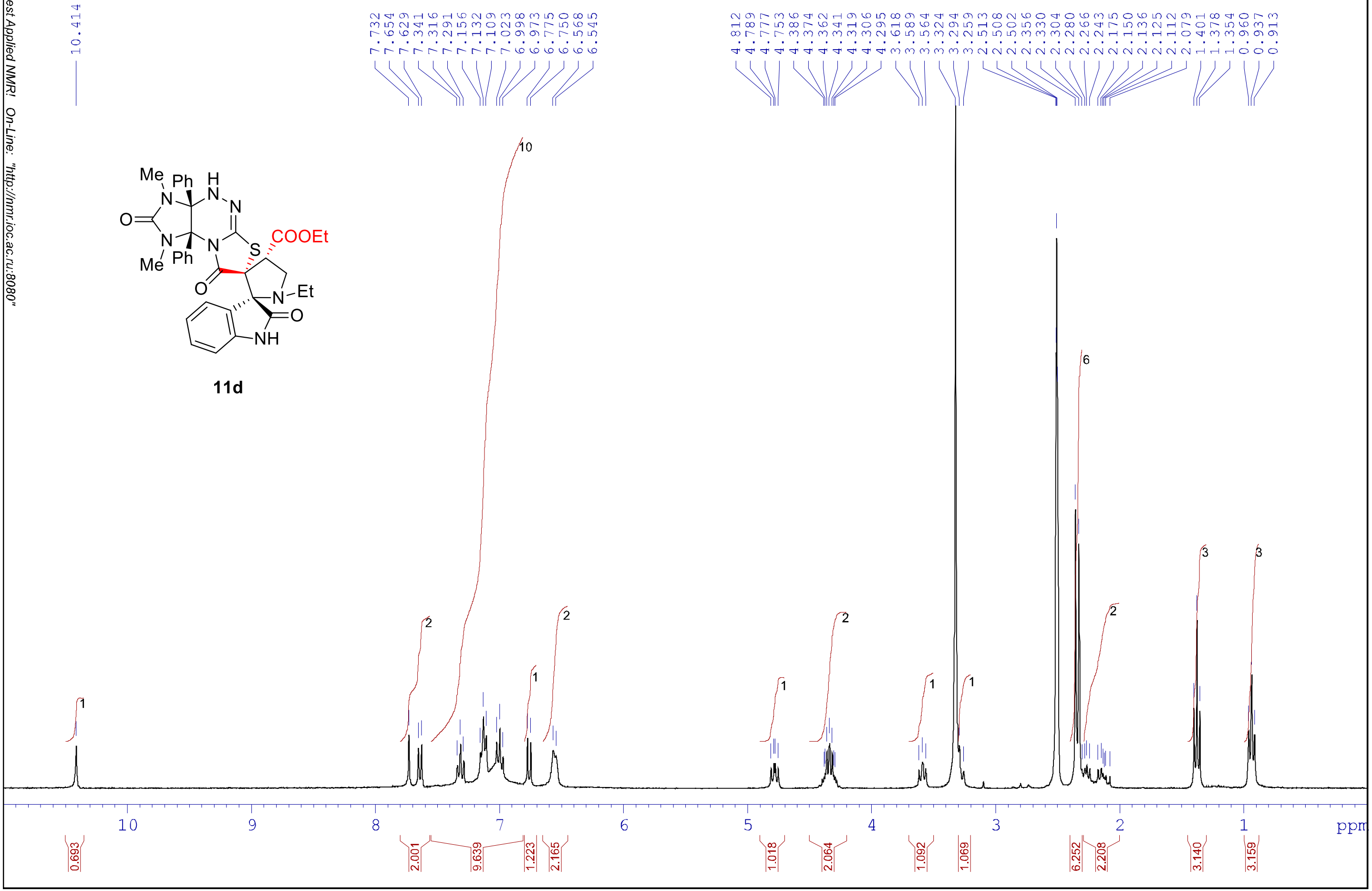

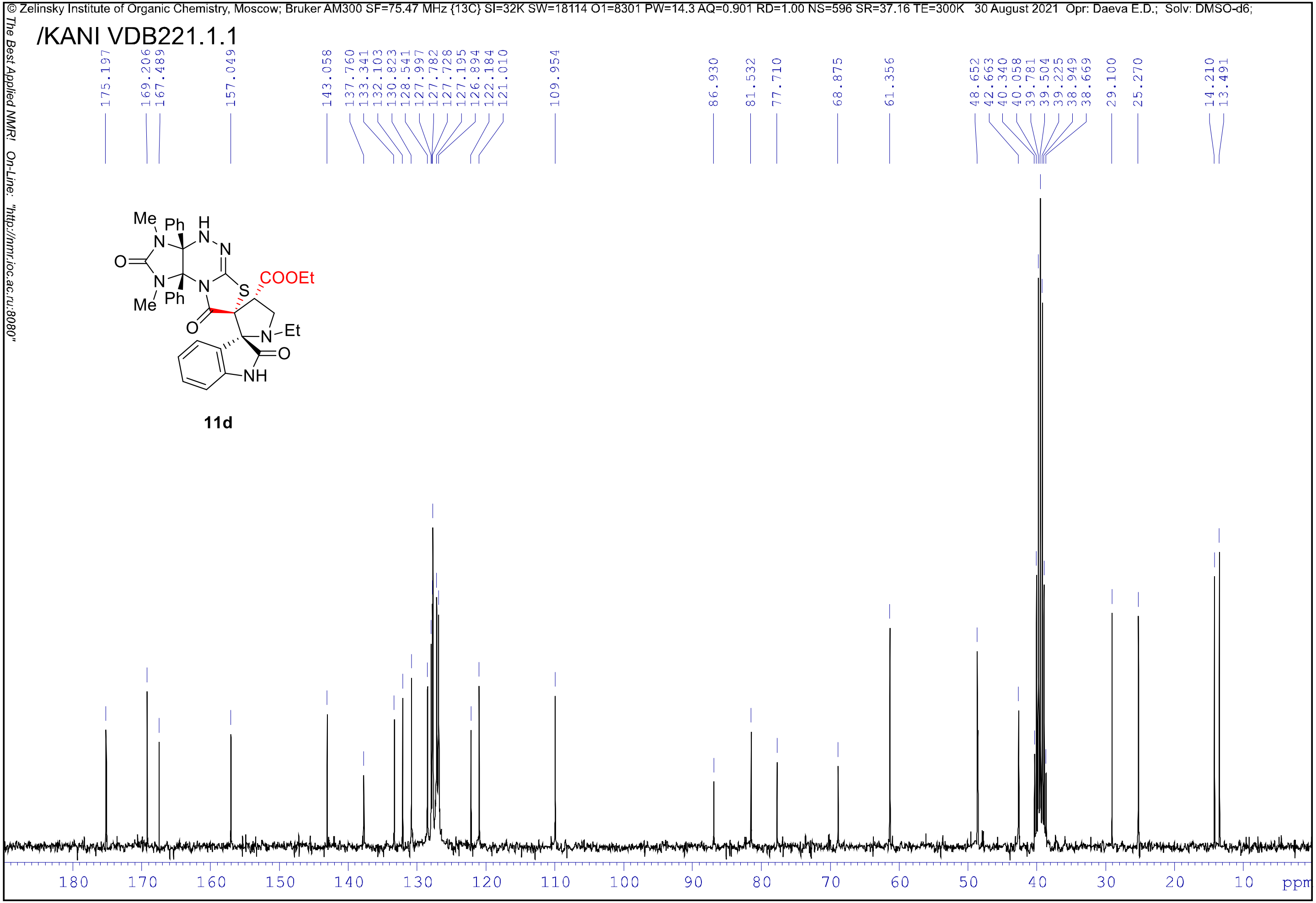
© Zelinsky Institute of Organic Chemistry, Moscow; Bruker AM300 SF=75.47 MHz {13C} SI=32K SW=18114 O1=8301 PW=14.3 AQ=0.901 RD=1.00 NS=596 SR=37.16 TE=300K 30 August 2021 Opr: Daeva E.D.; Solv: DMSO-d6;
/KANI VDB221.1.1
The Best Applied NMR! On-Line: "http://nmr.ioc.ac.ru:8080"
175.197
169.206
167.489
157.049
143.058
137.760
133.341
132.103
130.823
128.541
127.997
127.782
127.728
127.195
126.894
122.184
121.010
109.954
86.930
81.532
77.710
68.875
61.356
48.652
42.663
40.340
40.058
39.781
39.504
39.225
38.949
38.669
29.100
25.270
14.210
13.491
Me
Ph
H
N
N
N
O
N
N
Me
Ph
S
COOEt
O
N
Et
O
NH
180
170
160
150
140
130
120
110
100
90
80
70
60
50
40
30
20
10
ppm

11d

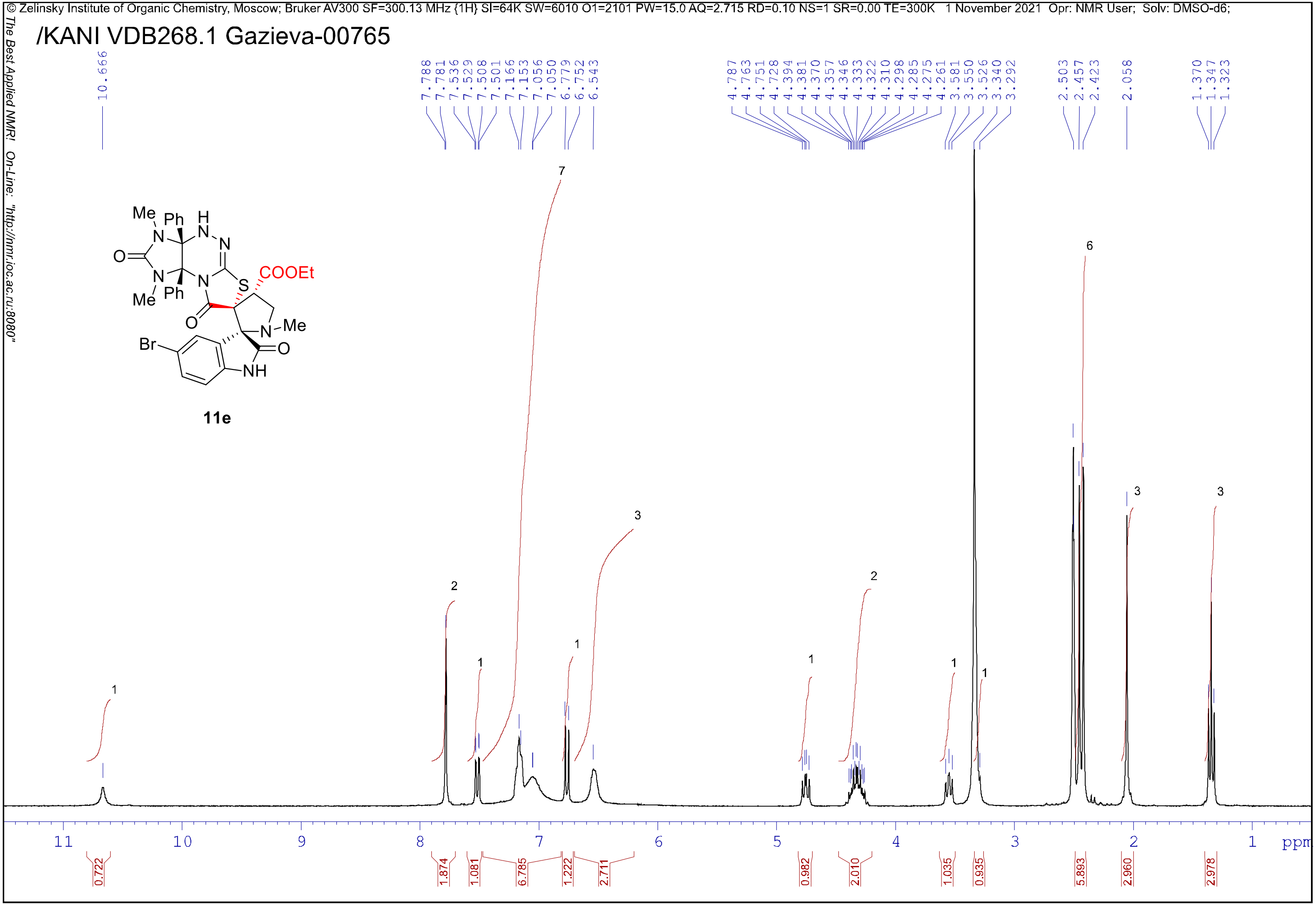
© Zelinsky Institute of Organic Chemistry, Moscow; Bruker AV300 SF=300.13 MHz {1H} SI=64K SW=6010 O1=2101 PW=15.0 AQ=2.715 RD=0.10 NS=1 SR=0.00 TE=300K 1 November 2021 Opr: NMR User; Solv: DMSO-d6;
/KANI VDB268.1 Gazieva-00765
The Best Applied NMR! On-Line: "http://nmr.ioc.ac.ru:8080"
10.666
7.788 7.781 7.536 7.529 7.508 7.501 7.166 7.153 7.056 7.050 6.779 6.752 6.543
4.787 4.763 4.751 4.728 4.394 4.381 4.370 4.357 4.346 4.333 4.322 4.310 4.298 4.285 4.275 4.261 3.581 3.550 3.526 3.340 3.292
2.503 2.457 2.423 2.058 1.370 1.347 1.323
Me Ph H N N N O N N S COOEt N Me Ph O N Me Br NH O
11e
11 10 9 8 7 6 5 4 3 2 1 ppm
0.722 1.874 1.081 6.785 1.222 2.711 0.982 2.010 1.035 0.935 5.893 2.960 2.978

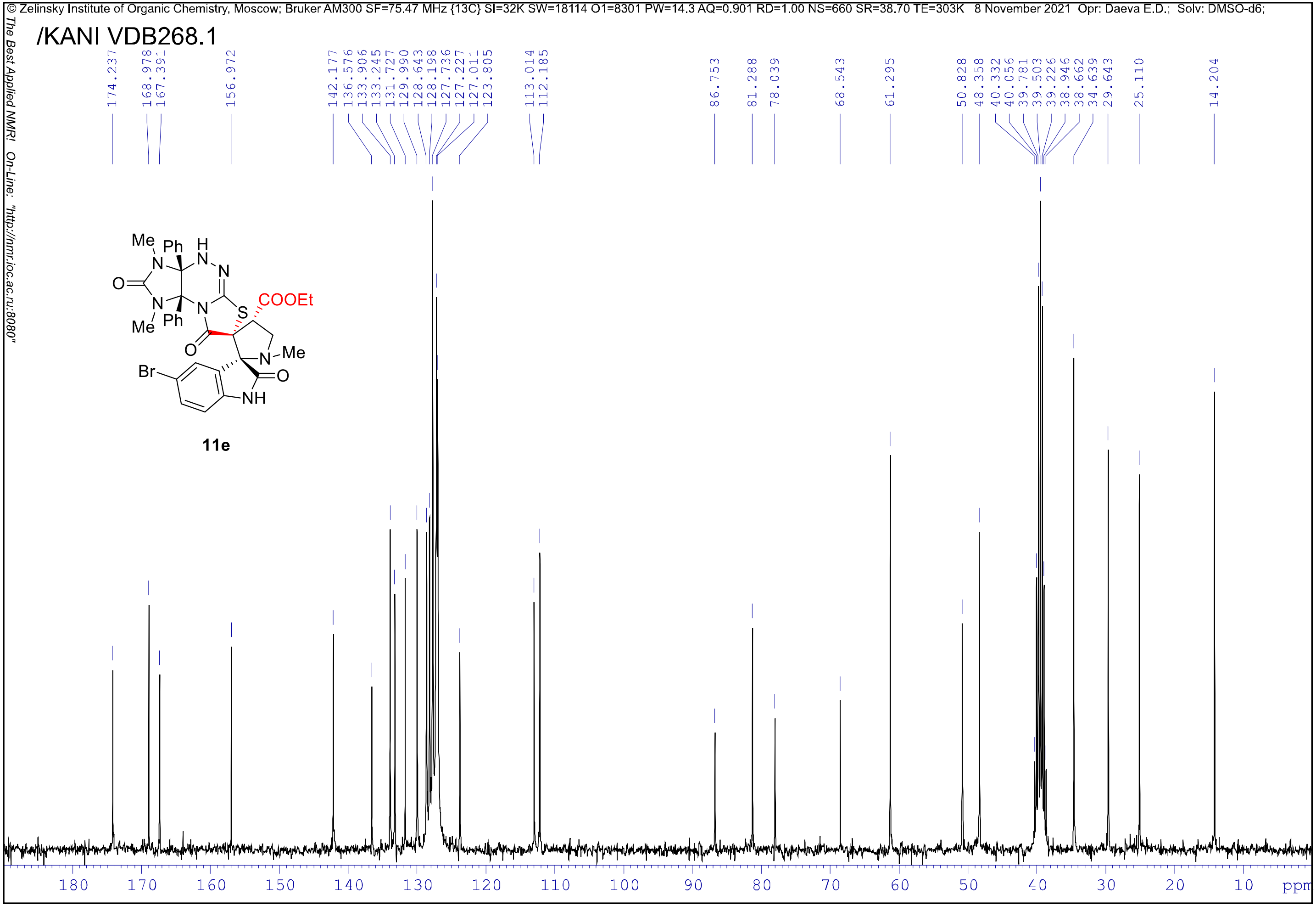
© Zelinsky Institute of Organic Chemistry, Moscow; Bruker AM300 SF=75.47 MHz {13C} SI=32K SW=18114 O1=8301 PW=14.3 AQ=0.901 RD=1.00 NS=660 SR=38.70 TE=303K 8 November 2021 Opr: Daeva E.D.; Solv: DMSO-d6;
The Best Applied NMR! On-Line: "http://nmr.ioc.ac.ru:8080"
/KANI VDB268.1
174.237
168.978
167.391
156.972
142.177
136.576
133.906
133.245
131.727
129.990
128.643
128.198
127.736
127.227
127.011
123.805
113.014
112.185
86.753
81.288
78.039
68.543
61.295
50.828
48.358
40.332
40.056
39.781
39.503
39.226
38.946
38.662
34.639
29.643
25.110
14.204
Me
Ph
H
N
N
N
O
N
N
S
COOEt
Me
Ph
O
N
Me
Br
O
NH
11e
180
170
160
150
140
130
120
110
100
90
80
70
60
50
40
30
20
10
ppm

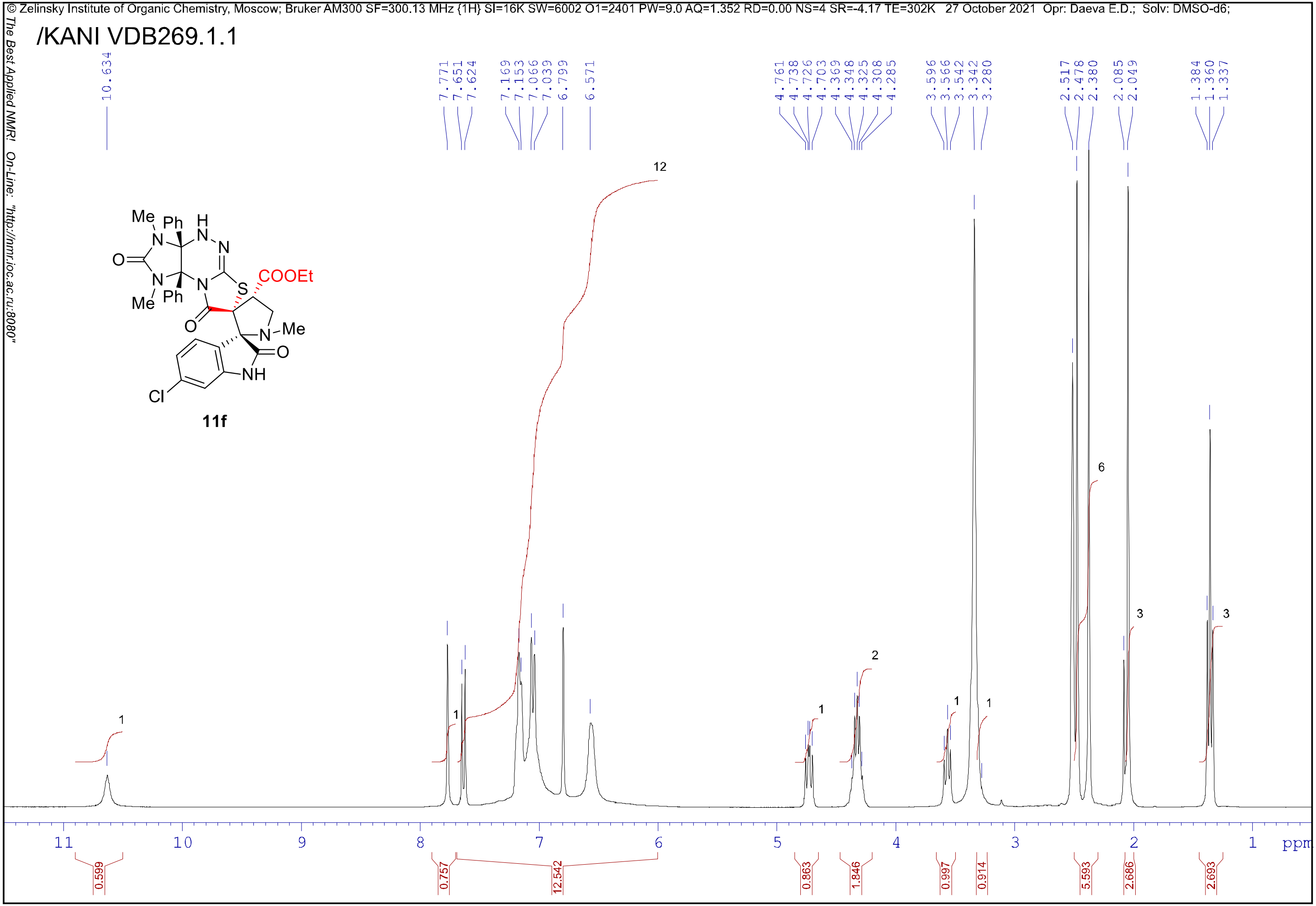
© Zelinsky Institute of Organic Chemistry, Moscow; Bruker AM300 SF=300.13 MHz {1H} SI=16K SW=6002 O1=2401 PW=9.0 AQ=1.352 RD=0.00 NS=4 SR=-4.17 TE=302K 27 October 2021 Opr: Daeva E.D.; Solv: DMSO-d6;
/KANI VDB269.1.1
The Best Applied NMR! On-Line: "http://nmr.ioc.ac.ru:8080"
10.634
7.771 7.651 7.624
7.169 7.153 7.066 7.039 6.799
6.571
4.761 4.738 4.726 4.703 4.369 4.348 4.325 4.308 4.285
3.596 3.566 3.542 3.342 3.280
2.517 2.478 2.380 2.085 2.049
1.384 1.360 1.337
COOEt
11f
0.599 0.757 12.542 0.863 1.846 0.997 0.914 5.593 2.686 2.693
ppm

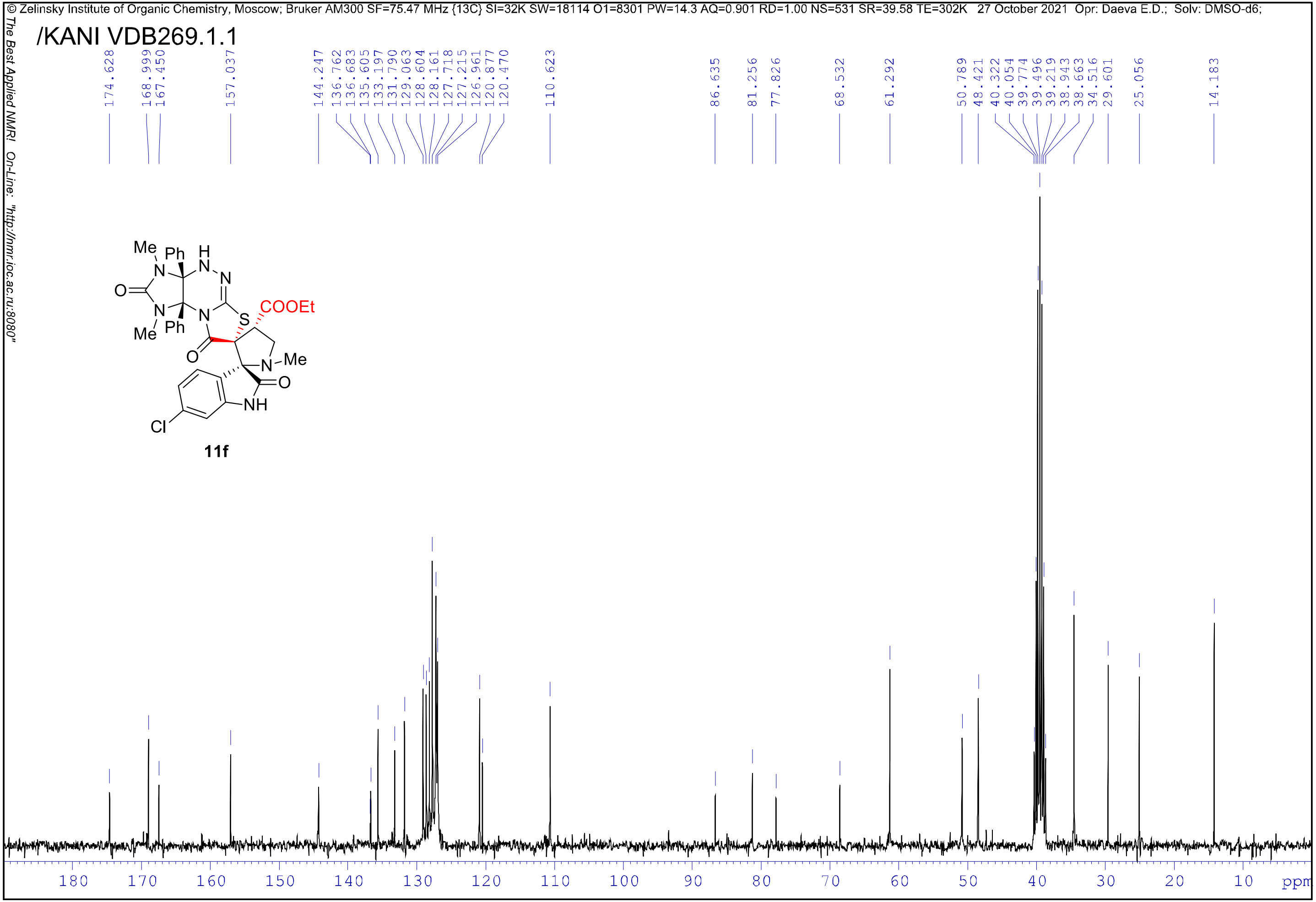
© Zelinsky Institute of Organic Chemistry, Moscow; Bruker AM300 SF=75.47 MHz {13C} SI=32K SW=18114 O1=8301 PW=14.3 AQ=0.901 RD=1.00 NS=531 SR=39.58 TE=302K 27 October 2021 Opr: Daeva E.D.; Solv: DMSO-d6;
/KANI VDB269.1.1
The Best Applied NMR! On-Line: "http://nmr.ioc.ac.ru:8080"
174.628
168.999
167.450
157.037
144.247
136.762
136.683
135.605
133.197
131.790
129.063
128.604
128.161
127.718
127.215
126.961
120.877
120.470
110.623
86.635
81.256
77.826
68.532
61.292
50.789
48.421
40.322
40.054
39.774
39.496
39.219
38.943
38.663
34.516
29.601
25.056
14.183
COOEt
11f
180
170
160
150
140
130
120
110
100
90
80
70
60
50
40
30
20
10
ppm

© Zelinsky Institute of Organic Chemistry, Moscow; Bruker DRX500 SF=500.13 MHz {1H-1H}NOESY SI=1024x256 SW=5631 O1=3099 PW=8.2 AQ=0.078 RD=1.00 NS=4x128 SR=6.18 TE=299K 13 January 2022 Opr: Strelenko Yu.A.; Solv: DMSO-d6;

The Best Applied NMR! On-Line: "http://nmr.ioc.ac.ru:8080"

/KANI IA1595.3
NOESY

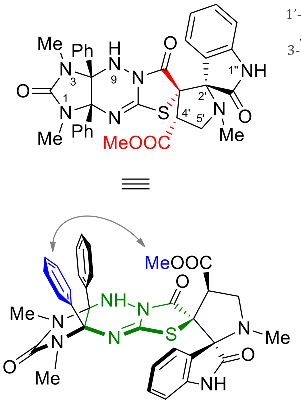

6a

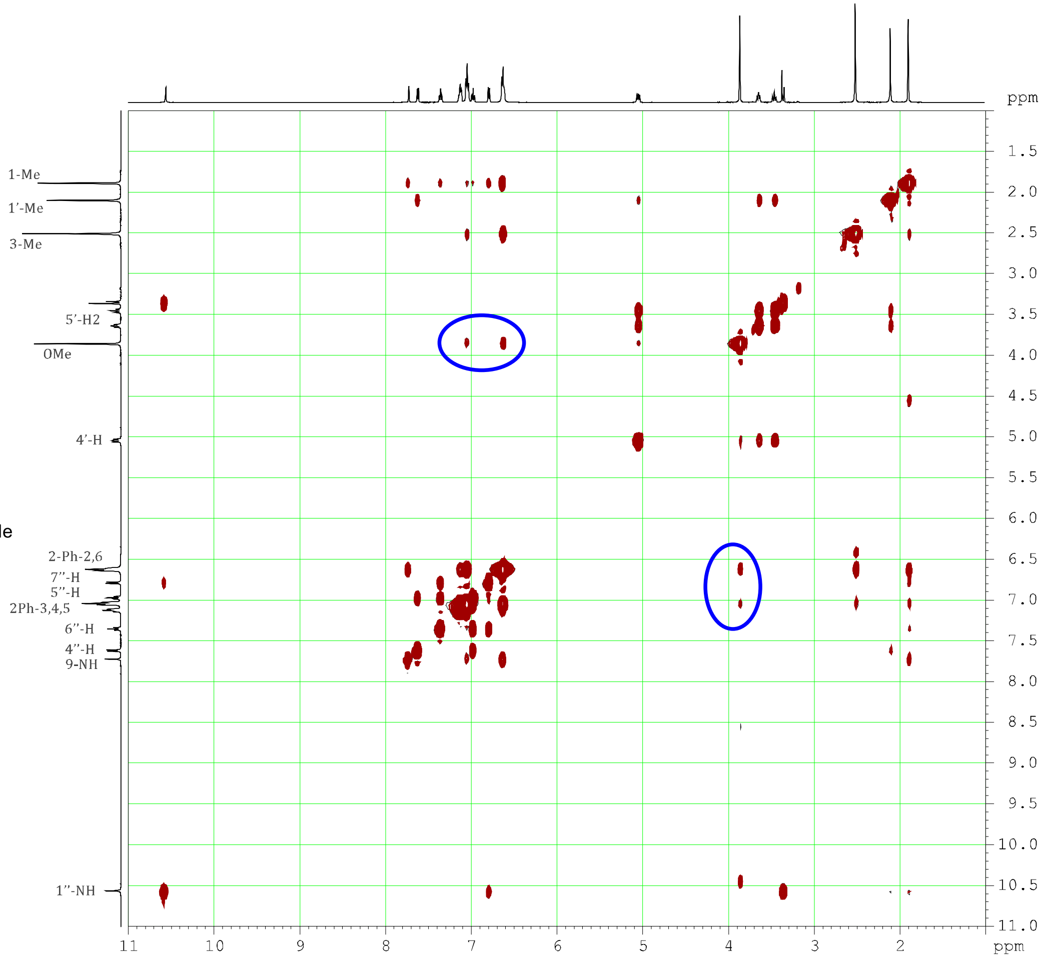

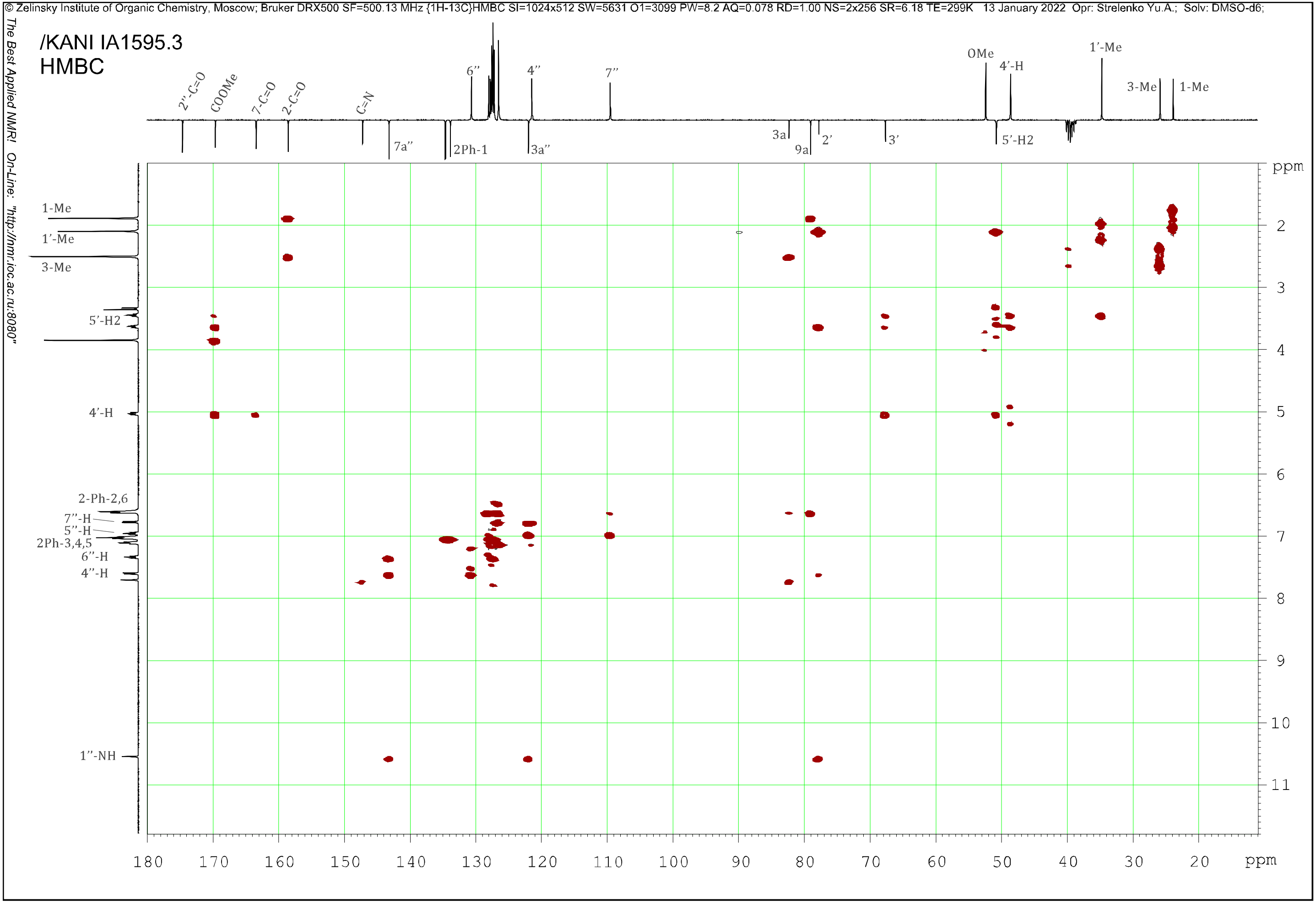
© Zelinsky Institute of Organic Chemistry, Moscow; Bruker DRX500 SF=500.13 MHz {1H-13C}HMBC SI=1024x512 SW=5631 O1=3099 PW=8.2 AQ=0.078 RD=1.00 NS=2x256 SR=6.18 TE=299K 13 January 2022 Opr: Strelenko Yu.A.; Solv: DMSO-d6;
The Best Applied NMR! On-Line: "http://nmr.ioc.ac.ru:8080"
/KANI IA1595.3
HMBC
2''-C=O
COOMe
7-C=O
2-C=O
C=N
7a''
2Ph-1
6''
4''
3a''
7''
3a
9a
2'
3'
OMe
4'-H
5'-H2
1'-Me
3-Me
1-Me
1-Me
1'-Me
3-Me
5'-H2
4'-H
2-Ph-2,6
7''-H
5''-H
2Ph-3,4,5
6''-H
4''-H
1''-NH
ppm
180 170 160 150 140 130 120 110 100 90 80 70 60 50 40 30 20 ppm
2 3 4 5 6 7 8 9 10 11

Data File D:\DATA\221003\E 60-70.D
Sample Name: E 60-70

===

Acq. Operator : Seq. Line : 1
Acq. Instrument : Instrument 1 Location : IA-1670
Injection Date : 10/3/2022 5:08:13 PM Inj : 1
Inj Volume : 5 µl

Acq. Method : D:\METHODS\CHROM_60_70.M
Last changed : 10/3/2022 5:07:17 PM
Analysis Method : D:\METHODS\CHROM_ADC_PN.m
Last changed : 10/4/2022 12:01:46 PM
Sample Info : Column: Reprosil-Pur Basic C18 250*4.6 mm 5um
A: H2O 0,01% tfa B: MeCN 0,01% tfa
5-100 %B at 20min

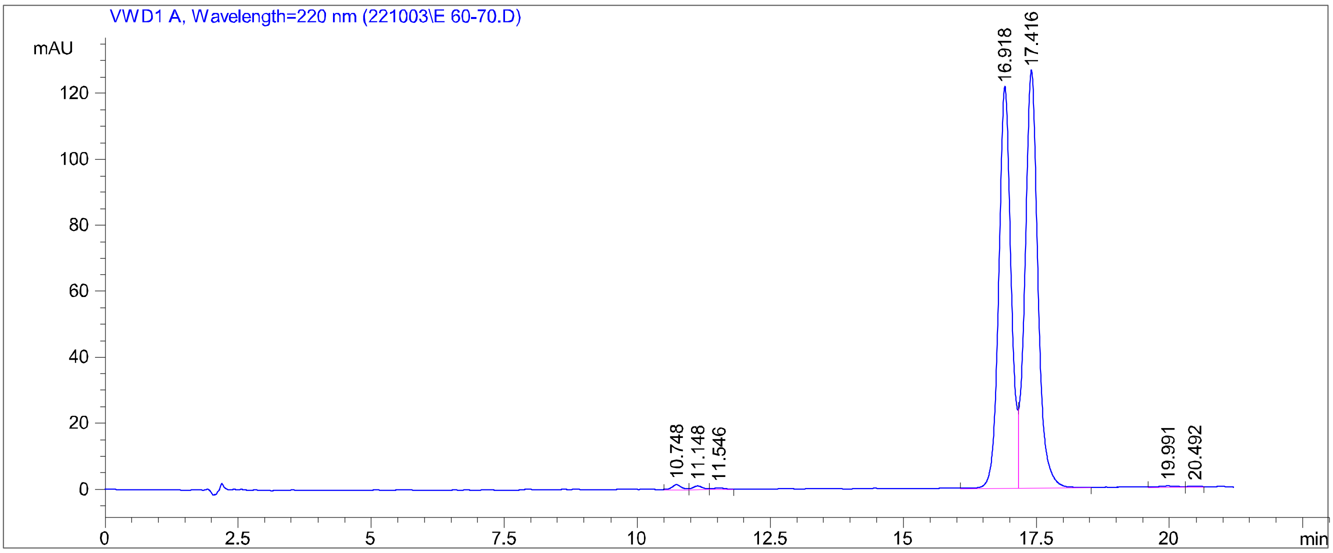

===

Area Percent Report

===

Sorted By : Signal
Multiplier : 1.0000
Dilution : 1.0000
Use Multiplier & Dilution Factor with ISTDs

Signal 1: VWD1 A, Wavelength=220 nm

Peak #	RetTime [min]	Type	Width [min]	Area mAU *s	Height [mAU]	Area %
1	10.748	MF	0.1911	16.90440	1.47459	0.3978
2	11.148	MF	0.2049	13.03335	1.06039	0.3067
3	11.546	FM	0.2292	6.50609	4.73162e-1	0.1531
4	16.918	MF	0.2655	1991.02295	124.98219	46.8574
5	17.416	FM	0.2833	2210.45190	130.05444	52.0215
6	19.991	MM	0.3865	8.29231	3.57625e-1	0.1952
7	20.492	MM	0.2156	2.90310	2.24429e-1	0.0683

Totals : 4249.11410 258.62682

===

*** End of Report ***

Analysis Name: IA-1670
Method: CHROM_ADC_PN.M
Sample Name: Default
Analysis Info: Column: ReprosilPur Basic C18 250*4.6 mm 5um
A: H2O 0,01% tfa B: MeCN 0,01% tfa
5-100 %B at 20min
Instrument: LC-MSD-Trap-SL
Operator: LCMS

Print Date: 9/30/2022 11:49:09 AM
Acq. Date: 9/29/2022 5:35:20 PM

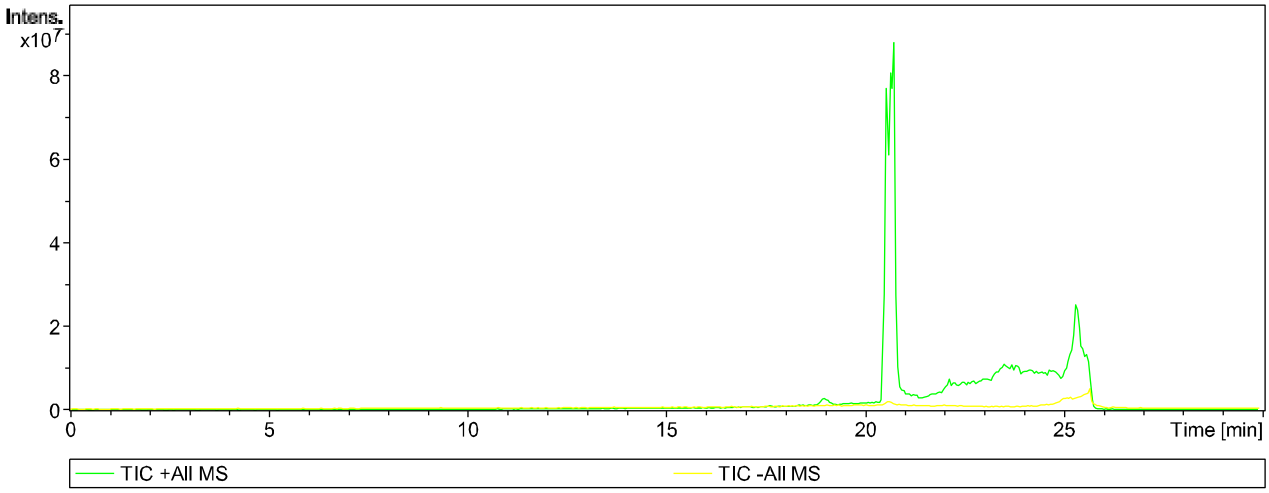

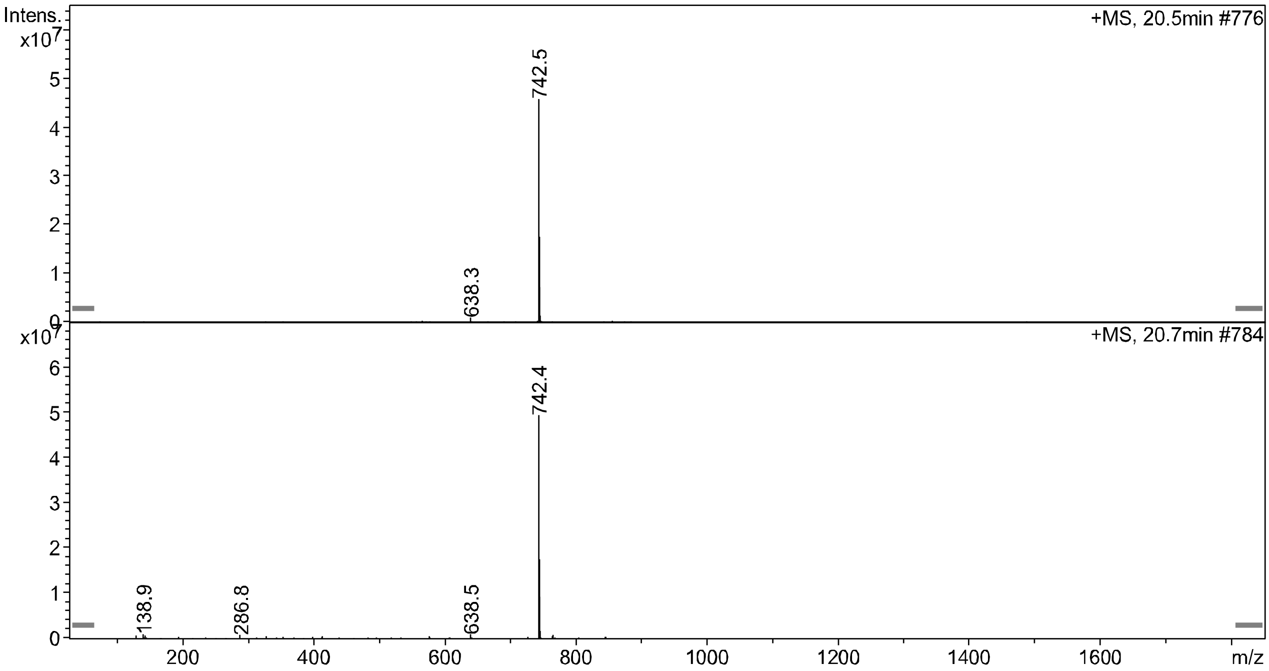

Supplement: Supplementary file 1 [file ijms-24-16359-s001.zip › ijms-2693112-supplementary.pdf]
